# Stafib‐2‐CR: an Improved Nanomolar and Selective Inhibitor of the Transcription Factor STAT5b Developed by Conformational Restriction of Stafib‐2

**DOI:** 10.1002/chem.202502809

**Published:** 2025-11-30

**Authors:** Theresa Münzel, Angela Berg, Christoph Protzel, Sylvie Schäfer, Alexander Jensen‐Feinhals, Thorsten Berg

**Affiliations:** ^1^ Institute of Organic Chemistry Leipzig University Leipzig Germany

**Keywords:** biological activity, cyclization, inhibitors, protein‐protein interactions, transcription factors

## Abstract

The highly homologous transcription factors STAT5a and STAT5b are overactivated in many human tumor types. We recently reported catechol bisphosphates as the first chemical entities that inhibit STAT5b with selectivity over STAT5a. Here, we apply conformational restriction strategies to increase the activity and selectivity of Stafib‐2, the most potent STAT5b inhibitor reported to date. The best conformationally restricted Stafib‐2 analogue **8b** (dubbed Stafib‐2‐CR) displayed approximately threefold higher activity against STAT5b than Stafib‐2, associated with higher selectivity over STAT5a. Its cell‐permeable prodrug **17** (dubbed Pomstafib‐2‐CR) inhibits phosphorylation of STAT5b in cultured human leukemia cells with slightly higher activity and selectivity over STAT5a than Pomstafib‐2, the prodrug corresponding to Stafib‐2.

## Graphical abstract

Conformational restriction strategies to increase the activity and selectivity of the STAT5b inhibitor Stafib‐2 are presented. The best conformationally restricted inhibitor Stafib‐2‐CR has threefold higher activity against STAT5b than Stafib‐2. A cell‐permeable prodrug of Stafib‐2‐CR inhibits phosphorylation of STAT5b in cultured human leukemia cells with higher activity and selectivity than the prodrug corresponding to Stafib‐2.

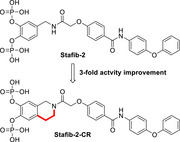

## 1 Introduction

Signal transducers and activators of transcription (STATs) are latent cytoplasmic transcription factors, which mediate cell signaling upon ligand‐induced activation of cytokine or growth factor receptors [1]. All seven members of the protein family are involved in important biological processes [2]. The family members STAT5a and STAT5b share 96 % homology on the amino acid level [3, 4], and possess both redundant and nonredundant functions [5, 6]. STAT5a and STAT5b are constitutively activated in many human tumor types, including Bcr‐Abl induced chronic myelogenous leukemia (CML) [7]. However, STAT5b appears to be the main determinant of cell growth and tumorigenesis [8, 9, 10, 11, 12, 13]. Chemical agents that inhibit one STAT5 protein with selectivity over the other are highly desirable as tools to investigate the individual roles of STAT5a/5b for various cellular processes [14].

**FIGURE 1:**
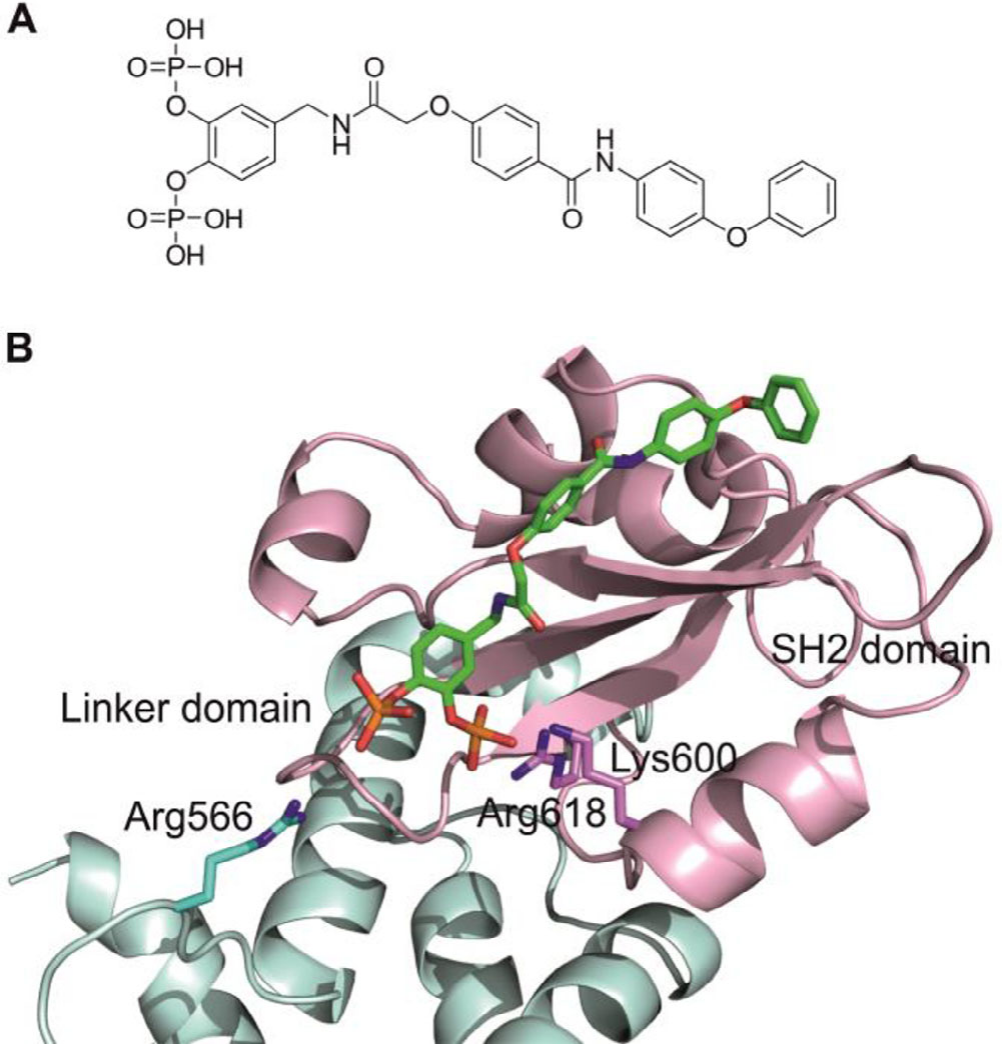
(A) Structure of Stafib‐2 and (B) its binding mode to STAT5b as previously proposed [18]. The figure was generated using PyMOL [19].

We recently presented catechol bisphosphates as the first chemical entities which inhibit STAT5b with selectivity over STAT5a [15, 16], with Stafib‐2 as the most potent STAT5b inhibitor published to date (Figure 1A) [17]. The source of the selectivity of catechol bisphosphates for STAT5b over STAT5a is the electrostatic interaction between one of the phosphoryl groups and the side chain of Arg566 in the STAT5b linker domain (Figure 1B) [18]. STAT5a carries a tryptophan residue in position 566, which cannot engage in similar attractive interactions.

## 2 Results and Discussion

In an effort to further increase the activity of Stafib‐2, we applied a conformational restriction approach [20, 21]. Favorable conformational preorganization reduces the entropic penalty upon binding of the target protein, leading to higher affinity and possibly higher selectivity.

**FIGURE 2:**
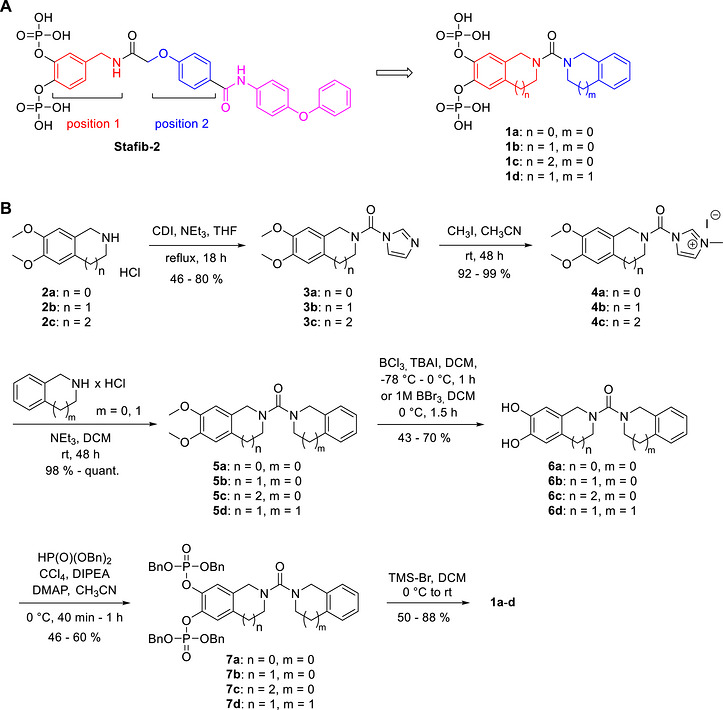
(A) Design concept and (B) synthesis of ureas **1a**–**d**.

Our conformational restriction approach is based on the introduction of 5‐ to 7‐membered heterocyclic rings into the structure of Stafib‐2, whilst retaining the existing structural elements to the maximum extent possible. One obvious possibility to implement this approach is to replace the benzylamine motif (position 1, Figure 2A) by an isoindoline, a tetrahydroisoquinoline, or a 2,3,4,5‐tetrahydro‐1*H*‐benzo[*c*]azepine motif. Another possibility is to replace the aromatic ether functionality (position 2) with *N*‐heterocycles such as isoindoline or tetrahydroisoquinoline. Initially, isoindoline was chosen as the heterocycle in position 2, resulting in ureas **1a**‐**c**. In one example (**1d**), we also used tetrahydroisoquinoline in position 2. This was carried out in combination with the tetrahydroisoquinoline moiety in position 1, as the demethylation procedure leading to catechol **6b** worked comparatively well. The hydrophobic phenoxyphenyl part of Stafib‐2 was omitted from the target molecules for practical reasons.

Synthesis of ureas **1a**–**d** started from catechol ethers **2a**–**c** (Figure 2B). Reaction with 1,1’carbonyldiimidazole generated carbamoylimidazoles **3a**–**c**, which were converted to the corresponding carbamoylimidazolium salts **4a**–**c** using methyl iodide. Reaction with isoindoline or tetrahydroisoquinoline, both used as hydrochlorides, resulted in formation of **5a**–**d**. Demethylation with BBr_3_ or BCl_3_ and tetra‐*n*‐butylammonium iodide (TBAI) provided the catechols **6a**–**d**, which were subjected to Atherton‐Todd phosphorylation [22]. The benzyl protecting groups of **7a**–**d** were removed by treatment with TMS‐Br, providing the target molecules **1a**–**d**.

In competitive binding assays based on fluorescence polarization (FP), the most potent of the synthesized ureas, compound **1c**, showed slightly more than twofold higher activity against STAT5b (IC_50_ = 0.78 ± 0.04 µM, Table 1) than catechol bisphosphate (CBP, IC_50_ = 1.79 ± 0.14 µM) [18]. In the presence of a tetrahydroisoquinoline moiety in position 1, the exchange of the isoindoline motif in position 2 (compound **1b,** IC_50_ = 1.29 ± 0.15 µM) for a tetrahydroisoquinoline (**1d,** IC_50_ = 1.20 ± 0.10 µM) did not have a significant effect on compound activities. Unfortunately, all of the ureas **1a**–**d** showed lower selectivity for STAT5b over STAT5a (10‐18‐fold) than catechol bisphosphate (38‐fold).

**TABLE 1:** Structures and activities of rigidified STAT5b inhibitors **1a**–**d** against STAT5a and STAT5b in FP assays. Mean values ± standard deviations are given (*n* = 3). CBP: Catechol bisphosphate.

No/ name	Structure	STAT5b IC_50_ (µM)	STAT5a IC_50_ (µM)	Selectivity IC_50_ (STAT5a) / IC_50_ (STAT5b)
**CBP**	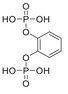	1.79 ± 0.14^[a]^	67.8 ± 7.6^[a]^	38
**1a**	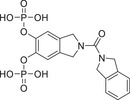	1.14 ± 0.12	11.9 ± 0.97	10
**1b**	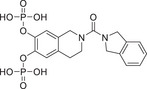	1.29 ± 0.15	13.9 ± 2.93	11
**1c**	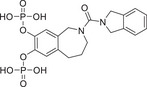	0.78 ± 0.04	14.2 ± 1.72	18
**1d**	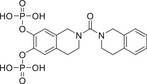	1.20 ± 0.10	18.1 ± 1.43	15

^[a]^ Data taken from the literature [18].

**FIGURE 3:**
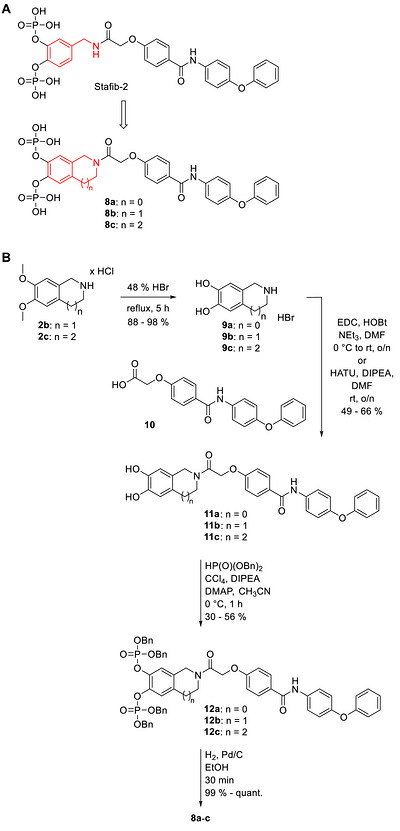
(A) Design concept and (B) synthesis of rigidified Stafib‐2 derivatives **8a**–**c**. Compound **9a** was commercially available.

It is conceivable that the geometry and rigidity of the urea moiety of **1a**–**d** placed the two heterocycles in positions 1 and 2 in an orientation which is unfavorable for protein binding. We therefore applied conformational restriction only at the benzylamine position 1 of Stafib‐2 by introducing an isoindoline, a tetrahydroisoquinoline, or a 2,3,4,5‐tetrahydro‐1*H*‐benzo[*c*]azepine moiety. The aromatic ether functionality of Stafib‐2 (position 2, Figure 2A) was left unchanged, thus avoiding the generation of a urea functionality in the target molecules **8a**–**c** (Figure 3A). To this end, dimethoxy ethers of tetrahydroisoquinoline (**2b**) and tetrahydrobenzoazepine (**2c**) were cleaved using 48 % HBr (Figure 3B). Coupling of the resulting catechols **9b**,**c** as well as of commercially available isoindoline catechol **9a** with the carboxylic acid **10** [17] produced **11a**–**c**, which were subjected to Atherton‐Todd phosphorylation to obtain the benzyl‐protected phosphorylated compounds **12a**–**c**. Hydrogen‐mediated cleavage of the benzyl ester functionalities provided **8a**–**c** in an overall yield of 20‐23 % over three to four steps.

**FIGURE 4:**
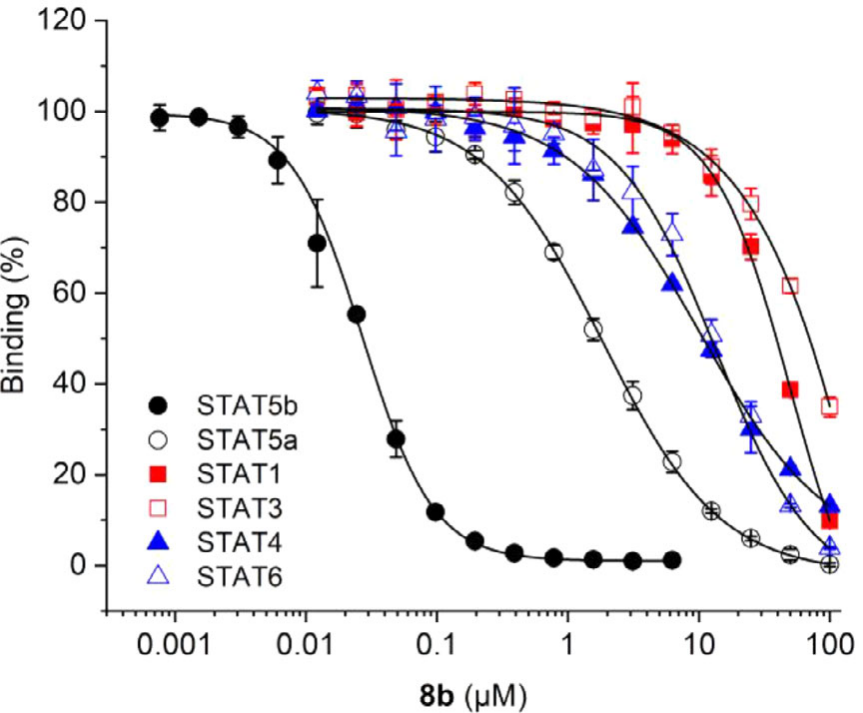
Selectivity profile of **8b** in FP assays against STAT proteins. Error bars represent standard deviations (*n* = 3).

Activity analysis in FP assays indicated that introduction of the isoindoline moiety (compound **8a**) had a negative effect on STAT5b inhibition (IC_50_ = 0.162 ± 0.004 µM, Table 2) as compared to the parent molecule Stafib‐2 (IC_50_ = 0.068 ± 0.003 µM) [18]. In contrast, the conformationally restricted Stafib‐2 derivative containing the tetrahydroisoquinoline moiety (**8b**, dubbed Stafib‐2‐CR) was 2‐3‐fold more active against STAT5b (IC_50_ = 0.026 ± 0.001 µM, Table 2) than Stafib‐2, which was associated with twofold higher selectivity over STAT5a (69‐fold for **8b** compared to 29‐fold for Stafib‐2, Table 2), and even higher selectivity against the other STAT proteins (∼400‐2700‐fold, Figure 4, Table S1) [18]. The tetrahydrobenzoazepine derivative **8c** (IC_50_ = 0.034 ± 0.004 µM, Table 2) was also more potent than Stafib‐2 and thus only slightly less potent than the tetrahydroisoquinoline derivative **8b**, whilst being even more selective for STAT5b over STAT5a (147‐fold). Replacement of the ether functionality of **8b** by a secondary amine, as represented by **13** (Scheme S1), to possibly allow for additional conformational stabilization between the amine and the carbonyl group via an intramolecular hydrogen bond, was unfavorable (IC_50_ = 0.078 ± 0.004 µM, Table 2).

**TABLE 2:** Structures and activities of rigidified STAT5b inhibitors against STAT5a and STAT5b in FP assays. Mean values ± standard deviations are given (*n* = 3).

No	Structure	STAT5b IC_50_ (µM)	STAT5a IC_50_ (µM)	Selectivity IC_50_ (STAT5a) / IC_50_ (STAT5b)
Stafib‐2	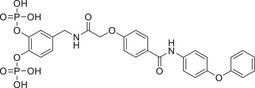	0.068 ± 0.003^[^ a ^]^	1.97 ± 0.09^[^ a ^]^	29
**8a**	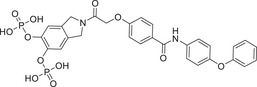	0.162 ± 0.004	4.52 ± 0.17	28
**8b** (Stafib‐2‐CR)	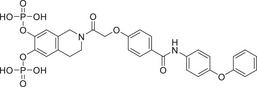	0.026 ± 0.001	1.79 ± 0.17	69
**8c**	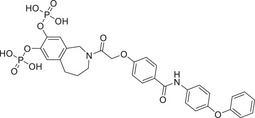	0.034 ± 0.004	4.99 ± 0.18	147
**13**	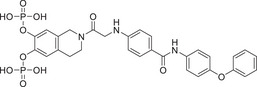	0.078 ± 0.004	2.05 ± 0.35	26
**14**	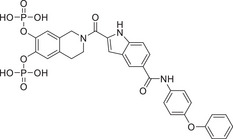	0.112 ± 0.003	3.31 ± 0.19	30
**15**	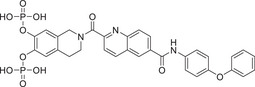	0.130 ± 0.008	4.78 ± 0.82	37
**16**	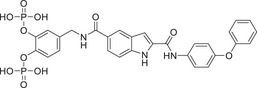	0.079 ± 0.004	0.97 ± 0.20	12

^[a]^ Data taken from the literature [18].

Since the tetrahydroisoquinoline motif was best suited for the replacement of the benzylamine motif in position 1, we tried to introduce additional conformationally restricted elements in the position of the aryl ether (position 2) without generating a urea functionality. However, neither compound **14** (Scheme S2) containing an indole moiety (IC_50_ = 0.112 ± 0.003 µM, Table 2) nor the quinoline derivative **15** (IC_50_ = 0.130 ± 0.008 µM, Scheme S3, Table 2) provided increased activities as compared to **8b**. Similarly, replacing the aromatic ether functionality of unconstrained Stafib‐2 (lacking the tetrahydroisoquinoline motif) with an indole (compound **16**) did not improve the activity against STAT5b (IC_50_ = 0.079 ± 0.004 µM, Scheme S4, Table 2). In summary, **8b** (IC_50_ = 0.026 ± 0.001 µM, Table 2) was the most potent conformationally restricted inhibitor in this series of Stafib‐2 derivatives and also exhibited higher selectivity over STAT5a (69‐fold) than Stafib‐2 (29‐fold). While the tetrahydrobenzoazepine derivative **8c** was even more selective for STAT5b over STAT5a (147‐fold), it was slightly less potent (IC_50_ = 0.034 ± 0.004 µM, Table 2) than **8b**. Since the primary aim of this study was to improve the activity of Stafib‐2, we selected **8b** for further analysis.

**FIGURE 5:**
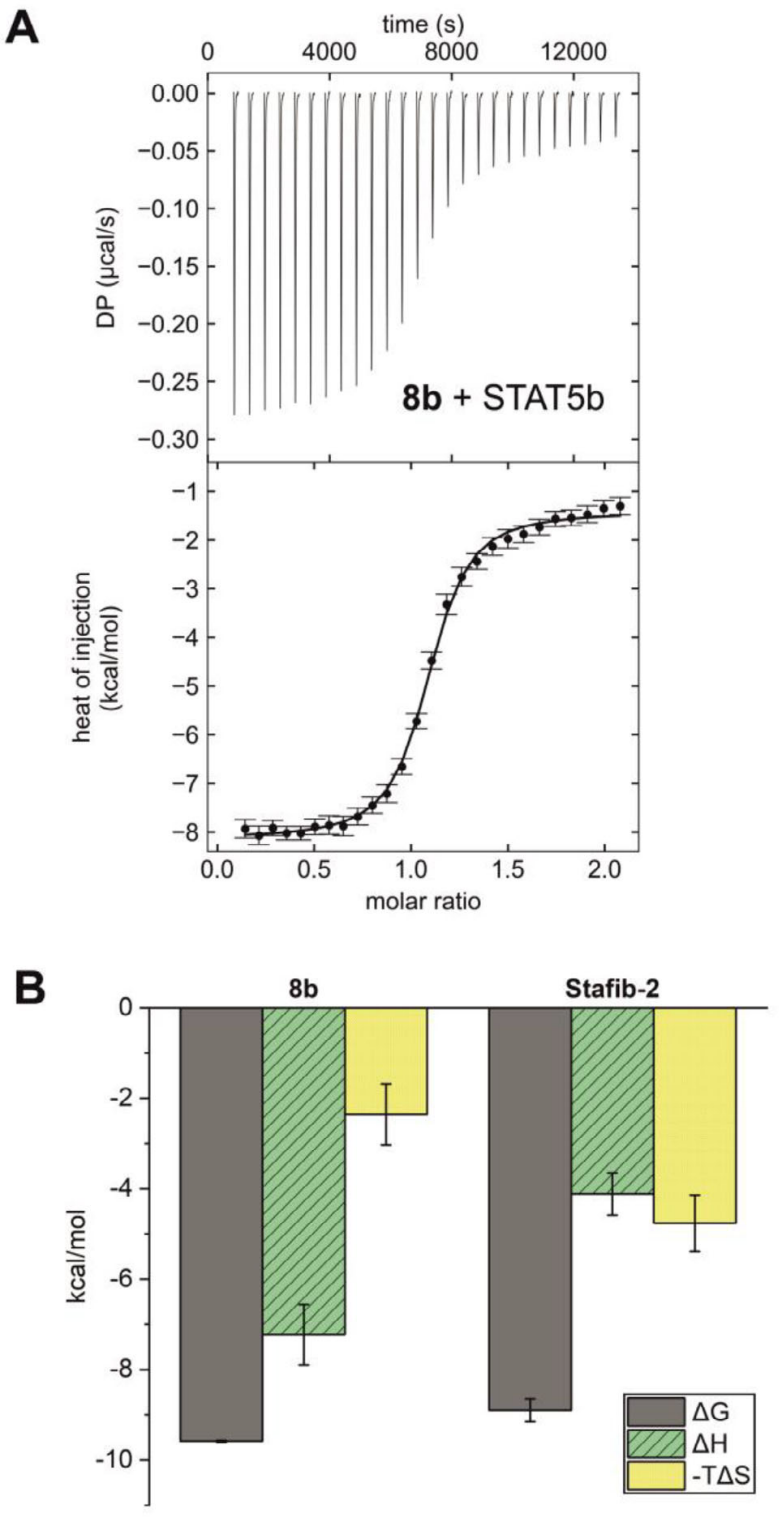
(A) Thermogram of **8b** binding to STAT5b by ITC. Error bars represent integration errors assigned by the data analysis software NITPIC for the depicted individual experiment [23] (B) Analysis of thermodynamic parameters for STAT5b binding of **8b** and Stafib‐2 [17] Mean values ± standard deviations are given (*n* = 3).

In isothermal titration calorimetry, **8b** bound to STAT5b (K_d_ = 0.094 ± 0.003 µM, Figure 5A) with three‐ to fourfold higher affinity than previously determined for Stafib‐2 (K_d_ = 0.32 ± 0.11 µM) [17], consistent with the results of the FP assays (Table 2). No heat was generated when **8b** was titrated into the corresponding buffer (Figure S1). Interestingly, although the increased affinity of **8b** was supposed to be achieved by a lower entropic penalty upon protein binding, analysis of the thermodynamic parameters revealed that it was in fact mainly due to enthalpic effects (Figure 5B). While a possible explanation would involve the formation of more stable hydrogen bonding with the protein conferred by the rigidified compound, cocrystal structure analysis between STAT5b and **8b** as well as Stafib‐2 would be required to elucidate the origin of this observation with certainty.

**FIGURE 6:**
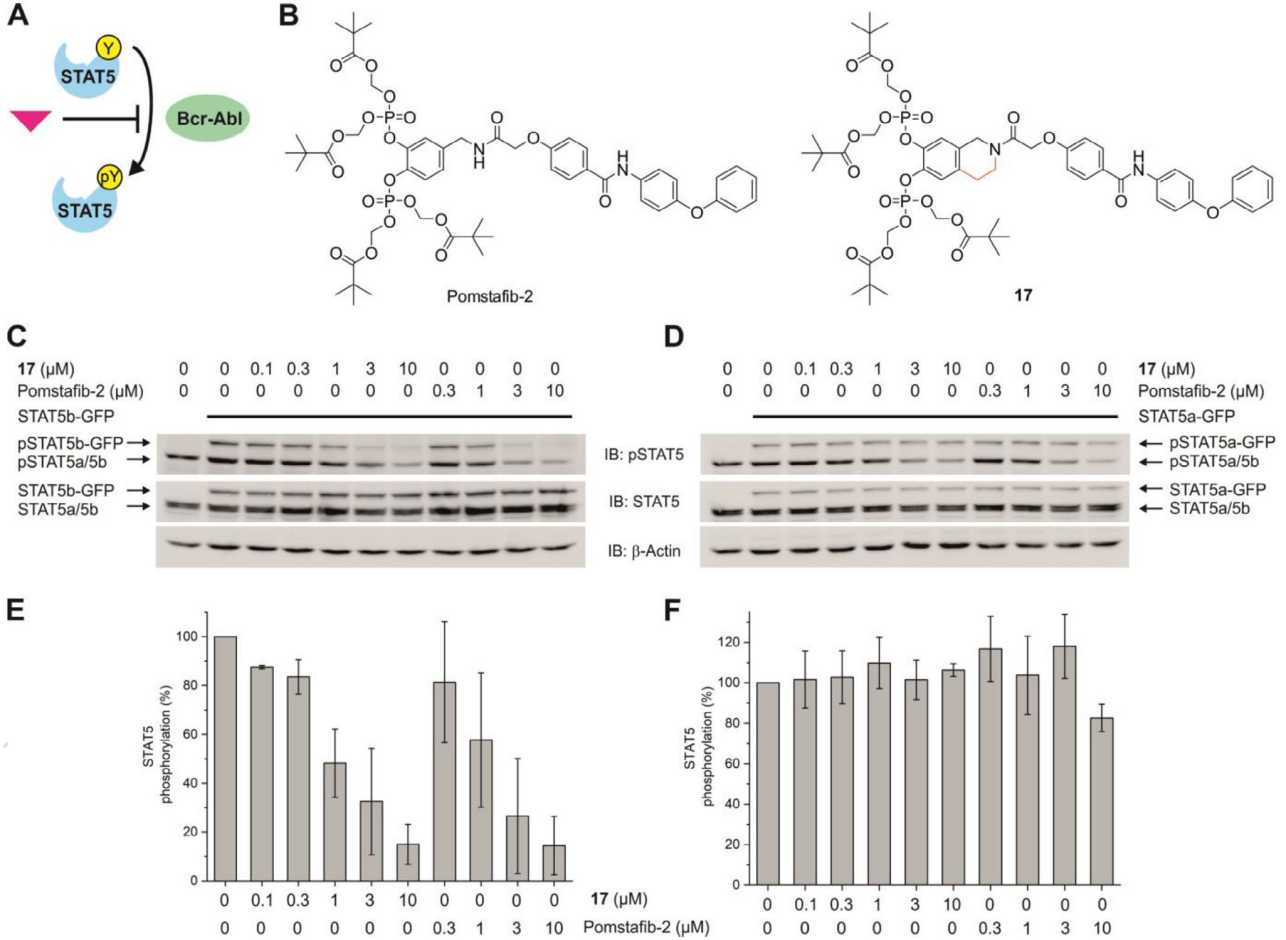
(A) Tyrosine phosphorylation of STAT5 proteins by Bcr‐Abl can be inhibited by ligands of the SH2 domain, symbolized by the magenta triangle. (B) Structures of Pomstafib‐2 and **17**. (C) Treatment of transfected K562 cells with Pomstafib‐2 or **17** leads to inhibition of phosphorylation of STAT5b‐GFP on Tyr699, but (D) not of STAT5a‐GFP on Tyr694. Phosphorylation of endogenous STAT5a/5b, which cannot be distinguished by the phosphorylation‐dependent STAT5 antibody, is also reduced by Pomstafib‐2 and **17**. (E) Quantification of the intensities of the pSTAT5b‐GFP band and F) the pSTAT5a‐GFP bands shown in (C) and (D), respectively (*n* = 3). pSTAT5a/b‐GFP levels are normalized against total STAT5a/b‐GFP. Error bars indicate standard deviations.

In human K562 leukemia cells, the nonreceptor tyrosine kinase Bcr‐Abl induces constitutive phosphorylation of STAT5 proteins on a conserved tyrosine residue (STAT5a: Tyr694; STAT5b: Tyr699) C‐terminal of the SH2 domain (Figure 6A) [15]. Blocking of the SH2 domain prevents tyrosine phosphorylation, which can be detected by a phospho‐specific antibody which recognizes STAT5a and STAT5b only in their tyrosine phosphorylated state. We developed an assay by which to distinguish the effect of an inhibitor on STAT5a or STAT5b alone, based on transfection of K562 cells with fusion constructs of either STAT5a or STAT5b with GFP. The ectopic product of either construct has a significantly higher molecular weight than the wild‐type proteins [15].

Catechol bisphosphates are negatively charged at physiological pH, which hampers their ability to cross the cell membrane by passive diffusion. Consistent with this, we had previously found that Stafib‐2 does not affect STAT5b phosphorylation in K562 cells [17]. However, cellular activity could be obtained by masking of its phosphate groups as pivaloyloxymethyl (POM) esters [24], which are cleaved off by intracellular phosphatases, in the prodrug Pomstafib‐2 (Figure 6B) [17]. Therefore, **8b** was converted to its POM‐ester **17** dubbed Pomstafib‐2‐CR (Figure 6B).

The conformationally constrained prodrug **17** inhibited phosphorylation of STAT5b‐GFP with an IC_50_ of 1.0 µM (Figures 6C, E, and S2A). This represents a slight, albeit not statistically significant, improvement over the activity of Pomstafib‐2 (IC_50_ = 1.6 µM, Figures 6D, F, and S2B), which was re‐tested side‐by‐side in these experiments. Phosphorylation of STAT5a‐GFP was not inhibited to a noticeable extent by **17** (Figure D, F), and slightly by Pomstafib‐2 at 10 µM, the highest concentration tested. The improved activity and selectivity of **17** compared to Pomstafib‐2 in the cell‐based assays is consistent with the results of the FP assays of **8b** and Stafib‐2 (Table 2). Presumably, the lower degree of improvement in the cell‐based assays is related to the overall lower activity of the prodrugs compared to the unprotected compounds **8b** and Stafib‐2 used in the in vitro assays (Table 2, Figure 5).

## 3 Conclusion

In summary, we have explored conformational restriction strategies aimed at improving the potency of the STAT5b inhibitor Stafib‐2. By replacing the benzylamine motif of Stafib‐2 with a tetrahydroisoquinoline moiety, the conformationally restricted STAT5b inhibitor **8b** (Stafib‐2‐CR) was created, which exhibits approximately three times higher activity than Stafib‐2 in FP assays and ITC. Data from the FP assays indicate that this was associated with a higher selectivity of 69‐fold toward STAT5a, compared to 29‐fold for Stafib‐2. In K562 human leukemia cells, the prodrug **17** (Pomstafib‐2‐CR) based on **8b** inhibited the phosphorylation of a STAT5b‐GFP fusion protein with slightly higher potency than Pomstafib‐2, associated with slightly higher selectivity over STAT5a‐GFP compared to Pomstafib‐2. Stafib‐2‐CR and its prodrug Pomstafib‐2‐CR are currently the most potent selective small‐molecule inhibitors of STAT5b.

## Conflicts of Interest

The authors declare no competing interest.

## Supporting information




**Supporting file**: The authors have cited additional references within the Supporting Information [25, 26, 27, 28, 29, 30, 31, 32, 33, 34, 35, 36, 37, 38, 39].

## Data Availability

The data underlying this study are available in the published article and online Supporting Information.
