# Supplementary material for: Stafib‐2‐CR: an Improved Nanomolar and Selective Inhibitor of the Transcription Factor STAT5b Developed by Conformational Restriction of Stafib‐2
Source: Chemistry. 2025 Nov 30;32(26):e02809. doi: 10.1002/chem.202502809 (PMC13356315; doi:10.1002/chem.202502809)
Supplement: Supplementary file 1 — Supporting file: The authors have cited additional references within the Supporting Information [25, 26, 27, 28, 29, 30, 31, 32, 33, 34, 35, 36, 37, 38, 39]. [file CHEM-32-e02809-s001.pdf]

Supporting Information

Stafib-2-CR: an Improved Nanomolar and Selective Inhibitor of the Transcription Factor STAT5b Developed by Conformational Restriction of Stafib-2

Theresa Münzel,[a] Angela Berg,[a] Christoph Protzel,[a] Sylvie Schäfer,[a] Alexander Jensen-Feinhals,[a] and Thorsten Berg*[a]

Table of Contents

Table S1 2

Figure S1 2

Figure S2 3

Scheme S1 3

Scheme S2 4

Scheme S3 4

Scheme S4 5

Methods 5

Plasmids, protein expression and purification 5

Fluorescence polarization assays 5

Isothermal titration calorimetry 6

Cell culture and transfection 6

Western blotting 7

NMR spectroscopy 7

General synthetic methods 8

Synthesis and spectroscopic characterization of compounds 10

NMR spectra 54

Supporting references 72

Table S1: Activity of **8b** against STATs in competitive FP assays. Mean values ± standard deviations are given (n = 3).

No	Structure	STAT1 IC_{50} (µM)	STAT3 IC_{50} (µM)	STAT4 IC_{50} (µM)	STAT5a IC_{50} (µM)	STAT5b IC_{50} (µM)	STAT6 IC_{50} (µM)
**8b**	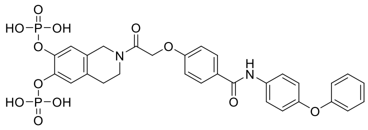	39.9 ± 1.0	71.2 ± 3.8	10.6 ± 0.8	1.79 ± 0.17	0.026 ± 0.001	13.4 ± 1.0

Figure S1

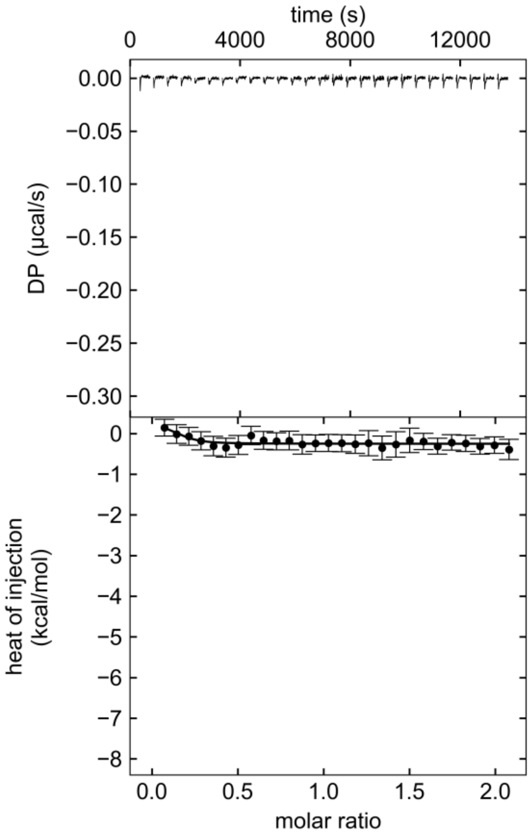

Figure S1: Titration of 100 µM **8b** dissolved in ITC buffer to a final DMSO concentration of 2 % (v/v) into ITC buffer supplemented with 2 % (v/v) DMSO.

Figure S2

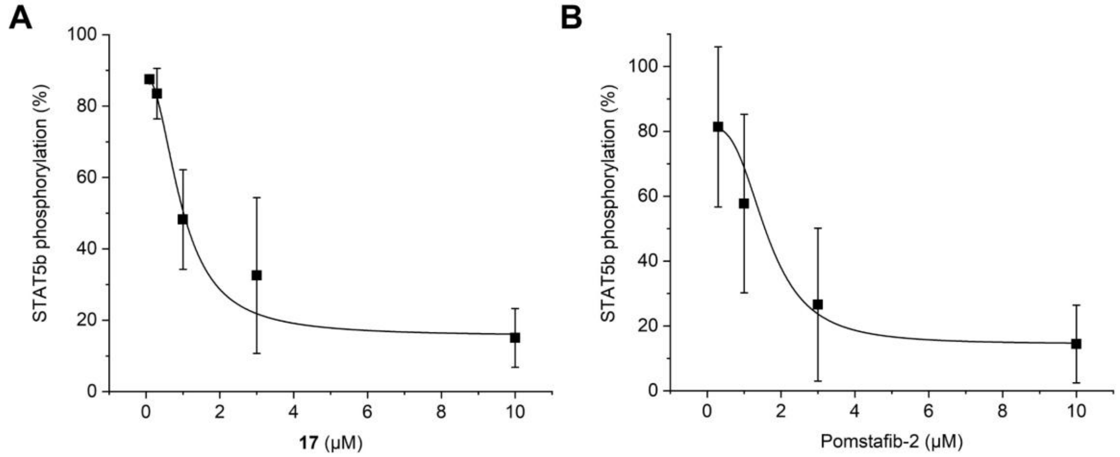

Figure S2: Comparison of the activities of the prodrugs A) **17** (IC_{50} = 1.0 μM) and B) Pomstafib-2 (IC_{50} = 1.6 μM) against STAT5b phosphorylation in STAT5b-GFP-transfected K562 cells. The IC_{50} of Pomstafib-2 in these experiments had been determined as 1.5 μM in the original publication.[1] Error bars indicate standard deviations (*n* = 3).

Scheme S1

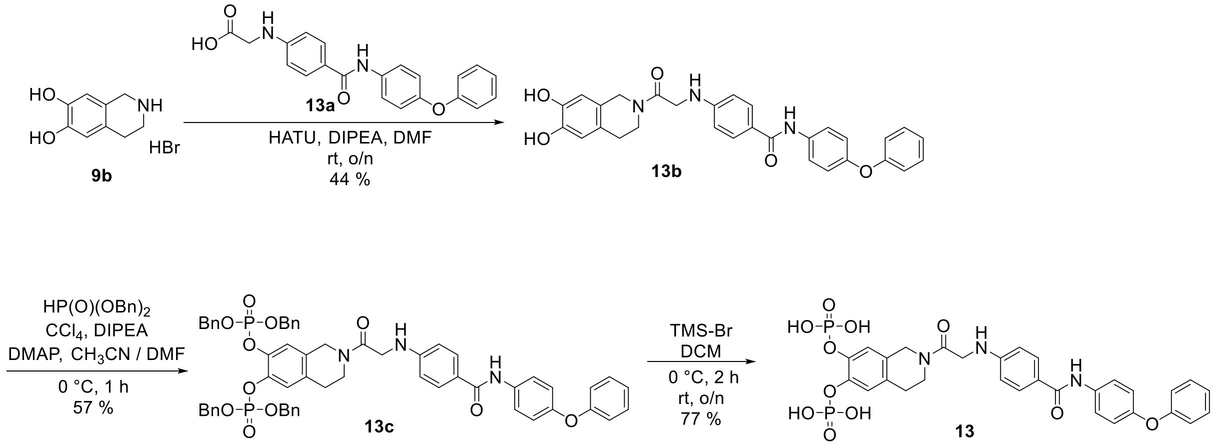

Scheme S1: Synthesis of **13**.

Scheme S2

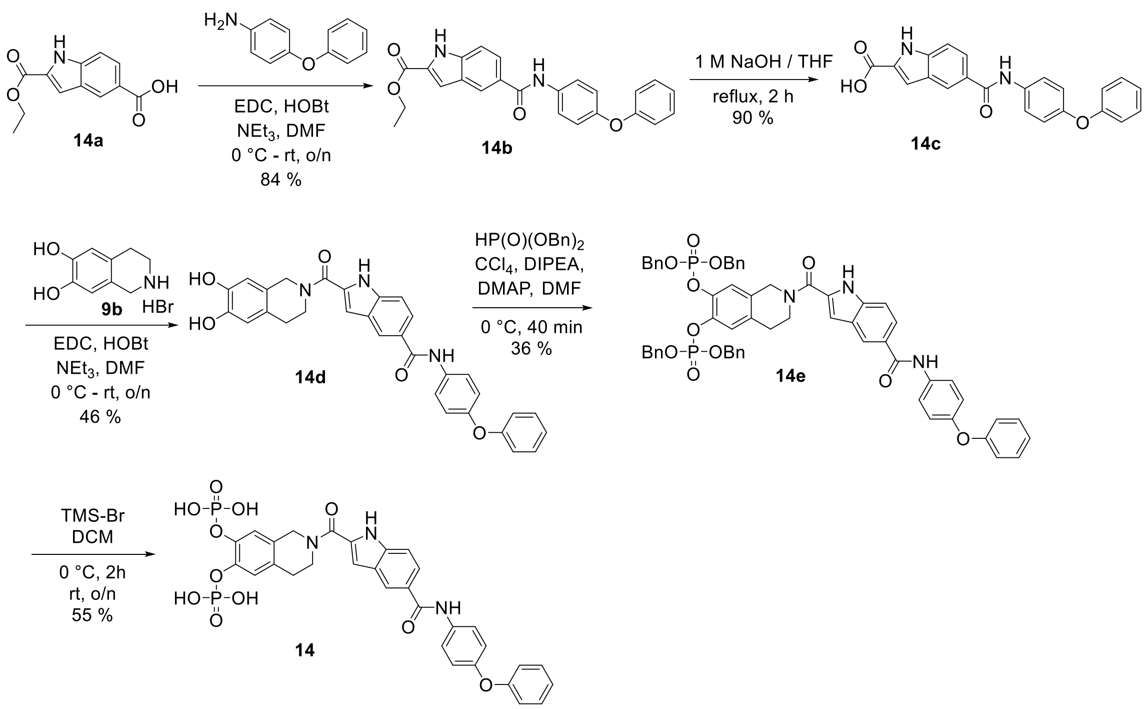

Scheme S2: Synthesis of **14**.

Scheme S3

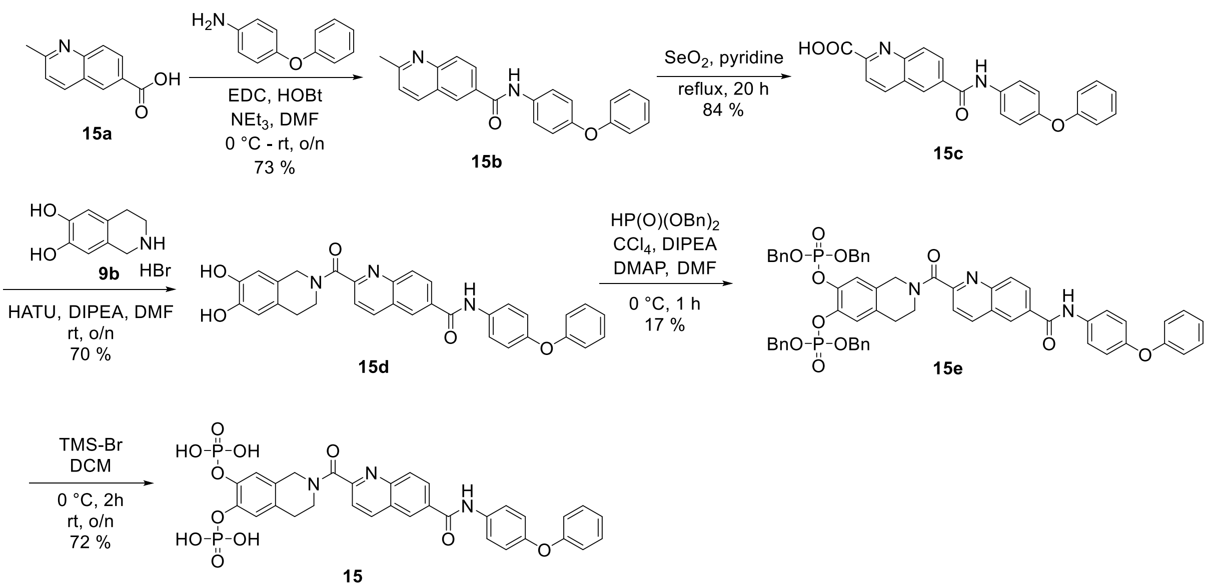

Scheme S3: Synthesis of **15**.

Scheme S4

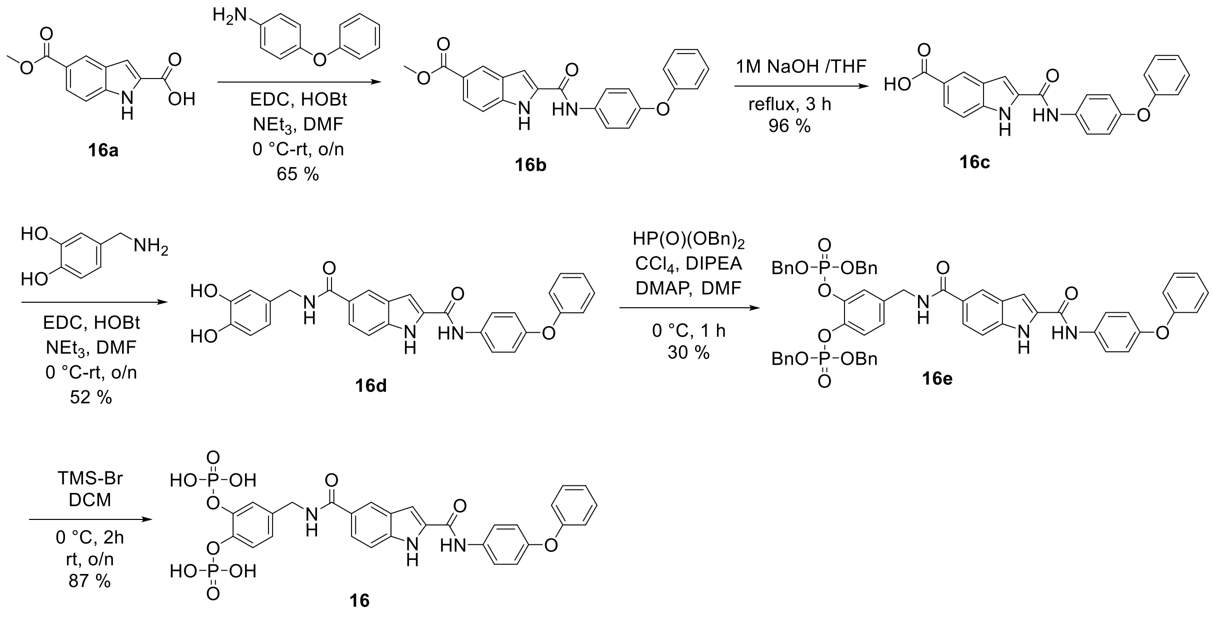

Scheme S4: Synthesis of **16.**

Methods

Plasmids, protein expression and purification

Expression constructs encoding STAT1,[2] STAT3,[3] STAT4,[4] STAT5a,[5] STAT5b[6] and STAT6[4] have been described. Protein expression and purification have also been described.[7] Proteins were purified via affinity chromatography using His-Bind resin (Millipore). Proteins used for fluorescence polarization assays were purified twice by affinity chromatography on His-Bind resin. They were dialyzed against 100 mM NaCl, 50 mM HEPES pH 7.5, 1mM EDTA, 1mM DTT, 10% (*v*/*v*) glycerol and 0.1% (*v*/*v*) NP-40, and snap frozen in liquid nitrogen. STAT5b used for ITC was purified over a single His-Bind resin column, and was dialyzed against a buffer containing 10 mM Tris pH 8.0 and 50 mM NaCl. Protein samples used for ITC were not frozen prior to measurement.

Cloning of the plasmids encoding GFP-STAT5a and GFP-STAT5b for expression in mammalian cells has also been described.[5]

Fluorescence polarization assays

The ability of a compound to displace a fluorescent-labelled peptide from the SH2 domain of the respective STAT protein was analyzed by competitive fluorescence polarization assays as described previously.[7] The following peptide sequences were used with a final concentration of 10 nM: 5-carboxyfluorescein-GpYDKPHVL for STAT1; 5-carboxyfluorescein-GpYLPQTV-NH_2 for STAT3, 5-carboxyfluorescein-GpYLPQNID for STAT4, 5-carboxyfluorescein-

GpYLVLDKW for STAT5a/5b, and 5-carboxyfluorescein-GpYVPWQDLI for STAT6. The final protein concentration was equivalent to the K_d-value determined in a fluorescence binding assay: 83 nM for STAT1, 81 nM for STAT3, 32 nM for STAT4, betweem 70 nM and 176 nM for STAT5a, 62 or 91 nM for STAT5b, and 85 nM for STAT6. The assays were carried out in FP-assay buffer, consisted of 10 mM Tris (pH 8.0), 50 mM NaCl, 1 mM DTT, 1 mM EDTA, 0.1 % Nonidet P-40 substitute and 2 % DMSO. A dilution series of the test compounds in water or DMSO was incubated with the respective protein for 60 min at room temperature, followed by the addition of fluorescent-labelled peptide. Samples were transferred to 384-well microtiter plates and fluorescence polarisation was measured 60 min after the addition of the fluorescent-labelled peptide. An excitation wavelength of 485 nm was used to detect the emission at 535 nm. Percent protein-peptide binding relative to control samples without test compound (positive control) or without protein and test compound (negative control) was calculated using a logarithmic curve fit (software: OriginPro 8G). Experiments were carried out in triplicates.

Isothermal titration calorimetry

The STAT5b protein was expressed from the same construct as the one used for the FP assays. The protein was dialyzed against ITC buffer (10 mM Tris pH 8.0, 50 mM NaCl) using dialysis tubing with a 50 kDa cut-off. ITC experiments were performed using a VP-ITC Micro Calorimeter (MicroCal). **8b** was dissolved in DMSO as a 5 mM stock and was diluted 1/50 in ITC buffer (final ligand concentration: 100 µM). 2 % (*v*/*v*) DMSO was added to the buffers containing the proteins and to the reference buffer in order to prevent errors caused by the detection of dilution heat. Proteins were degassed before the experiments using a ThermoVac sample degassing station. ITC experiments were carried out using the following conditions: 10 µM STAT5b, 100 µM **8b**, 28 injections with one 0.5 µL preinjection and 27 times 10 µL single injection volume, 150 s initial delay, 500 s spacing between injections, 20 µcal/s reference power, 220 rpm stirring at 25 °C. A low-noise integration approach was used for data analysis with NITPIC[8-9] and SEDPHAT.[10] A one-site binding model was used for data fitting. Figures were generated using GUSSI.[11] The experiment was carried out in triplicate.

Cell culture and transfection

K562 cells were cultured in RPMI 1640 medium containing 10% FBS, 2 mM L-glutamine and penicillin/streptomycin. 0.5 x 10^6 cells per well in 1 ml medium were transfected with either STAT5a-GFP or STAT5b-GFP plasmid in a 24-well plate, using Fugene HD transfection reagent (Promega). After 24 h, cells were treated with test compound or DMSO for 4 h (final DMSO concentration 0.2%). Cells were harvested by washing twice with ice-cold TBS, followed by resuspension in TBS supplemented with protease/phosphatase inhibitors (100 ng/ml aprotinin, 1 mM Na_3VO_4, 10 mM NaF, 1 mM PMSF) and lysed using three freeze-thaw

cycles with liquid nitrogen. Lysates were cleared by centrifugation at 20,000g for 25 minutes, 4°C.

Western blotting

The components of cell lysates were separated by SDS-PAGE using a 10% gel, transferred to nitrocellulose membrane (Bio-Rad) and detected using monoclonal rabbit primary antibodies (Cell Signaling), α-rabbit-HRP secondary antibody (Dako) and Pierce ECL Plus chemiluminescence reagent (Thermo Scientific). Bands were visualized using an ImageQuant system (GE Healthcare) and quantitated using ImageJ software (NIH).[12]

NMR spectroscopy

^{1}H, ^{13}C and ^{31}P-NMR spectra were recorded on Varian MERCURYplus 300, Varian MERCURYplus 400, Bruker AVANCE III HD 400 and Bruker Fourier 300 spectrometers. Chemical shifts (δ) are reported in parts per million (ppm), referenced to the deuteurated solvents as specified. Assignments were made based on HSQC, HMBC and COSY 2D-NMR spectra. The temperature dependend isomerization of the amide bonds can lead to a second set of signals due to the presence of two rotamers in a variable ratio. Rotamers were defined as R1 for the major rotamer and R2 for the minor rotamer. Signals for the individual rotamers can be broadened or doubled. In certain cases, spectra were recorded at temperatures above or below room temperature, which can result in signal coalescence or distingushable signals for R1 and R2. In cases where peak broadening led to a complete disappearance of the respective signal, 2D NMR experiments were consulted to identify the signals listed in the spectroscopic characterization of the respective compounds below.

General synthetic methods

Method 1: Synthesis of carbamoylimidazoles

Carbamoylimidazoles were prepared essentially as described by *Grzyb et al*.[13] In brief, *N*,*N*'-carbonyldiimidazole (1.1 eq.) and the corresponding amine (1.0 eq.) were suspended in dry THF (2 mL/mmol). In case the amine was used as the respective HCl salt, additional triethylamine was added (1.0 eq.), and the mixture was refluxed for 18 h. Removal of the solvent gave an oil, which was dissolved in DCM and washed with water. The aqueous phase was extracted with DCM (3 x). The combined organic layers were dried over Na_2SO_4, filtered and concentrated in vacuo.

Method 2: Synthesis of carbamoylimidazolium salts

Carbamoylimidazolium salts were prepared essentially as described by *Grzyb et al*.[13] In brief, to a solution of carbamoylimidazole (1.0 eq.) in dry acetonitrile (2 mL/mmol) was added methyl iodide (4.0 eq.). The mixture was stirred at rt for 48 h, followed by removal of the solvent in vacuo to yield the carbamoylimidazolium salt.

Method 3: Synthesis of ureas

Ureas were prepared essentially as described by *Grzyb et al*.[13] In brief, to a solution of carbamoylimidazolium salt (1.0 eq.) in dry DCM (10 mL/mmol) was added either isoindoline hydrochloride (1.0 eq.) and triethylamine (2.0 eq.) or tetrahydroisoquinoline (1.0 eq.) and triethylamine (1.0 eq.). The resulting solution was stirred at rt for 48 h. Afterwards the reaction mixture was washed with 1 M HCl (2 x) and brine (1 x). The organic layer was dried over Na_2SO_4, filtered and concentrated in vacuo.

Method 4: Demethylation

Demethylation was carried out essentially as described by *Brooks et al*.[14] In brief, to a solution of methoxy-protected urea (1.0 eq.) and TBAI (5.5 eq.) in dry DCM (5 mL/mmol), BCl_3 (1 M solution in DCM, 5.5 eq.) was added dropwise at – 78 °C. After 5 min, the solution was warmed to 0 °C and was stirred for 1 h. Afterwards, the reaction mixture was quenched by the addition of methanol at 0 °C, stirred for another 30 min and the solvent was removed under reduced pressure.

Method 5: Atherton-Todd phosphorylation

To a solution of urea or amide (1.0 eq.) in dry acetonitrile (0.1 mmol/mL) was added CCl_4 (10 eq.), DIPEA (4.0 eq.) and catalytic amounts of DMAP. The resulting mixture was cooled to 0 °C. After the addition of dibenzyl phosphite (3.0 eq.), the mixture was stirred at 0 °C for

40 min – 1 h (TLC control). Upon completion of the reaction, KH_2PO_4 (0.5 M) was added, and the resulting suspension was extracted with EtOAc (2 x). The combined organic layers were washed with brine (1 x) dried over Na_2SO_4, filtered, and concentrated in vacuo.

Method 6: Debenzylation by TMS-Br

To a stirred solution of dibenzylphosphate ester (1.0 eq.) in dry DCM (0.03 mmol/mL) was added bromotrimethylsilane (14 eq.) dropwise at 0 °C. After 2 h at 0 °C, the mixture was stirred at rt overnight. Afterwards, the reaction was quenched by the addition of methanol, and co-evaporated three times with methanol and DCM each.

Method 7: Amide coupling

7A: To a solution of the carboxylic acid (1.00 eq.) in dry DMF (2.5 mL/mmol) were added EDC-HCl (1.05 eq.) and HOBt (1.00 eq.) at 0 °C. The mixture was stirred for 30 min at 0 °C. Subsequently, triethylamine (6.00 eq.) and the corresponding amine (1.00 eq.) were added. The reaction mixture was stirred overnight at rt. Afterwards, the mixture was extracted with H_2O and EtOAc (3 x). The combined organic layers were dried over Na_2SO_4, filtered and concentrated in vacuo.

7B: To a solution of carboxylic acid (1.0 eq.) and the corresponding amine (1.0 eq.) in dry DMF (0.28 mmol/mL) were added HATU (1.1 eq.) and DIPEA (5.0 eq.). The reaction mixture was stirred overnight at rt, followed by an extraction with EtOAc (3 x) and 5 % LiCl-solution (1 x). The combined organic phases were washed with brine (1 x), dried over Na_2SO_4 and concentrated in vacuo.

Method 8: Hydrogenation

To a stirring solution of benzyl-protected phosphates in ethanol was added Pd/C (10 wt. %) under N_2. The N_2 atmosphere was exchanged for H_2 and the mixture was stirred for 30 min (TLC control). Upon completion of the reaction, the mixture was filtered through celite or filter paper and washed with ethanol. The solvent was removed in vacuo.

Synthesis and spectroscopic characterization of compounds

(5,6-Dimethoxyisoindolin-2-yl)(1*H*-imidazol-1-yl)methanone (3a)

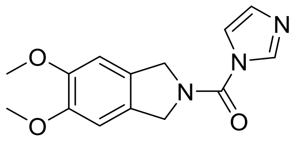

(5,6-Dimethoxyisoindolin-2-yl)(1*H*-imidazol-1-yl)methanone **3a** was prepared from 5,6-dimethoxyisoindolin hydrochloride **2a** (250 mg, 1.16 mmol, 1.0 eq.) according to **Method 1**. Additionally, triethylamine (166 µL, 1.20 mmol, 1.0 eq.) was added to the suspension. The crude product was purified by column chromatography (2 % MeOH in DCM) to yield **3a** as a colorless solid (195 mg, 62 %).

R_f = 0.28 (2 % MeOH in DCM, *v/v*).
^{1}H-NMR (400 MHz, $CDCl_3$): δ = 8.10 (t, *J* = 1.1 Hz, 1H), 7.44 (t, *J* = 1.5 Hz, 1H), 7.13 (t, *J* = 1.2 Hz, 1H), 6.77 (s, 2H), 4.95 (s, 4H), 3.88 (s, 6H) ppm.
^{13}C-NMR (101 MHz, $CDCl_3$): δ = 150.1, 149.8, 136.9, 129.9, 126.9, 117.8, 105.4, 56.3, 54.8 ppm.
HRMS (ESI, pos): *m/z* $[M+H]^+$ calculated for $[C_{14}H_{16}N_3O_3]^+$: 274.1186, found: 274.1180.
IR (KBr): $\tilde{\nu}$ = 3445 (br), 2937 (w), 1690 (s), 1508 (s), 1409 (s), 1357 (w), 1277 (m), 1226 (m), 1105 (m), 996 (w), 735 (w), 648 (w) cm^{-1}.
UV / Vis (DCM): λ_{max} = 287, 243 nm.
mp: 186 - 187 °C.

1-(5,6-Dimethoxyisoindoline-2-carbonyl)-3-methyl-1*H*-imidazol-3-ium iodide (4a)

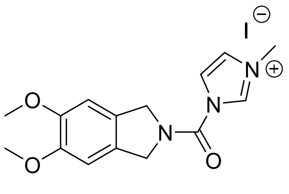

1-(5,6-Dimethoxyisoindoline-2-carbonyl)-3-methyl-1*H*-imidazol-3-ium iodide **4a** was prepared from **3a** (170 mg, 0.623 mmol, 1.0 eq.) according to **Method 2**. The solvent was removed in vacuo to yield the carbamoylimidazolium salt **4a** as a colorless solid (244 mg, 95 %).

^{1}**H-NMR** (300 MHz, DMSO-*d*$_6$, rotameric mixture 1:1): δ = 9.73 – 9.70 (m, 1H), 8.22 (t, *J* = 1.9 Hz, 1H), 7.91 – 7.89 (m, 1H), 7.05 (s, 1H), 6.90 (s, 1H), 4.93 (s, 2H), 4.88 (s, 2H), 3.95 (s, 3H), 3.76 (s, 3H), 3.74 (s, 3H) ppm.
^{13}**C-NMR** (75 MHz, DMSO-*d*$_6$, rotameric mixture 1:1): δ = 149.1, 146.0, 137.7, 126.9, 126.1, 123.5, 120.8, 106.2, 105.8, 55.7, 54.3, 36.4 ppm.
HRMS (ESI, pos): *m/z* $[M]^+$ calculated for $[C_{15}H_{18}N_3O_3]^+$: 288.1343, found: 288.1352.
IR (KBr): $\tilde{\nu}$ = 3576 (m), 3399 (s), 3163 (w), 1728 (s), 1510 (m), 1405 (s), 1309 (m), 1271 (m), 1222 (s), 1155 (m), 1101 (s), 987 (m), 829 (w), 741 (m) cm^{-1}.
UV / Vis (DCM): λ_{max} = 282, 243 nm.
mp: 197 - 203 °C.

(5,6-Dimethoxyisoindolin-2-yl)(isoindolin-2-yl)methanone (5a)

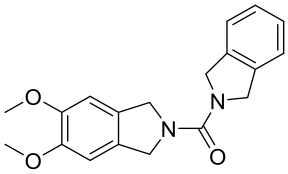

(5,6-Dimethoxyisoindolin-2-yl)(isoindolin-2-yl)methanone **5a** was prepared from **4a** (210 mg, 0.506 mmol, 1.0 eq.) according to **Method 3**. The crude product was purified by column chromatography (2 % MeOH in DCM) to yield **5a** as a colorless solid (160 mg, 98 %).

R_f = 0.41 (3 % MeOH in DCM, *v/v*).
^{1}**H-NMR** (400 MHz, $CDCl_3$): δ = 7.29 – 7.27 (m, 4H), 6.78 (s, 2H), 4.91 (s, 4H), 4.86 (s, 4H), 3.89 (s, 6H) ppm.
^{13}**C-NMR** (101 MHz, $CDCl_3$): δ = 160.5, 149.2, 137.2, 128.8, 127.5, 122.6, 105.5, 56.3, 54.5, 54.4 ppm.
HRMS (ESI, pos): *m/z* $[M+H]^+$ calculated for $[C_{19}H_{21}N_2O_3]^+$: 325.1547, found: 325.1543.
IR (KBr): $\tilde{\nu}$ = 3446 (m), 2867 (s), 1637 (s), 1507 (s), 1392 (s), 1347 (s), 1276 (s), 1221 (s), 1188 (s), 1102 (s), 992 (m), 841 (m), 752 (s) cm^{-1}.
UV / Vis (DCM): λ_{max} = 291, 242 nm.
mp: 197 - 199 °C.

(5,6-Dihydroxyisoindolin-2-yl)(isoindolin-2-yl)methanone (6a)

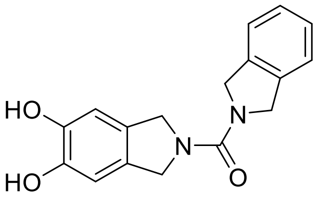

To a solution of **5a** (50 mg, 0.15 mmol, 1.0 eq.) in DCM (1.3 mL) was added BBr_3 (1 M solution in DCM, 0.90 mL, 0.90 mmol, 6.0 eq.) dropwise at 0 °C over 15 min. The solution was stirred for 1.5 h at 0 °C. Afterwards, the reaction mixture was quenched by the addition of methanol (1 mL) and the solvent was removed under reduced pressure. The crude product was used *in-situ* for the next reaction step without further purification.

$\mathbf{R_f}$ = 0.43 (10 % MeOH in DCM, *v/v*).
^{1}H-NMR (400 MHz, DMSO-d_6): δ = 7.35 – 7.26 (m, 4H), 6.67 (s, 2H), 4.81 (s, 4H), 4.65 (s, 4H) ppm.
HRMS (ESI, neg): *m/z* $[M-H]^-$ calculated for $[C_{17}H_{15}N_2O_3]^-$: 297.1234, found: 297.1244.

Tetrabenzyl (2-(isoindoline-2-carbonyl)isoindoline-5,6-diyl) bis(phosphate) (7a)

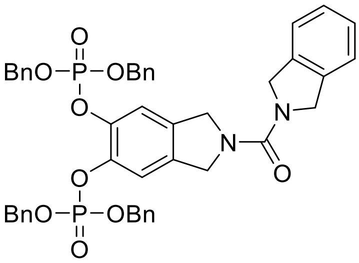

Tetrabenzyl (2-(isoindoline-2-carbonyl)isoindoline-5,6-diyl) bis(phosphate) **7a** was synthesized from **6a** (46 mg, 0.16 mmol, 1.0 eq.) according to **Method 5**. Purification of the crude product by column chromatography (3:2 EtOAc / hexane, *v/v*) yielded **7a** as a colorless oil (59 mg, 46 %).

$\mathbf{R_f}$ = 0.30 (3:2 EtOAc / hexane, *v/v*).
^{1}H-NMR (400 MHz, $CDCl_3$): δ = 7.33 – 7.23 (m, 24H), 7.18 (s, 2H), 5.10 (d, $^3J_{H-P}$ = 8.3 Hz, 8H, OC**H**$_2$Ph), 4.89 (s, 4H), 4.77 (s, 4H) ppm.
^{31}P-NMR (162 MHz, $CDCl_3$): δ = - 6.16 ppm.

^{13}**C-NMR** (101 MHz, $CDCl_3$): δ = 160.4, 141.1 (t, $^{2,3}J_{C\text{-}P}$ = 6.7 Hz), 137.0, 135.4 (d, $^{3}J_{C\text{-}P}$ = 7.1 Hz), 134.4, 128.8, 128.7, 128.2, 127.6, 122.6, 115.8, 70.4 (d, $^{2}J_{C\text{-}P}$ = 6.0 Hz, O**C**H_2Ph), 54.4, 54.0 ppm.
HRMS (ESI, pos): *m/z* $[M+H]^+$ calculated for $[C_{45}H_{43}N_2O_9P_2]^+$: 817.2438, found: 817.2449.
IR (KBr): $\tilde{\nu}$ = 3477 (br), 3033 (w), 1631 (s), 1504 (s), 1414 (m), 1352 (s), 1284 (s), 1215 (m), 1016 (s), 959 (s), 905 (s), 743 (s), 697 (s), 601 (w) cm^{-1}.
UV / Vis (DCM): λ_{max} = 273 nm.

2-(Isoindoline-2-carbonyl)isoindoline-5,6-diyl bis(dihydrogen phosphate) (1a)

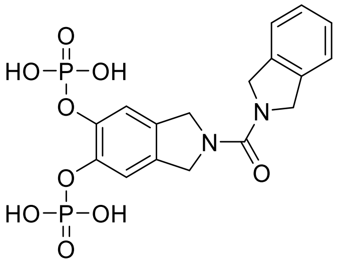

2-(Isoindoline-2-carbonyl)isoindoline-5,6-diyl bis(dihydrogen phosphate) **1a** was prepared from **7a** (35 mg, 0.043 mmol, 1.0 eq.) according to **Method 6**. Precipitation of the crude product from acetonitrile and subsequent filtration yielded **1a** as a colorless solid (10 mg, 50 %).

R$_f$ = 0.76 (RP-TLC, 1:1 H_2O / CH_3CN, *v/v*).
^{1}**H-NMR** (400 MHz, DMSO-d_6): δ = 7.35 – 7.28 (m, 4H), 7.27 (s, 2H), 4.82 (s, 4H), 4.77 (s, 4H) ppm.
^{31}**P-NMR** (162 MHz, D_2O): δ = - 6.31 ppm.
^{13}**C-NMR** (101 MHz, DMSO-d_6): δ = 159.4, 142.4 (t, $^{2,3}J_{C\text{-}P}$ = 6.1 Hz), 137.1, 133.1, 127.5, 122.7, 116.3, 53.9, 53.6 ppm.
HRMS (ESI, neg): *m/z* $[M-H]^-$ calculated for $[C_{17}H_{17}N_2O_9P_2]^-$: 455.0415, found: 455.0409.
IR (KBr): $\tilde{\nu}$ = 3436 (br), 1627 (w), 1503 (w), 1444 (w), 1353 (w), 1316 (w), 1174 (w), 1099 (w), 975 (w), 752 (w), 505 (w) cm^{-1}.
UV / Vis (H_2O): λ_{max} = 272 nm.
mp: 220 - 225 °C

(6,7-Dimethoxy-3,4-dihydroisoquinolin-2(1*H*)-yl)(1*H*-imidazol-1-yl)methanone (3b)

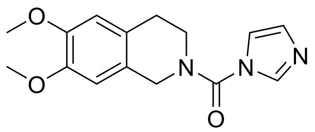

(6,7-Dimethoxy-3,4-dihydroisoquinolin-2(1*H*)-yl)(1*H*-imidazol-1-yl)methanone **3b** was synthesized from 6,7-dimethoxy-1,2,3,4-tetrahydroisoquinoline hydrochloride **2b** (1.00 g, 4.30 mmol, 1.0 eq.) according to **Method 1**. Additionally, triethylamine (595 µL, 4.30 mmol, 1.0 eq.) was added to the suspension. The crude product was purified by column chromatography (2 % MeOH in DCM) to yield **3b** as a colorless solid (955 mg, 80 %).

R_f = 0.28 (2 % MeOH in DCM, *v/v*).
^{1}H-NMR (400 MHz, $CDCl_3$): δ = 7.93 (t, *J* = 1.1 Hz, 1H), 7.27 (t, *J* = 1.4 Hz, 1H), 7.12 (dd, *J* = 1.5, 0.9 Hz, 1H), 6.65 (s, 1H), 6.56 (s, 1H), 4.67 (s, 2H), 3.87 (s, 3H), 3.84 (s, 3H), 3.81 (t, *J* = 5.9 Hz, 2H), 2.92 (t, *J* = 5.9 Hz, 2H) ppm.
^{13}C-NMR (101 MHz, $CDCl_3$): δ = 151.2, 148.4, 148.2, 136.9, 130.0, 125.6, 123.6, 118.0, 111.6, 109.0, 56.1, 48.4, 44.7, 28.2 ppm.
HRMS (ESI, pos): *m/z* $[M+H]^+$ calculated for $[C_{15}H_{17}N_3O_3]^+$: 288.1343, found: 288.1344.
IR (KBr): $\tilde{\nu}$ = 3433 (br), 3125 (s), 2838 (m), 1683 (s), 1612 (s), 1521 (s), 1434 (s), 1323 (m), 1292 (s), 1235 (s), 1115 (s), 1001 (s), 861 (s), 749 (s), 658 (s) cm^{-1}.
UV / Vis ($CHCl_3$): λ_{max} = 287, 242 nm.
mp: 139 - 141 °C.

1-(6,7-Dimethoxy-1,2,3,4-tetrahydroisoquinoline-2-carbonyl)-3-methyl-1*H*-imidazol-3-ium iodide (4b)

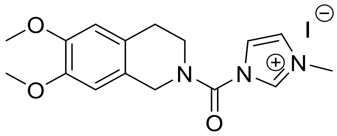

1-(6,7-Dimethoxy-1,2,3,4-tetrahydroisoquinoline-2-carbonyl)-3-methyl-1*H*-imidazol-3-ium iodide **4b** was synthesized from **3b** (600 mg, 2.09 mmol, 1.0 eq.) according to **Method 2**. The solvent was removed in vacuo to yield the carbamoylimidazolium salt **4b** as a yellow solid (884 mg, 99 %).

^{1}**H-NMR** (400 MHz, DMSO-d_6, recorded at 90°C): δ = 9.57 – 9.54 (m, 1H), 8.04 (t, *J* = 1.9 Hz, 1H), 7.87 – 7.81 (m, 1H), 6.83 (s, 1H), 6.82 (s, 1H), 4.65 (s, 2H), 3.97 (s, 3H), 3.77 (s, 3H), 3.75 (s, 3H), 3.72 (t, *J* = 6.3 Hz, 2H), 2.90 (t, *J* = 6.1 Hz, 2H) ppm.
^{13}**C-NMR** (101 MHz, DMSO-d_6, HSQC recorded at 60°C): δ = 147.8, 147.5, 147.0, 137.7, 125.7, 123.6, 123.3, 121.0, 111.8, 109.8, 55.6, 47.4, 44.4, 36.4, 27.5 ppm.
HRMS (ESI, pos): *m/z* $[M]^+$ calculated for $[C_{16}H_{20}N_3O_3]^+$: 302.1499, found: 302.1500.
IR (KBr): $\tilde{\nu}$ = 3438 (br), 3074 (w), 1724 (s), 1520 (s), 1437 (m), 1353 (w), 1256 (m), 1220 (s), 1140 (w), 1111 (s), 1001 (w), 869 (w), 744 (m) cm^{-1}.
UV / Vis ($CHCl_3$): λ_{max} = 283, 242 nm.
mp: 200 - 220 °C.

(6,7-Dimethoxy-3,4-dihydroisoquinolin-2(1*H*)-yl)(isoindolin-2-yl)methanone (5b)

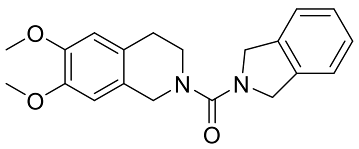

(6,7-Dimethoxy-3,4-dihydroisoquinolin-2(1*H*)-yl)(isoindolin-2-yl)methanone **5b** was synthesized from **4b** (300 mg, 0.700 mmol, 1.0 eq.) according to **Method 3**. The crude product was purified by column chromatography (1 % MeOH in DCM) to yield **5b** as a colorless solid (237 mg, quant.).

R_f = 0.20 (1 % MeOH in DCM, *v/v*).
^{1}**H-NMR** (300 MHz, acetone-d_6): δ = 7.34 – 7.25 (m, 4H), 6.76 (s, 1H), 6.73 (s, 1H), 4.81 (s, 4H), 4.43 (s, 2H), 3.78 (s, 3H), 3.77 (s, 3H), 3.56 (t, *J* = 5.8 Hz, 2H), 2.88 – 2.83 (m, 2H) ppm.
^{13}**C-NMR** (75 MHz, acetone-d_6): δ = 163.5, 149.2, 149.1, 138.6, 128.1, 127.7, 127.1, 123.4, 113.3, 111.0, 56.3, 54.7, 49.0, 45.2, 29.0 ppm.
HRMS (ESI, pos): *m/z* $[M+H]^+$ calculated for $[C_{20}H_{23}N_2O_3]^+$: 339.1703, found: 339.1715.
IR (KBr): $\tilde{\nu}$ = 3440 (s), 2931 (m), 1614 (s), 1518 (s), 1420 (s), 1310 (m), 1254 (s), 1228 (s), 1105 (s), 1012 (w), 847 (w), 753 (m) cm^{-1}.
UV / Vis (DCM): λ_{max} = 286, 230 nm.
mp: 160 - 163 °C.

(6,7-Dihydroxy-3,4-dihydroisoquinolin-2(1*H*)-yl)(isoindolin-2-yl)methanone 6b

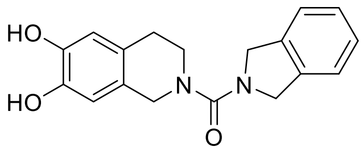

(6,7-Dihydroxy-3,4-dihydroisoquinolin-2(1*H*)-yl)(isoindolin-2-yl)methanone **6b** was prepared from **5b** (300 mg, 0.888 mmol, 1.0 eq.) following **Method 4**. The residue was extracted with EtOAc (3 x 15 mL) and washed with brine (1 x 15 mL). The combined organic layers were dried over Na_2SO_4, filtered, and concentrated in vacuo. Purification of the crude product by column chromatography (7:3 EtOAc / hexane, *v/v*) yielded **6b** as a colorless solid (162 mg, 59 %).

R_f = 0.31 (7:3 EtOAc / hexane, *v/v*).
^{1}H-NMR (400 MHz, DMSO-d_6): δ = 7.34 – 7.24 (m, 4H), 6.50 (s, 2H), 4.73 (s, 4H), 4.26 (s, 2H), 3.44 (t, *J* = 5.8 Hz, 2H), 2.72 – 2.65 (m, 2H) ppm.
^{13}C-NMR (101 MHz, DMSO-d_6): δ = 162.1, 143.9, 143.6, 137.2, 127.1, 124.8, 124.2, 122.4, 115.3, 113.1, 53.5, 47.5, 44.1, 27.5 ppm.
HRMS (ESI, neg): *m/z* $[M-H]^-$ calculated for $[C_{18}H_{17}N_2O_3]^-$: 309.1245, found: 309.1235.
IR (KBr): $\tilde{\nu}$ = 3470 (br), 3044 (m), 2862 (w), 1598 (s), 1577 (s), 1483 (s), 1453 (s), 1422 (s), 1354 (s), 1317 (s), 1279 (s), 1193 (m), 1093 (w), 932 (w), 761 (s) cm^{-1}.
UV / Vis (MeOH): λ_{max} = 288, 206 nm.
mp: 234 - 238 °C.

Tetrabenzyl (2-(isoindoline-2-carbonyl)-1,2,3,4-tetrahydroisoquinoline-6,7-diyl) bis(phosphate) (7b)

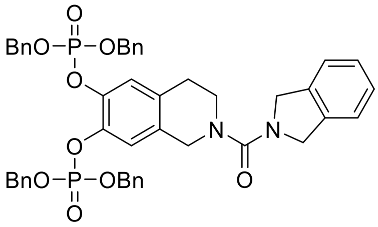

Tetrabenzyl (2-(isoindoline-2-carbonyl)-1,2,3,4-tetrahydroisoquinoline-6,7-diyl) bis(phosphate) **7b** was prepared from **6b** (10 mg, 0.032 mmol, 1.0 eq.) according to **Method 5**. Purification of the crude product by column chromatography (3:2 EtOAC / hexane, *v/v*) yielded **7b** as a colorless oil (13 mg, 48 %).

R_f = 0.23 (3:2 EtOAc / hexane, *v/v*).

^{1}H-NMR (400 MHz, $CDCl_3$): δ = 7.31 – 7.23 (m, 24H), 7.08 (s, 1H), 6.99 (s, 1H), 5.10 (d, $^3J_{P-H}$ = 8.2 Hz, 4H, OC**H**$_2$Ph), 5.08 (d, $^3J_{P-H}$ = 8.2 Hz, 4H, OC**H**$_2$Ph), 4.82 (s, 4H), 4.36 (s, 2H), 3.53 (t, *J* = 5.7 Hz, 2H), 2.85 – 2.77 (m, 2H) ppm.

^{31}P-NMR (162 MHz, $CDCl_3$) δ = - 6.19, - 6.16 ppm.

^{13}C-NMR (101 MHz, $CDCl_3$): δ = 162.9, 139.7 (t, $^{2,3}J_{C-P}$ = 6.8 Hz), 139.6 (t, $^{2,3}J_{C-P}$ = 6.8 Hz), 137.2, 135.50 (d, $^3J_{C-P}$ = 6.9 Hz), 135.43 (d, $^3J_{C-P}$ = 6.8 Hz), 132.6, 131.5, 128.71 (d, $^4J_{C-P}$ = 1.2 Hz), 128.66, 128.1, 127.5, 122.5, 121.69 (d, $^3J_{C-P}$ = 2.6 Hz), 119.25 (d, $^3J_{C-P}$ = 2.6 Hz), 70.2 (d, $^2J_{C-P}$ = 5.7 Hz, O**C**H$_2$Ph), 54.2, 48.1, 44.0, 28.3 ppm.

HRMS (ESI, pos): *m/z* $[M+H]^+$ calculated for $[C_{46}H_{45}N_2O_9P_2]^+$: 831.2598, found: 831.2595.

IR (KBr): ṽ = 3477 (w), 3033 (w), 1638 (m), 1513 (w), 1456 (w), 1415 (m), 1282 (m), 1214 (w), 1016 (s), 961 (m), 898 (w), 744 (m), 697 (m) cm^{-1}.

UV / Vis (DCM): λ_{max} = 272, 228 nm.

2-(Isoindoline-2-carbonyl)-1,2,3,4-tetrahydroisoquinoline-6,7-diyl bis (dihydrogen phosphate) (1b)

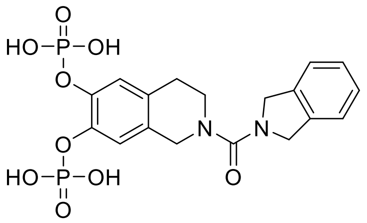

2-(Isoindoline-2-carbonyl)-1,2,3,4-tetrahydroisoquinoline-6,7-diyl bis (dihydrogen phosphate) **1b** was synthesized from **7b** (60 mg, 0.072 mmol, 1.0 eq.) according to **Method 6**. The crude product was purified by reversed-phase column chromatography (100 % H_2O). Lyophilization yielded **1b** as a colorless solid (22 mg, 66 %).

R_f = 0.8 (RP-TLC, 1:1 H_2O / CH_3CN, *v/v*).

^{1}H-NMR (400 MHz, D_2O): δ = 7.42 – 7.34 (m, 4H), 7.21 (s, 1H), 7.16 (s, 1H), 4.86 (s, 4H), 4.53 (s, 2H), 3.64 (t, *J* = 5.8 Hz, 2H), 2.94 (t, *J* = 5.8 Hz, 2H) ppm.

^{31}P-NMR (162 MHz, D_2O) δ = -1.75 ppm.

^{13}C-NMR (101 MHz, D_2O): δ = 163.8, 142.5 – 142.3 (m), 142.2 – 142.0 (m), 136.7, 130.4, 128.9, 127.4, 122.4, 121.7, 119.5, 53.7, 47.6, 44.2, 27.6 ppm.

HRMS (ESI, neg): *m/z* $[M-H]^-$ calculated for $[C_{18}H_{19}N_2O_9P_2]^-$: 469.0571, found: 469.0557.

IR (KBr): ṽ = 3434 (br), 3223 (s), 2858 (w), 1628 (s), 1512 (s), 1418 (s), 1307 (m), 1281 (m), 1195 (m), 1091 (s), 982 (w), 925 (s), 837 (w), 743 (m) cm^{-1}.

UV / Vis (H_2O): λ_{max} = 272 nm.

***Tert*-butyl-(3-(3,4-dimethoxyphenyl)propyl)carbamate (X1)**

Tert-butyl-(3-(3,4-dimethoxyphenyl)propyl)carbamate **X1** was prepared essentially as described by *In et al*.[15]

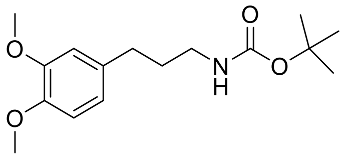

In brief, NEt_3 (4.0 mL, 29 mmol, 5.8 eq.) and Boc_2O (2.0 mL, 8.7 mmol, 1.7 eq.) were added to a solution of 3-(3,4-dimethoxyphenyl)-propane-1-amine (1.0 g, 5.1 mmol, 1.0 eq.) in dry DCM (25 mL) at 0 °C. After stirring at rt overnight, the reaction mixture was quenched by the addition of brine (25 mL) at 0 °C. The mixture was then extracted with DCM (2 x 30 mL). The combined organic layers were washed with a saturated NH_4Cl solution (1 x 25 mL), dried over Na_2SO_4 and concentrated in vacuo. The residue was purified by column chromatography (3:1 hexane / EtOAc, *v/v*) to give **X1** as a colorless solid (1.49 g, 99 %).

$\mathbf{R_f}$ = 0.25 (3:1 hexane / EtOAc, *v/v*).
^{1}H-NMR (400 MHz, $CDCl_3$): δ = 6.81 – 6.77 (m, 1H), 6.74 – 6.69 (m, 2H), 4.52 (s, 1H, N**H**), 3.87 (s, 3H), 3.85 (s, 3H), 3.22 – 3.09 (m, 2H), 2.64 – 2.54 (m, 2H), 1.85 – 1.74 (m, 2H), 1.44 (s, 9H) ppm.
^{13}C-NMR (101 MHz, $CDCl_3$): δ = 156.1, 149.0, 147.4, 134.4, 120.3, 111.9, 111.5, 79.3, 56.1, 56.0, 40.4, 32.9, 32.1, 28.6 ppm.
HRMS (ESI, pos): *m/z* $[M+Na]^+$ calculated for $[C_{16}H_{25}NO_4Na]^+$: 318,1676, found: 318,1683.
IR (KBr): $\tilde{\nu}$ = 3348 (br), 2969 (s), 2926 (s), 2855 (s), 1704 (s), 1683 (s), 1518 (s), 1452 (s), 1366 (s), 1291 (s), 1250 (s), 1168 (s), 1134 (s), 1079 (m), 1030 (s), 1000 (s), 957 (m), 870 (s), 809 (s), 767 (s) cm^{-1}.
UV / Vis (DCM): λ_{max} = 281, 240 nm.
mp: 72 - 74 °C (lit.: 69.5 - 72.0 °C).[15]

7,8-Dimethoxy-2,3,4,5-tetrahydro-1*H*-benzo[*c*]azepine-1-one (X2)

7,8-Dimethoxy-2,3,4,5-tetrahydro-1*H*-benzo[*c*]azepine-1-one **X2** was prepared essentially as described by *In et al*.[15]

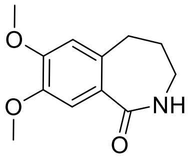

In brief, 2-chloropyridine (141 µL, 1.50 mmol, 1.5 eq.) and Tf_2O (185 µL, 1.10 mmol, 1.1 eq.) were added to a stirred solution of *N*-Boc-carbamate **X1** (300 mg, 1.00 mmol, 1.0 eq.) in DCM (20 mL) at -78 °C. After 20 min, TfOH (439 µL, 5.00 mmol, 5.0 eq.) was added at -78 °C. After another 10 min, the reaction mixture was warmed to rt and stirred for 2 h. Next, the reaction was quenched by the addition of $NaHCO_3$ (20 mL) at 0 °C, followed by the extraction with DCM (3 x 30 mL). The combined organic layers were dried over Na_2SO_4, filtered and concentrated in vacuo. The residue was purified by column chromatography (3 % MeOH in DCM, *v/v*) to yield the cyclized product **X2** as a colorless solid (152 mg, 68 %).

R_f = 0.28 (3 % MeOH in DCM, *v/v*).
^{1}H-NMR (400 MHz, $CDCl_3$): δ = 7.27 (s, 1H), 6.68 (s, 1H), 6.55 (s, 1H, N**H**), 3.92 (s, 3H), 3.91 (s, 3H), 3.14 (q, *J* = 6.5 Hz, 2H), 2.82 (t, *J* = 7.1 Hz, 2H), 2.01 (p, *J* = 6.8 Hz, 2H) ppm.
^{13}C-NMR (101 MHz, $CDCl_3$): δ = 174.0, 151.0, 147.7, 132.1, 126.7, 111.8, 111.5, 56.04, 55.96, 39.9, 30.8, 30.2 ppm.
HRMS (ESI, pos): *m/z* $[M+H]^+$ calculated for $[C_{12}H_{16}NO_3]^+$: 222.1125, found: 222.1116.
IR (KBr): $\tilde{\nu}$ = 3430 (br), 3179 (s), 2951 (s), 2862 (s), 1647 (s), 1604 (s), 1513 (s), 1465 (s), 1365 (s), 1263 (s), 1217 (s), 1110 (s), 1084 (s), 1016 (s), 951 (m), 878 (s), 817 (s), 795 (s) cm^{-1}.
UV / Vis (DCM): λ_{max} = 292, 259 nm.
mp: 182 - 184 °C (lit.: 183.0 - 184.2 °C).[15]

7,8-Dimethoxy-2,3,4,5-tetrahydro-1*H*-benzo[*c*]azepine (2c)

7,8-Dimethoxy-2,3,4,5-tetrahydro-1*H*-benzo[*c*]azepine **2c** was prepared essentially as described by *Tafesse und Kyle*.[16]

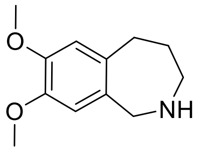

In brief, to a solution of $LiAlH_4 \cdot$ THF (0.72 mL, 0.72 mmol, 2.0 eq.) in THF (1.2 mL) was added a solution of tetrahydro-1*H*-benzo[c]azepinone **X2** (80 mg, 0.36 mmol, 1.0 eq.) in THF (2 mL) dropwise at 0 °C. The resulting mixture was stirred for 20 min at 0 °C and then heated to 60 °C for 8 h. After successive addition of 0.5 mL water, 0.5 mL 10 % NaOH and again 0.5 mL water at 0 °C, the mixture was stirred for 30 min. The resulting suspension was filtered and the residue was extracted with EtOAc (3 x 5 mL). The combined organic layers were dried over Na_2SO_4, filtered and concentrated in vacuo. The resulting oil was used for the next reaction step without further purification.

^{1}**H-NMR** (400 MHz, $CDCl_3$): δ = 6.69 (s, 1H), 6.67 (s, 1H), 3.87 (s, 2H), 3.85 (s, 3H), 3.84 (s, 3H), 3.22 –3.16 (m, 2H), 2.90 – 2.84 (m, 2H), 1.77 – 1.66 (m, 2H) ppm.
^{13}**C-NMR** (101 MHz, $CDCl_3$): δ = 147.4, 146.6, 135.3, 134.9, 113.5, 112.7, 56.2, 54.8, 53.6, 35.9, 31.1 ppm.
HRMS (ESI, pos): *m/z* $[M+H]^+$ calculated for $[C_{12}H_{18}NO_2]^+$: 208.1332, found: 208.1323.

(7,8-Dimethoxy-1,3,4,5-tetrahydro-2*H*-benzo[*c*]azepine-2-yl)-(1*H*-imidazol-1-yl)-methanone (3c)

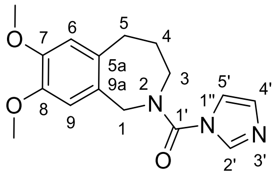

(7,8-Dimethoxy-1,3,4,5-tetrahydro-2*H*-benzo[*c*]azepine-2-yl)-(1*H*-imidazol-1-yl)-methanone **3c** was prepared from **2c** (66 mg, 0.32 mmol, 1.0 eq.) following **Method 1**. The crude product was purified by column chromatography (0 - 3 % MeOH in DCM) to yield **3c** as a yellow oil (50 mg, 46 % over two steps).

$\mathbf{R_f}$ = 0.23 (3 % MeOH in DCM, *v/v*).
^{1}**H-NMR** (300 MHz, DMSO-d_6, recorded at 90°C): δ = 7.85 (t, *J* = 1.1 Hz, 1H, **H-2“**), 7.35 (t, *J* = 1.4 Hz, 1H, **H-5“**), 7.03 (dd, *J* = 1.5, 0.9 Hz, 1H, **H-4“**), 6.82 (s, 1H, **H-6**), 6.50 (s, 1H, **H-9**), 4.55 (s, 2H, **H-1**), 3.76 (s, 3H, -OC**H**$_3$), 3.75 – 3.70 (m, 2H, **H-3**), 3.68 (s, 3H, -OC**H**$_3$), 2.95 – 2.89 (m, 2H, **H-5**), 1.88 – 1.79 (m, 2H, **H-4**) ppm.
^{13}**C-NMR** (75 MHz, DMSO-d_6, recorded at 90°C): δ = 151.6 (**C-1‘**), 147.9 (**C-7,8**), 146.5 (**C-7,8**), 136.2 (**C-2“**), 133.8 (**C-5a**), 129.1 (**C-9a**), 128.3 (**C-4“**), 117.4 (**C-5“**), 114.5 (**C-6**), 113.9 (**C-9**), 55.7 (-O**C**H$_3$), 55.6 (-O**C**H$_3$), 52.2 (**C-1**), 51.0 (**C-3**), 32.6 (**C-5**), 27.1 (**C-4**) ppm.
HRMS (ESI, pos): *m/z* $[M+H]^+$ calculated for $[C_{16}H_{20}N_3O_3]^+$: 302.1499, found: 302.1507.
IR (KBr): $\tilde{\nu}$ = 3436 (br), 2924 (s), 2836 (m), 1705 (s), 1606 (s), 1519 (s), 1427 (s), 1337 (s), 1264 (s), 1229 (s), 1111 (s), 999 (s), 861 (s), 771 (s), 655 (s) cm^{-1}.
UV / Vis ($CHCl_3$): λ_{max} = 318, 285, 242 nm.

1-(7,8-Dimethoxy-2,3,4,5-tetrahydro-1*H*-benzo[*c*]azepine-2-carbonyl)-3-methyl-1*H*-imidazol-3-ium iodide (4c)

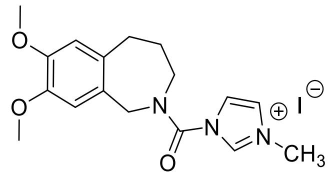

1-(7,8-Dimethoxy-2,3,4,5-tetrahydro-1*H*-benzo[*c*]azepine-2-carbonyl)-3-methyl-1*H*-imidazol-3-ium **4c** was prepared from **3c** (358 mg, 1.20 mmol, 1.0 eq.) following **Method 2**. The solvent was removed in vacuo to yield the carbamoylimidazolium salt as a light-yellow solid (483 mg, 92 %).

^{1}H-NMR (400 MHz, DMSO-d_6, recorded at 60 °C): δ = 9.54 – 9.49 (m, 1H), 7.95 (t, *J* = 1.8 Hz, 1H), 7.84 (t, *J* = 1.8 Hz, 1H), 6.86 (s, 1H), 6.81 – 6.62 (m, 1H), 4.60 (s, 2H), 3.92 (s, 3H), 3.75 (s, 3H), 3.75 – 3.71 (m, 2H), 3.70 (s, 3H), 3.03 – 2.90 (m, 2H), 1.90 – 1.79 (m, 2H) ppm.
^{13}C-NMR (75 MHz, DMSO-d_6, HSQC recorded at 60°C): δ = 148.2, 146.9, 146.6, 136.9, 134.0, 127.7, 123.6, 120.7, 114.4, 114.2, 55.9, 55.7, 52.6, 51.7, 36.2, 32.5, 27.3 ppm.
HRMS (ESI, pos): *m/z* [M]$^+$ calculated for $[C_{17}H_{22}N_3O_3]^+$: 316,1656, found: 316,1654.
IR (KBr): ṽ = 3435 (s), 2935 (w), 1723 (s), 1520 (m), 1428 (m), 1339 (w), 1272 (w), 1226 (w), 1111 (m), 997 (w), 851 (w), 737 (w) cm^{-1}.
UV / Vis ($CHCl_3$): λ_{max} = 274, 242 nm.
mp: 191 - 200 °C.

(7,8-Dimethoxy-1,3,4,5-tetrahydro-2*H*-benzo[*c*]azepin-2-yl)(isoindolin-2-yl) methanone (5c)

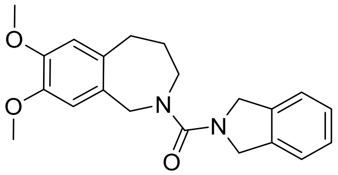

(7,8-Dimethoxy-1,3,4,5-tetrahydro-2*H*-benzo[*c*]azepin-2-yl)(isoindolin-2-yl) methanone **5c** was prepared from **4c** (330 mg, 0.745 mmol, 1.0 eq.) following **Method 3**. The crude product was purified by column chromatography (2 % MeOH in DCM) to yield **5c** as a colorless solid (265 mg, quant.).

R$_f$ = 0.30 (2 % MeOH in DCM, *v/v*).

^{1}H-NMR (300 MHz, $CDCl_3$): δ = 7.25 – 7.18 (m, 4H), 6.77 (s, 1H), 6.69 (s, 1H), 4.76 (s, 4H), 4.47 (s, 2H), 3.87 (s, 3H), 3.84 (s, 3H), 3.68 – 3.62 (m, 2H), 2.97 – 2.89 (m, 2H), 2.01 – 1.91 (m, 2H) ppm.
^{13}C-NMR (101 MHz, $CDCl_3$): δ = 163.1, 147.6, 146.7, 137.3, 133.9, 130.8, 127.2, 122.3, 113.3, 112.9, 56.1, 56.0, 54.6, 53.2, 52.4, 34.3, 28.4 ppm.
HRMS (ESI, pos): *m/z* $[M+H]^+$ calculated for $[C_{21}H_{25}N_2O_3]^+$: 353.1860, found: 353.1857.
IR (KBr): $\tilde{\nu}$ = 3445 (w), 2932 (w), 1628 (m), 1519 (m), 1416 (w), 1282 (w), 1254 (w), 1219 (w), 1112 (w), 844 (w), 743 (w) cm^{-1}.
UV / Vis ($CHCl_3$): λ_{max} = 286, 241 nm.
mp: 163 - 165 °C.

(7,8-Dihydroxy-1,3,4,5-tetrahydro-2*H*-benzo[*c*]azepin-2-yl)(isoindolin-2-yl) methanone (6c)

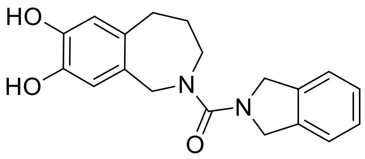

(7,8-Dihydroxy-1,3,4,5-tetrahydro-2*H*-benzo[*c*]azepin-2-yl)(isoindolin-2-yl) methanone **6c** was prepared from **5c** (40 mg, 0.11 mmol, 1.0 eq.) following **Method 4**. The residue was taken up in brine (1 x 10 mL) and extracted with DCM (20 mL). The organic layer was dried over Na_2SO_4, filtered and concentrated in vacuo. Purification of the crude product by column chromatography (7:3 EtOAc / hexane, *v/v*) yielded **6c** as a colorless solid (16 mg, 43 %).

R_f = 0.38 (7:3 EtOAc / hexane, *v/v*).
^{1}H-NMR (400 MHz, DMSO-d_6): δ = 8.63 (s, 1H, O**H**), 8.58 (s, 1H, O**H**), 7.31 – 7.23 (m, 4H), 6.60 (s, 1H), 6.55 (s, 1H), 4.68 (s, 4H), 4.31 (s, 2H), 3.52 (t, *J* = 5.3 Hz, 2H), 2.79 – 2.72 (m, 2H), 1.81 – 1.74 (m, 2H) ppm.
^{13}C-NMR (101 MHz, DMSO-d_6): δ = 161.6, 143.6, 142.3, 137.3, 132.6, 129.3, 127.1, 122.4, 117.0, 116.8, 53.9, 52.1, 52.0, 33.5, 28.2 ppm.
HRMS (ESI, pos): *m/z* $[M+H]^+$ calculated for $[C_{19}H_{21}N_2O_3]^+$: 325.1547, found: 325.1557.

Tetrabenzyl (2-(isoindoline-2-carbonyl)-2,3,4,5-tetrahydro-1*H*-benzo[*c*]azepine-7,8-diyl) bis(phosphate) (7c)

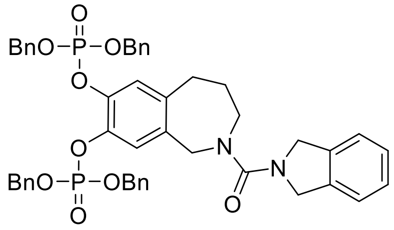

Tetrabenzyl (2-(isoindoline-2-carbonyl)-2,3,4,5-tetrahydro-1*H*-benzo[*c*]azepine-7,8-diyl) bis(phosphate) **7c** was prepared from **6c** (16 mg, 0.050 mmol, 1.0 eq.) in dry DMF (600 µL) and dry CH_3CN (200 µL) according to **Method 5**. Purification of the crude product by column chromatography (3:2 EtOAC / hexane, *v/v*) yielded **7c** as a colorless oil (28 mg, 60 %).

R_f = 0.28 (3:2 EtOAC / hexane, *v/v*).
^{1}H-NMR (300 MHz, $CDCl_3$): δ = 7.31 – 7.18 (m, 24H), 7.15 (s, 1H), 7.12 (s, 1H), 5.10 (d, $^3J_{P-H}$ = 8.2 Hz, 4H, OC**H**$_2$Ph), 5.07 (d, $^3J_{P-H}$ = 8.4 Hz, 4H, OC**H**$_2$Ph), 4.71 (s, 4H), 4.36 (s, 2H), 3.61 (t, *J* = 5.6 Hz, 2H), 2.89 – 2.80 (m, 2H), 1.97 – 1.86 (m, 2H) ppm.
^{31}P-NMR (162 MHz, $CDCl_3$) δ = - 6.06, - 6.50 ppm.
^{13}C-NMR (101 MHz, $CDCl_3$): δ = 163.1, 140.2 – 140.0 (m), 139.8, 139.0 – 138.8 (m), 137.3, 136.2, 135.6 (d, $^3J_{C-P}$ = 7.2 Hz), 128.73, 128.69, 128.66, 128.2 (d, $^4J_{C-P}$ = 3.5 Hz), 127.4, 122.7 – 122.6 (m), 122.5, 122.3 (d, $^3J_{C-P}$ = 2.5 Hz), 70.3 (d, $^2J_{C-P}$ = 5.5 Hz, O**C**H$_2$Ph), 70.2 (d, $^2J_{C-P}$ = 5.5 Hz, O**C**H$_2$Ph), 54.7, 53.1, 52.1, 34.1, 27.8 ppm.
HRMS (ESI, pos): *m/z* $[M+H]^+$ calculated for $[C_{47}H_{47}N_2O_9P_2]^+$: 845.2751, found: 845.2717.
IR (KBr): $\tilde{\nu}$ = 3477(w), 3033 (w), 1633 (m), 1509 (m), 1456 (m), 1414 (m), 1288 (s), 1215 (w), 1020 (s), 958 (s), 900 (m), 743 (s), 697 (s), 602 (w) cm^{-1}.
UV / Vis ($CHCl_3$): λ_{max} = 266, 240 nm.

2-(Isoindoline-2-carbonyl)-2,3,4,5-tetrahydro-1*H*-benzo[*c*]azepine-7,8-diyl bis(dihydrogen phosphate) (1c)

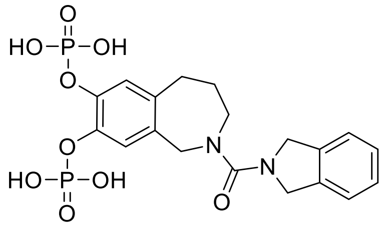

2-(Isoindoline-2-carbonyl)-2,3,4,5-tetrahydro-1*H*-benzo[*c*]azepine-7,8-diyl bis(dihydrogen phosphate) **1c** was prepared from **7c** (33 mg, 0.040 mmol, 1.0 eq.) according to **Method 6**.

The crude product was purified by reversed-phase column chromatography (100 % H_2O). Lyophilization yielded **1c** as a colorless solid (12 mg, 63 %).

R_f = 0.66 (RP-TLC, 3:2 H_2O / CH_3CN, *v/v*).
^{1}H-NMR (400 MHz, D_2O): δ = 7.36 – 7.34 (m, 4H), 7.23 (s, 1H), 7.22 (s, 1H), 4.79 (s, 4H), 4.53 (s, 2H), 3.71 (t, *J* = 5.6 Hz, 2H), 3.01 – 2.94 (m, 2H), 2.00 – 1.94 (m, 2H) ppm.
^{31}P-NMR (162 MHz, D_2O): δ = -2.11 ppm.
^{13}C-NMR (101 MHz, D_2O): δ = 163.8, 142.7 – 142.5 (m), 141.5 – 141.3 (m), 138.1, 136.9, 134.2, 127.4, 123.2, 122.5, 54.3, 52.2, 52.0, 33.2, 27.6 ppm.
HRMS (ESI, neg): *m/z* $[M-H]^-$ calculated for $[C_{19}H_{21}N_2O_9P_2]^-$: 483.0728, found: 483.0740.
IR (KBr): ṽ = 3435 (br), 1629 (m), 1512 (w), 1419 (m), 1303 (w), 1191 (m), 1104 (m), 924 (w), 745 (w), 619 (w) cm^{-1}.
UV / Vis (H_2O): λ_{max} = 270, 201 nm.
mp: 212 - 213 °C (decomposition).

(3,4-Dihydroisoquinolin-2(1H)-yl)(6,7-dimethoxy-3,4-dihydroisoquinolin-2(1H)-yl)methanone (5d)

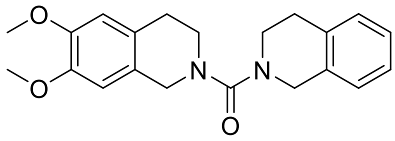

(3,4-Dihydroisoquinolin-2(1H)-yl)(6,7-dimethoxy-3,4-dihydroisoquinolin-2(1H)-yl)methanone **5d** was prepared from **4b** (600 mg, 1.40 mmol, 1.0 eq.) according to **Method 3**. The crude product was purified by column chromatography (1 % MeOH in DCM, *v/v*) to yield **5d** as a yellow oil (482 mg, 98 %).

R_f = 0.24 (1 % MeOH in DCM, *v/v*).
^{1}H-NMR (400 MHz, $CDCl_3$): δ = 7.20 – 7.07 (m, 4H), 6.63 (s, 1H), 6.58 (s, 1H), 4.49 (s, 2H), 4.41 (s, 2H), 3.86 (s, 3H), 3.85 (s, 3H), 3.56 (t, *J* = 6.3 Hz, 2H), 3.53 (t, *J* = 6.3 Hz, 2H), 2.95 (t, *J* = 5.8 Hz, 2H), 2.86 (t, *J* = 5.8 Hz, 2H) ppm.
^{13}C-NMR (100 MHz, $CDCl_3$): δ = 164.3, 147.9, 147.8, 134.8, 134.0, 129.0, 126.62, 126.57, 126.45, 126.3, 125.8, 111.8, 109.2, 56.13, 56.11, 49.0, 48.6, 44.9, 44.8, 28.9, 28.3 ppm.
HRMS (ESI, pos): *m/z* $[M+H]^+$ calculated for $[C_{21}H_{25}N_2O_3]^+$: 353.1860, found: 353.1864.
IR (KBr): ṽ = 3465 (br), 3000 (m), 2931 (m), 2834 (s), 1636 (br), 1518 (s), 1455 (s), 1420 (s), 1367 (s), 1226 (s), 1133 (m), 1100 (s), 933 (m), 750 (s) cm^{-1}.
UV / Vis (DCM): λ_{max} = 292, 245 nm.

(3,4-Dihydroisoquinolin-2(1H)-yl)(6,7-dihydroxy-3,4- dihydroisoquinolin-2(1H)-yl)methanone (6d)

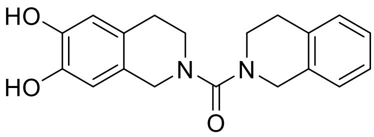

(3,4-Dihydroisoquinolin-2(1H)-yl)(6,7-dihydroxy-3,4-dihydroisoquinolin-2(1H)-yl)methanone **6d** was prepared from **5d** (50 mg, 0.14 mmol, 1.0 eq.) following **Method 4**. The residue was taken up in EtOAc (15 mL) and washed with brine (2 x 15 mL). The aqueous phase was reextracted with EtOAc (3 x 15 mL). The combined organic phases were dried over Na_2SO_4, filtered and the solvent was removed under reduced pressure. Purification of the crude product by column chromatography (7:3 EtOAc / hexane, *v*/*v*) yielded **6d** as a yellow solid (32 mg, 70 %).

R_f = 0.29 (7:3 EtOAc / hexane, *v/v*).
^{1}H-NMR (400 MHz, CD_3OD): δ = 7.18 – 7.09 (m, 4H), 6.55 (s, 1H), 6.51 (s, 1H), 4.57 (s, 2H), 4.47 (s, 2H), 4.33 (s, 2H), 3.56 (t, *J* = 5.9 Hz, 2H), 3.51 (t, *J* = 5.9 Hz, 2H), 2.93 (t, *J* = 5.8 Hz, 2H), 2.77 (t, *J* = 5.8 Hz, 2H) ppm.
^{13}C-NMR (75.5 MHz, CD_3OD): δ = 165.9, 145.2, 145.0, 135.8, 134.9, 129.8, 127.6, 127.3, 126.7, 125.7, 116.2, 113.7, 49.9, 49.5, 46.0, 45.9, 29.5, 28.9 ppm.
HRMS (ESI, pos): *m/z* $[M+H]^+$ calculated for $[C_{19}H_{21}N_2O_3]^+$ = 325.1547, found 325.1554.
IR (KBr): ṽ = 3436 (br), 3023 (w), 2924 (m), 2839 (m), 1604 (s), 1527 (m), 1486 (m), 1434 (s), 1369 (m), 1274 (m), 1242 (m), 933 (m), 751 (m) cm^{-1}.
UV / Vis (MeOH): λ_{max} = 291, 239 nm.

Tetrabenzyl (2-(1,2,3,4-tetrahydroisoquinoline-2-carbonyl)- 1,2,3,4-tetrahydroisoquinoline-6,7-diyl) bis(phosphate) (7d)

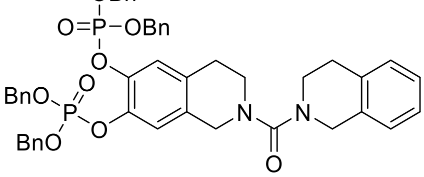

Tetrabenzyl (2-(1,2,3,4-tetrahydroisoquinoline-2-carbonyl)- 1,2,3,4-tetrahydroisoquinoline-6,7-diyl) bis(phosphate) **7d** was prepared from **6d** (25.0 mg, 0.077 mmol, 1.0 eq.) following **Method 5**. Purification of the crude product by column chromatography (3:2 EtOAc / hexane, *v/v*) afforded **7d** as a colorless oil (38 mg, 58 %).

R$_f$ = 0.26 (3:2 EtOAc / hexane, *v/v*).

^{1}H-NMR (400 MHz, $CDCl_3$): δ = 7.31 – 7.22 (m, 20H), 7.21 – 7.08 (m, 4H), 7.05 (s, 1H), 6.97 (s, 1H), 5.09 (d, $^3J_{P-H}$ = 8.2 Hz, 4H, OC**H**$_2$Ph), 5.08 (d, $^3J_{P-H}$ = 8.2 Hz, 4H, OC**H**$_2$Ph), 4.47 (s, 2H), 4.31 (s, 2H), 3.54 (t, J = 5.8 Hz, 2H), 3.47 (t, J = 5.7 Hz, 2H), 2.95 (t, J = 5.7 Hz, 2H), 2.78 (t, J = 5.6 Hz, 2H) ppm.

^{31}P-NMR (162 MHz, $CDCl_3$): δ = -6.20 ppm.

^{13}C-NMR (100 MHz, $CDCl_3$): δ = 164.0 (s), 139.9 – 139.5 (m), 135.6 (d, $^3J_{P-C}$ = 7.1 Hz), 135.5 (d, $^3J_{P-C}$ = 7.2 Hz), 134.7 (s), 133.8 (s), 132.5 (s), 131.4 (s), 129.1 (s), 128.7 (d, $^4J_{C-P}$ = 1.5 Hz), 128.69 (s), 128.2 (s), 126.7 (s), 126.44 (s), 126.35 (s), 121.8 (d, $^3J_{C-P}$ = 1.5 Hz), 119.2 (d, $^3J_{C-P}$ = 1.7 Hz), 70.3 (d, $^2J_{C-P}$ = 6.1 Hz, O**C**H$_2$Ph), 49.0, 48.4, 44.8, 44.2, 28.8, 28.1 ppm.

HRMS (ESI, pos): *m/z* $[M+H]^+$ calculated for $[C_{47}H_{47}N_2O_9P_2]^+$: 845.2751, found 845.2772.

UV/Vis (DCM): λ_{max} = 274 nm.

2-(1,2,3,4-Tetrahydroisoquinoline-2-carbonyl)-1,2,3,4- tetrahydroisoquinoline-6,7-diyl bis(dihydrogen phosphate) (1d)

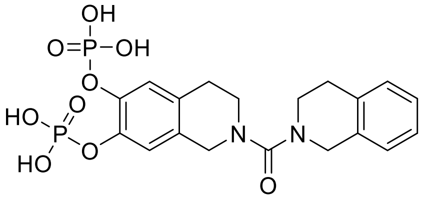

2-(1,2,3,4-Tetrahydroisoquinoline-2-carbonyl)-1,2,3,4-tetrahydroisoquinoline-6,7-diyl bis(dihydrogen phosphate) **1d** was prepared from **7d** (33 mg, 0.039 mmol, 1.0 eq.) according to **Method 6**. The crude product was washed with pentane (3 x) and purified by reversed-phase column chromatography (100 % H_2O). Lyophilization yielded **1d** as a colorless solid (17 mg, 88 %).

R$_f$ = 0.70 (RP-TLC, 7:3 H_2O / CH_3CN, *v/v*).

^{1}H-NMR (400 MHz, D_2O): δ = 7.31 – 7.21 (m, 4H), 7.18 (s, 1H), 7.14 (s, 1H), 4.54 (s, 2H), 4.50 (s, 2H), 3.64 – 3.56 (m, 4H), 3.00 – 2.94 (m, 2H), 2.94 – 2.89 (m, 2H) ppm.

^{31}P-NMR (162 MHz, D_2O): δ = -3.03 ppm.

^{13}C-NMR (75.5 MHz, D_2O): δ = 165.1, 142.0, 141.6, 134.8, 133.6, 130.7, 129.3, 128.8, 127.9, 126.8, 126.3, 121.7, 119.3, 48.4, 48.0, 44.5, 44.3, 27.9, 27.4 ppm.

HRMS (ESI, neg): *m/z* $[M-H]^-$ calculated for $[C_{19}H_{21}N_2O_9P_2]^-$: 483.0728, found 483.0716.

IR (KBr): ṽ = 3426 (br), 3173 (br), 1644 (br), 1513 (m), 1400 (s), 1308 (m), 1276 (m), 1191 (m), 1092 (m), 984 (m), 930 (m), 755 (m) cm^{-1}.

UV/Vis (H_2O): λ_{max} = 279, 236 nm.

1,2,3,4-Tetrahydroisoquinoline-6,7-diol hydrobromide (9b)

1,2,3,4-Tetrahydroisoquinoline-6,7-diol hydrobromide **9b** was prepared essentially as described by *Zheng et al*.[17]

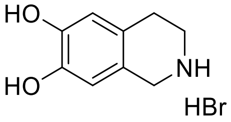

In brief, 6,7-dimethoxy-1,2,3,4-tetrahydroisoquinoline hydrochloride **2b** (100 mg, 0.437 mmol, 1.0 eq.) was dissolved in HBr (48 % in H_2O, 2 mL). The reaction mixture was heated to 105 °C for 5 h and upon completion of the reaction concentrated in vacuo. The residue was suspended in EtOAc and again concentrated in vacuo to yield **9b** without further purification as a colorless solid (105 mg, 98 %).

^{1}H-NMR (400 MHz, CD_3OD): δ = 6.62 (s, 1H), 6.58 (s, 1H), 4.18 (s, 2H), 3.43 (t, $^3J_{H\text{-}H}$ = 6.4 Hz, 2H), 2.95 (t, $^3J_{H\text{-}H}$ = 6.3 Hz, 2H) ppm.
^{13}C-NMR (101 MHz, CD_3OD): δ = 146.8, 146.0, 123.5, 119.7, 116.2, 114.0, 45.6, 43.2, 25.5 ppm.
HRMS (ESI, pos): *m/z* $[M+H]^+$ calculated for $[C_{11}H_{16}N_2O_2]^+$: 166.0863, found: 166.0865.
IR (KBr): $\tilde{\nu}$ = 3576 (s), 3240 (s), 3096 (m), 2933 (s), 2827 (s), 1580 (m), 1526 (s), 1459 (m), 1388 (m), 1376 (s), 1294 (s), 1281 (s), 1185 (s), 1116 (m), 959 (w), 812 (m), 574 (m) cm^{-1}
UV / Vis (MeOH): λ_{max} = 290, 226, 213 nm.
mp: 256 - 261 °C.

2-(4-((4-Phenoxyphenyl)carbamoyl)phenoxy)acetic acid (10)

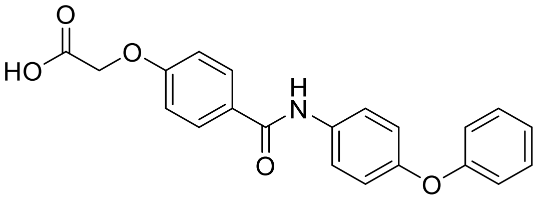

Synthesis of **10** has been described previously.[1]

4-(2-(6,7-Dihydroxy-3,4-dihydroisoquinolin-2(1*H*)-yl)-2-oxoethoxy)-*N*-(4-phenoxyphenyl)benzamide (11b)

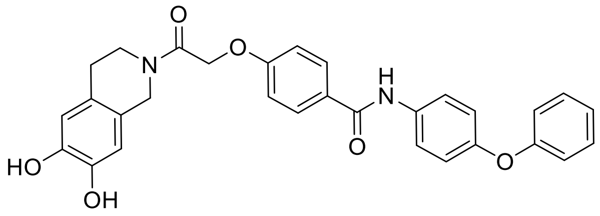

4-(2-(6,7-Dihydroxy-3,4-dihydroisoquinolin-2(1*H*)-yl)-2-oxoethoxy)-*N*-(4-phenoxyphenyl) benzamide **11b** was prepared from **9b** (150 mg, 0.610 mmol, 1.0 eq.) and 2-(4-((4-phenoxyphenyl)carbamoyl)phenoxy)acetic acid **10** (221 mg, 0.610 mmol, 1.0 eq.) according to **Method 7A**. Purification of the crude product by column chromatography (4 % MeOH in DCM) yielded **11b** as a colorless solid (190 mg, 61 %).

R_f = 0.21 (4 % MeOH in DCM, *v/v*).
^{1}H-NMR (400 MHz, CD_3OD, rotameric mixture): δ = 7.93 – 7.87 (m, 2H, R1/R2), 7.66 – 7.60 (m, 2H, R1/R2), 7.36 – 7.29 (m, 2H), 7.11 – 7.02 (m, 3H, R1/R2), 6.99 – 6.97 (m, 2H), 6.97 – 6.95 (m, 2H), 6.62 – 6.54 (m, 2H, R1/R2), 4.94 (s, 2H, R1/R2), 4.57 (s, 1H, R2), 4.54 (s, 1H, R1) 3.74 (t, ^3J = 6.0 Hz, 1H, R2), 3.70 (t, ^3J = 6.0 Hz, 1H, R1), 2.78 (t, ^3J = 5.9 Hz, 1H, R1), 2.69 (t, ^3J = 6.1 Hz, 1H, R2) ppm.
^{13}C-NMR (101 MHz, CD_3OD, rotameric mixture): δ = 168.81 (**C**=O, R2), 168.76 (**C**=O, R1), 168.2 (**C**=O), 162.5, 159.0, 155.1, 145.6 (R2), 145.4 (R1), 145.3 (R1), 145.2 (R2), 135.5, 130.9, 130.53 (R1), 130.48 (R2), 129.0 (R1), 128.9 (R2), 126.9 (R2), 126.4 (R1), 124.7 (R1), 124.4 (R2), 124.2, 124.1, 120.2, 119.5, 116.1 (R2), 116.0 (R1), 115.7 (R2), 115.6 (R1), 114.0 (R1), 113.9 (R2), 67.6 (R1), 67.5 (R2), 46.9 (R2), 45.1 (R1), 44.1 (R1), 41.8 (R2), 29.5 (R1), 28.5 (R2) ppm.
HRMS (ESI, pos): *m/z* $[M+H]^+$ calculated for $[C_{30}H_{27}N_2O_6]^+$: 511.1864, found: 511.1864.
IR (KBr): $\tilde{\nu}$ = 3433 (br), 2926 (w), 1644 (s), 1606 (s), 1529 (m), 1508 (s), 1489 (m), 1453 (w), 1247 (m), 1224 (s), 1178 (w), 873 (w), 762 (w) cm^{-1}.
UV / Vis (MeOH): λ_{max} = 279, 213 nm.
mp: 198 - 201 °C.

Tetrabenzyl (2-(2-(4-((4-phenoxyphenyl)carbamoyl)phenoxy)acetyl)-1,2,3,4-tetrahydroisoquinoline-6,7-diyl) bis(phosphate) (12b)

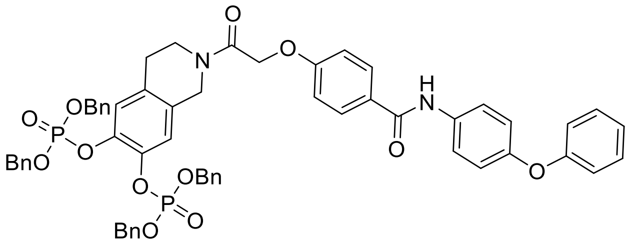

Tetrabenzyl (2-(2-(4-((4-phenoxyphenyl) carbamoyl) phenoxy) acetyl) -1,2,3,4-tetrahydroisoquinoline-6,7-diyl) bis(phosphate) **12b** was prepared from **11b** (45 mg, 0.088 mmol, 1.0 eq.) according to **Method 5**. Purification of the crude product by column chromatography (7:3 EtOAC / hexane, *v/v*) yielded **12b** as a colorless oil (51 mg, 56 %).

R_f = 0.30 (7:3 EtOAc / hexane, *v/v*).
^{1}H-NMR (400 MHz, $CDCl_3$, rotameric mixture): δ = 8.52 – 7.96 (m, 1H, N**H**, R1/R2), 7.85 (d, *J* = 8.2 Hz, 1H, R2), 7.76 (d, *J* = 8.4 Hz, 1H, R1), 7.65 (d, *J* = 8.8 Hz, 1H, R1), 7.59 (d, *J* = 8.6 Hz, 1H, R2), 7.38 – 7.19 (m, 22H), 7.12 – 6.94 (m, 7H), 6.88 – 6.82 (m, 2H, R1/R2), 5.14 – 5.03 (m, 8H, OC**H**$_2$Ph, R1/R2), 4.81 (s, 2H), 4.56 (s, 2H), 3.78 – 3.66 (m, 2H, R1/R2), 2.74 (t, *J* = 5.6 Hz, 1H, R2), 2.54 (t, *J* = 6.1 Hz, 1H, R1) ppm.
^{31}P-NMR (162 MHz, $CDCl_3$, rotameric mixture) δ = - 6.12 (R2), - 6.25 (R1), - 6.29 (R1/R2) ppm.
^{13}C-NMR (76 MHz, $CDCl_3$, rotameric mixture): δ = 166.6 (R1, **C**=O), 166.5 (R2, **C**=O), 165.4 (R1, **C**=O), 165.2 (R2, **C**=O), 160.7 (R2), 160.0 (R1), 157.8 (R1), 157.7 (R2), 153.6 (R2), 153.4 (R1), 140.3 – 139.7 (m, R1/R2), 135.5 – 135.2 (m), 134.3 (R1), 133.8 (R2), 132.3 (R1), 131.5 (R2), 130.3 (R2), 130.2 (R1), 129.8, 129.3, 128.9 (R1), 128.8 (R2), 128.74 (R1), 128.68 (R2), 128.43 (R2), 128.35 (R1), 128.2 (R1), 128.1 (R2), 123.2 (R2), 123.1 (R1), 122.1, 122.0 (R1), 121.5 (R2), 119.8 (R1), 119.7 (R2), 119.1 (R1/R2), 118.6 (R1), 118.5 (R2), 114.7 (R2), 114.6 (R1), 70.3 (d, $^{2}J_{C\text{-}P}$ = 5.8 Hz, O**C**H$_2$Ph), 67.9, 46.8 (R1), 44.1 (R2), 42.8 (R2), 40.6 (R1), 29.0 (R1), 27.6 (R2) ppm.
HRMS (ESI, pos): *m/z* $[M+H]^+$ calculated for $[C_{58}H_{53}N_2O_{12}P_2]^+$: 1031.3068, found: 1031.3062.
IR (KBr): $\tilde{\nu}$ = 3308 (w), 3007 (w), 1660 (s), 1605 (m), 1508 (s), 1489 (s), 1308 (m), 1282 (m), 1220 (s), 1178 (m), 1018 (s), 964 (m), 901 (m), 751 (s), 696 (m) cm^{-1}.
UV / Vis ($CHCl_3$): λ_{max} = 276 nm.

2-(2-(4-((4-Phenoxyphenyl)carbamoyl)phenoxy)acetyl)-1,2,3,4-tetrahydro-isoquinoline-6,7-diyl bis(dihydrogen phosphate) (8b)

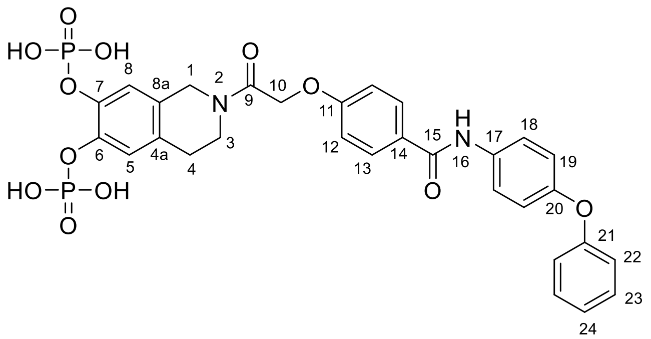

2-(2-(4-((4-Phenoxyphenyl)carbamoyl)phenoxy)acetyl)-1,2,3,4-tetrahydro-isoquinoline-6,7-diyl bis(dihydrogen phosphate) **8b** was prepared from **12b** (200 mg, 0.194 mmol, 1.0 eq.) according to **Method 8**. Lyophilization yielded **8b** as a colorless solid (129 mg, 99 %).

$\mathbf{R_f}$ = 0.23 (RP-TLC, 4:1 H_2O / CH_3CN, *v/v*).
^{1}H-NMR (400 MHz, D_2O, rotameric mixture): δ = 7.88 (d, *J* = 8.5 Hz, 2H, **H-13**), 7.56 – 7.49 (m, 2H, **H-18**), 7.49 – 7.42 (m, 2H, **H-23**), 7.27 – 7.20 (m, 3H, **H-8,5,24**), 7.17 – 7.07 (m, 6H, **H-12,19,22**), 5.13 (s, 1H, R1, **H-10**), 5.09 (s, 1H, R2, **H-10**), 4.71 (s, 1H, R1, **H-1**), 4.69 (s, 1H, R2, **H-1**), 3.85 – 3.73 (m, 2H, R1/R2, **H-3**), 3.02 – 2.94 (m, 1H, R2, **H-4**), 2.93 – 2.83 (m, 1H, R1, **H-4**) ppm.
^{31}P-NMR (162 MHz, D_2O, rotameric mixture) δ = -0.68 (R2), -0.83 (R1) ppm.
^{13}C-NMR (101 MHz, DMSO-d_6, rotameric mixture): δ = 165.98 (R2, **C-9**), 165.95 (R1, **C-9**), 164.8 (**C-15**), 160.7 (**C-11**), 157.4 (**C-21**), 151.9 (**C-20**), 141.2 – 140.4 (m, R1/R2, **C-6,7**), 135.2 (**C-17**), 130.8 (R2, **C-4a**), 130.6 (R1, **C-4a**), 130.0 (**C-23**), 129.4 (**C-13**), 129.2 (R1, **C-8a**), 128.9 (R2, **C-8a**), 127.3 (R1, **C-14**), 127.2 (R2, **C-14**), 123.0 (**C-24**), 122.0 (**C-18**), 121.9 (R2, **C-5**), 121.6 (R1, **C-5**), 119.7 (R1, **C-8**), 119.5 (R2, **C-8**), 119.3 (**C-19**), 117.9 (**C-22**), 114.3 (R1/R2, **C-12**), 65.9 (**C-10**), 44.7 (R2, **C-1**), 43.1 (R1, **C-1**), 41.6 (R1, **C-3**), 38.8 (R2, **C-3**), 28.1 (R1, **C-4**), 27.3 (R2, **C-4**) ppm.
HRMS (ESI, neg): *m/z* $[M-H]^-$ calculated for $[C_{30}H_{27}N_2O_{12}P_2]^-$: 669.1045, found: 669.1031.
IR (KBr): $\tilde{\nu}$ = 3477 (br), 3233 (m), 1638 (s), 1616 (s), 1508 (s), 1488 (m), 1248 (m), 1225 (m), 976 (w), 623 (br) cm^{-1}
UV / Vis (H_2O): λ_{max} = 272, 204 nm.
mp: 176 - 178°C.

POM-Prodrug (17)

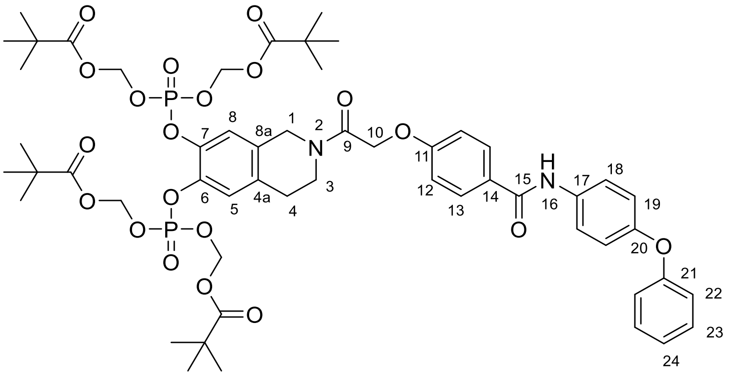

To a suspension of **8b** (100 mg, 0.149 mmol, 1 eq.) in dry CH_3CN (2 mL) was added DIPEA (260 µL, 1.49 mmol, 10 eq.) and POM-I (223 µL, 1.49 mmol, 10 eq.). The mixture was stirred overnight at room temperature and turned into a clear solution. The volatiles were removed in vacuo and the residue was purified by column chromatography (7:3 EtOAc / hexane, *v/v*) to yield prodrug **17** as a light-yellow foam / crystalline solid (60 mg, 36 %).

R_f = 0.29 (7:3 EtOAc / hexane, *v/v*).

^{1}H-NMR (400 MHz, CD_2Cl_2, rotameric mixture): δ = 8.27 – 7.90 (m, 1H, R1/R2, **H-16**), 7.87 – 7.82 (m, 1H, R1, **H-13**), 7.79 (d, *J* = 8.6 Hz, 1H, R2, **H-13**), 7.69 – 7.63 (m, 1H, R2, **H-18**), 7.63 – 7.57 (m, 1H, R1, **H-18**), 7.37 – 7.31 (m, 2H, **C-23**), 7.25 – 7.18 (m, 2H, R1/R2, **H-5,8**), 7.13 – 7.06 (m, 2H, R1, **H-12,24**), 7.05 – 6.97 (m, 4H, **H-19,22**), 6.93 (d, *J* = 8.7 Hz, 1H, R2, **H-12**), 5.78 – 5.67 (m, 8H, R1/R2, -OC**H₂**OC(O)C(CH₃)₃), 4.85 (s, 1H, R2, **H-10**), 4.84 (s, 1H, R1, **H-10**), 4.68 (s, 2H, R1/R2, **H-1**), 3.81 – 3.70 (m, 2H, R1/R2, **H-3**), 2.89 (t, *J* = 5.8 Hz, 1H, R1, **H-4**), 2.72 (t, *J* = 6.1 Hz, 1H, R2, **H-4**), 1.22 – 1.14 (m, 36H, -OCH₂OC(O)C**(CH₃)₃**) ppm.

^{31}P-NMR (162 MHz, CD_2Cl_2, rotameric mixture) δ = -9.53 (R2), -9.69 (R1/R2), -9.73 (R1) ppm.

^{13}C-NMR (76 MHz, CD_2Cl_2, rotameric mixture): δ = 176.9 (-OCH₂O**C**(O)C(CH₃)₃), 166.6 (R2, **C-9**), 166.5 (R1, **C-9**), 165.3 (R2, **C-15**), 165.1 (R1, **C-15**), 161.2 (R1, **C-11**), 160.7 (R2, **C-11**), 158.1 (R2, **C-21**), 158.0 (R1, **C-21**), 153.8 (R2, **C-20**), 153.7 (R1, **C-20**), 140.3 – 139.2 (m, R1/R2, **C-6,7**), 134.7 (R2, **C-17**), 134.4 (R1, **C-17**), 133.6 (R1, **C-4a**), 132.8 (R2, **C-4a**), 131.6 (R1, **C-8a**), 131.3 (R2, **C-8a**), 130.1 (**C-23**), 129.4 (**C-13**), 128.5 (**C-14**), 123.5 (R1, **C-24**), 123.4 (R2, **C-24**), 122.3 (**C-18**), 122.2 (R2, **C-5**), 121.9 (R1, **C-5**), 120.1 (R1, **C-8**), 119.89 (R1, **C-19**), 119.87 (R2, **C-19**), 119.6 (R2, **C-8**), 118.8 (**C-22**), 115.0 (**C-12**), 83.7 (R1, -O**C**H₂OC(O)C(CH₃)₃), 83.6 (R2, -O**C**H₂OC(O)C(CH₃)₃), 68.0 (R2, **C-10**), 67.9 (R1, **C-10**), 46.9 (R2, **C-1**), 44.3 (R1, **C-1**), 42.9 (R1, **C-3**), 40.5 (R2, **C-3**), 39.0 (-OCH₂OC(O)**C**(CH₃)₃), 29.4 (R1, **C-4**), 28.0 (R2, **C-4**), 26.9 (-OCH₂OC(O)C(**C**H₃)₃) ppm.

HRMS (ESI, pos): *m/z* $[M+H]^+$ calculated for $[C_{54}H_{69}N_2O_{20}P_2]^+$: 1127.3913, found: 1127.3959.

IR (KBr): ṽ = 3318 (w), 2978 (m), 1758 (s), 1659 (s), 1606 (m), 1508 (s), 1489 (s), 1308 (s), 1280 (s), 1224 (s), 1136 (s), 1056 (m), 1032 (m), 968 (s), 905 (m), 852 (w), 762 (m) cm^{-1}.
UV / Vis (H_2O): λ_{max} = 277 nm.
mp: 50 - 52 °C.

2,3,4,5-Tetrahydro-1*H*-benzo[*c*]azepine-7,8-diol hydrobromide (9c)

2,3,4,5-Tetrahydro-1*H*-benzo[c]azepine-7,8-diol hydrobromide **9c** was prepared essentially as described by *Zheng et al*.[17]

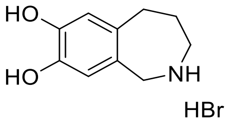

In brief, 7,8-dimethoxy-2,3,4,5-tetrahydro-1*H*-benzo[*c*]azepine **2c** (111 mg, 0.536 mmol, 1.0 eq.) was dissolved in HBr (48 % in H_2O, 2 mL). The reaction mixture was heated to 105 °C for 5 h and upon completion of the reaction concentrated in vacuo. The residue was suspended in EtOAc and again concentrated in vacuo to yield **9c** without further purification as a brown solid (123 mg, 88 %).

^{1}H-NMR (300 MHz, CD_3OD): δ = 6.78 (s, 1H), 6.69 (s, 1H), 4.20 (s, 2H), 3.45 – 3.38 (m, 2H), 2.92 – 2.86 (m, 2H), 1.99 – 1.89 (m, 2H) ppm.
^{13}C-NMR (101 MHz, CD_3OD): δ = 147.1, 144.7, 135.8, 124.0, 119.1, 117.9, 52.2, 51.6, 34.1, 27.0 ppm.
HRMS (ESI, neg): *m/z* $[M-H]^-$ calculated for $[C_{10}H_{12}NO_2]^-$: 178.0874, found: 178.0869.
IR (KBr): ṽ = 3498 (br), 3243 (w), 2976 (s), 2844 (m), 2731 (w), 1614 (s), 1572 (m), 1524 (s), 1465 (m), 1443 (m), 1292 (s), 1102 (m), 1073 (m), 884 (w), 852 (m), 775 (w) cm^{-1}.
UV / Vis ($CHCl_3$): λ_{max} = 286, 235, 212 nm.
mp: 82 - 85 °C.

4-(2-(7,8-Dihydroxy-1,3,4,5-tetrahydro-2*H*-benzo[*c*]azepin-2-yl)-2-oxoethoxy)-*N*-(4-phenoxyphenyl)benzamide (11c)

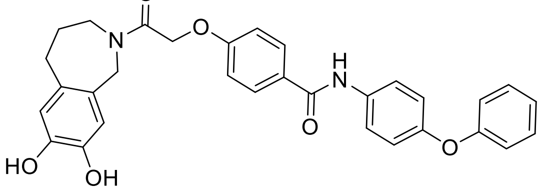

4-(2-(7,8-Dihydroxy-1,3,4,5-tetrahydro-2*H*-benzo[*c*]azepin-2-yl)-2-oxoethoxy)-*N*-(4-phenoxyphenyl)benzamide **11c** was prepared from **9c** (96 mg, 0.37 mmol, 1.0 eq.) and 2-(4-((4-phenoxyphenyl)carbamoyl)phenoxy)acetic acid **10** (135 mg, 0.371 mmol, 1.0 eq.) according to **Method 7A**. Purification of the crude product by column chromatography (4 % MeOH in DCM) yielded **11c** as a colorless solid (95 mg, 49 %).

R_f = 0.30 (4 % MeOH in DCM, *v/v*).
^{1}H-NMR (400 MHz, DMSO-d_6, rotameric mixture): δ = 10.12 – 10.08 (m, 1H, N**H**, R1/R2), 8.88 – 8.60 (m, 2H, O**H**, R1/R2), 7.91 – 7.82 (m, 2H, R1/R2), 7.81 – 7.73 (m, 2H, R1/R2), 7.42 – 7.34 (m, 2H), 7.15 – 7.08 (m, 1H), 7.07 – 6.93 (m, 5H), 6.86 – 6.80 (m, 1H), 6.80 – 6.53 (m, 2H, R1/R2), 4.92 (s, 1H, R2), 4.85 (s, 1H, R1), 4.46 (s, 1H, R1), 4.32 (s, 1H, R2), 3.76 – 3.65 (m, 1H, R1), 3.12 – 3.02 (m, 1H, R2), 2.82 – 2.74 (m, 2H, R1/R2), 1.78 – 1.70 (s, 1H, R2), 1.66 – 1.58 (s, 1H, R1) ppm.
^{13}C-NMR (75 MHz, DMSO-d_6, rotameric mixture): δ = 166.0 (R1, **C**=O), 165.1 (R2, **C**=O), 164.8 (R1, **C**=O), 164.7 (R2, **C**=O), 160.72 (R2), 160.68 (R1), 157.4, 151.9, 144.1 (R1), 143.7 (R2), 142.6 (R1), 142.3 (R2), 135.2, 132.6 (R1), 132.4 (R2), 130.0, 129.3, 128.5 (R2), 128.1 (R1), 127.19 (R2), 127.18 (R1), 123.0, 122.0, 119.3, 117.9, 117.6 (R1/R2), 117.1 (R2), 116.8 (R1), 114.2 (R2), 114.0 (R1), 66.0 (R1), 65.5 (R2), 50.4 (R1), 50.1 (R2), 49.9 (R1), 48.5 (R2), 33.7 (R2), 33.5 (R1), 29.3 (R2), 27.7 (R1) ppm.
HRMS (ESI, pos): *m/z* $[M+H]^+$ calculated for $[C_{31}H_{29}N_2O_6]^+$: 525.2020, found: 525.2040.
IR (KBr): $\tilde{\nu}$ = 3433 (br), 2935 (w), 1645 (s), 1606 (s), 1540 (m), 1508 (s), 1488 (s), 1407 (w), 1248 (s), 1224 (s), 1178 (m), 873 (m), 851 (m) cm^{-1}.
UV / Vis (MeOH): λ_{max} = 277, 209 nm.
mp: 245 - 247 °C.

Tetrabenzyl (2-(2-(4-((4-phenoxyphenyl)carbamoyl)phenoxy)acetyl)-2,3,4,5-tetrahydro-1*H*-benzo[*c*]azepine-7,8-diyl) bis(phosphate) (12c)

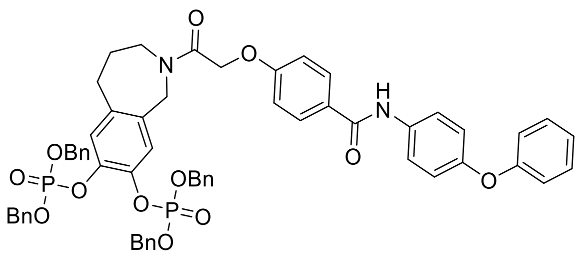

Tetrabenzyl (2-(2-(4-((4-phenoxyphenyl) carbamoyl) phenoxy) acetyl)-2,3,4,5-tetrahydro-1*H*-benzo[*c*]azepine-7,8-diyl) bis(phosphate) **12c** was prepared from **11c** (60 mg, 0.11 mmol, 1.0 eq.) according to **Method 5**. Purification of the crude product by column chromatography (7:3 EtOAC / hexane, *v/v*) yielded **12c** as a colorless oil (60 mg, 50 %).

R_f = 0.20 (7:3 EtOAc / hexane, *v/v*).
^{1}H-NMR (400 MHz, $CDCl_3$, rotameric mixture): δ = 9.32 – 8.88 (m, 1H, N**H**, R1/R2), 7.78 – 7.65 (m, 4H), 7.36 – 7.12 (m, 23H), 7.11 – 7.05 (m, 1H), 7.00 – 6.95 (m, 2H), 6.95 – 6.90 (m, 2H), 6.84 – 6.78 (m, 1H), 6.56 (d, *J* = 8.2 Hz, 1H, R1), 6.47 (d, *J* = 8.4 Hz, 1H, R2), 5.10 – 4.93 (m, 8H, OC**H**$_2$Ph, R1/R2), 4.74 – 4.68 (m, 2H, R1/R2), 4.52 (s, 1H, R2), 4.44 (s, 1H, R1), 3.88 – 3.78 (m, 2H, R1/R2), 2.88 – 2.80 (m, 1H, R1), 2.79 – 2.70 (m, 1H, R2), 1.86 – 1.80 (m, 1H, R2), 1.79 – 1.71 (m, 1H, R1) ppm.
^{31}P-NMR (162 MHz, $CDCl_3$, rotameric mixture) δ = -6.14 (R2), -6.34 (R1), -6.47 (R1), -6.62 (R2) ppm.
^{13}C-NMR (101 MHz, $CDCl_3$, rotameric mixture): δ = 167.6 (R2, **C**=O), 166.9 (R1, **C**=O), 165.6 (R1, **C**=O), 165.4 (R2, **C**=O), 160.6 (R1), 160.5 (R2), 157.9, 153.0, 140.9 – 138.7 (m, R1/R2), 135.6 – 134.1 (m), 134.9, 134.8, 129.82 (R2), 128.78 (R1), 129.52 (R1), 129.46 (R2), 129.0 (R1), 128.9 (R2), 128.80 (R2), 128.76 (R1), 128.74 (R1), 128.68 (R2), 128.2 (R2), 128.1 (R1), 127.8, 124.1 (R2), 123.7 (R1), 123.0 (R2), 122.9 (R1), 122.4 (R1), 122.3 (R1), 122.2 (R2), 122.0 (R2), 119.7 (R2), 119.5 (R1), 118.5 (R1), 118.4 (R2), 114.4 (R1), 114.1 (R2), 70.6 – 70.2 (m, R1/R2, O**C**H$_2$Ph), 68.1 (R2), 66.7 (R1), 52.0 (R1), 51.7 (R2), 50.5 (R1), 49.6 (R2), 34.5 (R2), 34.1 (R1), 29.3 (R1), 26.7 (R2) ppm.
HRMS (ESI, pos): *m/z* $[M+H]^+$ calculated for $[C_{59}H_{55}N_2O_{12}P_2]^+$: 1045.3225, found: 1045.3219.
IR (KBr): ṽ = 3434 (br), 3035 (w), 2931 (w), 1664 (s), 1605 (m), 1508 (s), 1489 (m), 1293 (m), 1222 (s), 1177 (w), 1016 (s), 962 (m), 907 (w), 741 (m), 696 (m) cm^{-1}.
UV / Vis (DCM): λ_{max} = 274, 228 nm.

2-(2-(4-((4-Phenoxyphenyl)carbamoyl)phenoxy)acetyl)-2,3,4,5-tetrahydro-1*H*-benzo[*c*]azepine-7,8-diyl bis(dihydrogen phosphate) (8c)

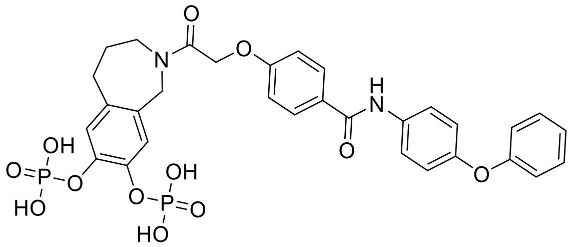

2-(2-(4-((4-Phenoxyphenyl)carbamoyl)phenoxy)acetyl)-2,3,4,5-tetrahydro-1*H*-benzo[*c*]azepine-7,8-diyl bis(dihydrogen phosphate) **8c** was prepared from **12c** (30 mg, 0.029 mmol, 1.0 eq.) according to **Method 8**. After filtration, the crude product was extracted with CH_3CN and washed with pentane (1 x). The solvent was removed *in vacuo*, and the residue was suspended in water. Lyophilization yielded **8c** as a colorless solid (20 mg, quant.).

R_f = 0.55 (RP-TLC, 3:2 H_2O / CH_3CN, *v/v*).
^{1}H-NMR (400 MHz, DMSO-d_6, rotameric mixture): δ = 7.90 – 7.79 (m, 2H), 7.77 – 7.68 (m, 2H), 7.42 – 7.28 (m, 2H), 7.21 – 7.05 (m, 3H), 6.97 (t, *J* = 9.7 Hz, 5H), 6.78 (d, *J* = 8.4 Hz, 1H), 4.89 (s, 1H, R2), 4.85 (s, 1H, R1), 4.58 (s, 1H, R2), 4.42 (s, 1H, R1), 3.77 – 3.65 (m, 2H, R1/R2), 2.97 – 2.81 (m, 2H, R1/R2), 1.85 – 1.73 (m, 1H, R1), 1.72 – 1.59 (m, 1H, R2) ppm.
^{31}P-NMR (162 MHz, DMSO-d_6, rotameric mixture): δ = -6.03 (R2), -6.14 (R1), -6.21 (R2), -6.24 (R1) ppm.
^{13}C-NMR (101 MHz, DMSO-d_6, rotameric mixture): δ = 166.2 (R2, **C**=O), 165.4 (R1, **C**=O), 164.7 (**C**=O), 160.6 (R1), 160.5 (R2), 157.3, 151.82 (R1), 151.79 (R2), 141.8 – 141.5 (m, R2), 141.4 – 141.0 (m, R1), 140.7 – 140.3 (m, R1), 140.3 – 139.7 (m, R2), 138.0, 135.22 (R2), 135.17 (R1), 134.1 (R1), 133.6 (R2), 129.9, 129.3, 127.2 (R1), 127.0 (R2), 123.9 (R1), 123.3 (R2), 123.0 (R1), 122.9, 122.8 (R2), 121.92 (R1), 121.89 (R2), 119.2, 117.8, 114.2 (R1), 114.0 (R2), 65.8 (R1), 65.2 (R2), 50.0 (R1/R1), 49.6 (R2), 48.3 (R2), 33.7 (R2), 33.5 (R1), 28.7 (R2), 27.1 (R1) ppm.
HRMS (ESI, neg): *m/z* $[M-H]^-$ calculated for $[C_{31}H_{29}N_2O_{12}P_2]^-$: 683.1201, found: 683.1188.
IR (KBr): $\tilde{\nu}$ = 3434 (br), 2931 (w), 1638 (s), 1607 (s), 1508 (s), 1489 (m), 1385 (w), 1307 (w), 1247 (m), 1224 (m), 1179 (w), 964 (w), 849 (w) cm^{-1}.
UV / Vis (H_2O): λ_{max} = 271, 201 nm.
mp: 171 - 177 °C.

4-(2-(5,6-Dihydroxyisoindolin-2-yl)-2-oxoethoxy)-*N*-(4-phenoxyphenyl) benzamide (11a)

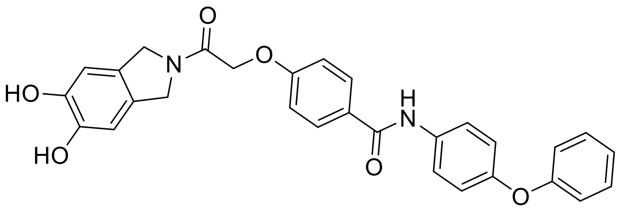

4-(2-(5,6-Dihydroxyisoindolin-2-yl)-2-oxoethoxy)-*N*-(4-phenoxyphenyl) benzamide **11a** was prepared from 2,3-dihydro-1*H*-isoindole-5,6-diol hydrobromide **9a** (65 mg, 0.28 mmol, 1.0 eq.) and 2-(4-((4-phenoxyphenyl)carbamoyl)phenoxy)acetic acid **10** (102 mg, 0.281 mmol, 1.0 eq.) according to **Method 7B**. Purification of the crude product by column chromatography (5 % MeOH in DCM) yielded **11a** as a colorless solid (92 mg, 66 %).

R_f = 0.35 (5 % MeOH in DCM, *v/v*).
^{1}H-NMR (400 MHz, DMSO-d_6, rotameric mixture): δ = 10.12 (s, 1H, N**H**), 8.97 (br, 2H, O**H**), 7.95 – 7.88 (m, 2H), 7.80 – 7.74 (m, 2H), 7.41 – 7.34 (m, 2H), 7.13 – 7.05 (m, 3H), 7.04 – 7.00 (m, 2H), 7.00 – 6.96 (m, 2H), 6.72 (s, 1H), 6.70 (s, 1H), 4.94 (s, 2H), 4.75 (s, 2H), 4.52 (s, 2H) ppm.
^{13}C-NMR (101 MHz, DMSO-d_6, rotameric mixture): δ = 165.8, 164.9, 160.8, 157.4, 152.0, 145.31, 145.30, 135.2, 130.0, 129.4, 127.3, 126.7, 125.8, 123.0, 122.1, 119.3, 118.0, 114.3, 109.7, 109.4, 65.7, 51.8, 50.5 ppm.
HRMS (ESI, pos): *m/z* $[M+Na]^+$ calculated for $[C_{29}H_{24}N_2O_6Na]^+$: 519.1527, found: 519.1507.
IR (KBr): $\tilde{\nu}$ = 3435 (br), 2856 (w), 1644 (s), 1607 (m), 1508 (s), 1489 (m), 1461 (w), 1352 (w), 1227 (s), 1178 (w), 873 (w) cm^{-1}.
UV / Vis (MeOH): λ_{max} = 280, 205 nm.
mp: 244 - 247 °C.

Tetrabenzyl (2-(2-(4-((4-phenoxyphenyl)carbamoyl)phenoxy)acetyl)isoindoline-5,6-diyl) bis(phosphate) (12a)

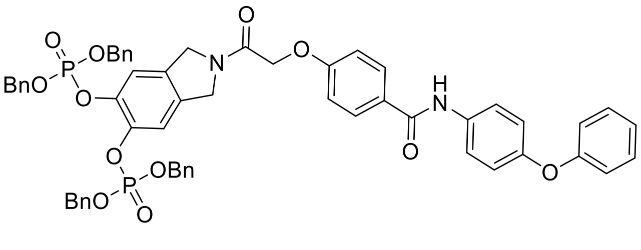

Tetrabenzyl (2-(2-(4-((4-phenoxyphenyl) carbamoyl) phenoxy) acetyl) isoindoline-5,6-diyl) bis(phosphate) **12a** was prepared from **11a** (30 mg, 0.060 mmol, 1.0 eq.) in dry DMF (250 µL) and dry CH_3CN (250 µL) according to **Method 5**. Purification of the crude product by column chromatography (7:3 EtOAC / hexane, *v/v*) yielded **12a** as a colorless oil (17 mg, 30 %).

R_f = 0.30 (7:3 EtOAc / hexane, *v/v*).

^{1}H-NMR (400 MHz, CD_2Cl_2, rotameric mixture): δ = 8.41 (s, 1H, N**H**), 7.89 (d, *J* = 8.4 Hz, 2H), 7.65 (d, *J* = 8.5 Hz, 2H), 7.38 – 7.22 (m, 22H), 7.20 (s, 1H), 7.17 (s, 1H), 7.10 (t, *J* = 7.4 Hz, 1H), 7.04 – 6.94 (m, 6H), 5.10 (d, $^{3}J_{P\text{-}H}$ = 8.5 Hz, 8H, OC**H**$_2$Ph), 4.76 (s, 2H), 4.74 (s, 2H), 4.66 (s, 2H) ppm.

^{31}P-NMR (162 MHz, CD_2Cl_2, rotameric mixture) δ = -6.23 ppm.

^{13}C-NMR (101 MHz, CD_2Cl_2, rotameric mixture): δ = 166.4, 165.3, 161.1, 158.1, 153.7, 141.9 – 141.5 (m), 135.9 (d, $^{3}J_{C\text{-}P}$ = 7.0 Hz), 134.7, 133.9, 133.5, 130.1, 129.6, 129.1 (d, *J* = 2.5 Hz), 128.98 (R1), 128.97 (R2), 128.6, 128.4, 123.4, 122.4, 119.8, 118.8, 116.6 (d, *J* = 2.2 Hz), 116.4 (d, *J* = 1.9 Hz), 114.8, 70.7 (d, $^{2}J_{C\text{-}P}$ = 5.9 Hz, O**C**H$_2$Ph), 67.6, 52.6, 51.6 ppm.

HRMS (ESI, pos): *m/z* $[M+H]^+$ calculated for $[C_{57}H_{51}N_2O_{12}P_2]^+$: 1017.2912, found: 1017.2914.

IR (KBr): ṽ = 3435 (br), 2925 (w), 1659 (s), 1606 (m), 1507 (s), 1489 (m), 1359 (w), 1305 (m), 1222 (s), 1177 (w), 1016 (s), 959 (m), 904 (w), 740 (m), 695 (m) cm^{-1}.

UV / Vis ($CHCl_3$): λ_{max} = 277, 244 nm.

2-(2-(4-((4-Phenoxyphenyl)carbamoyl)phenoxy)acetyl)isoindoline-5,6-diyl bis(dihydrogen phosphate) (8a)

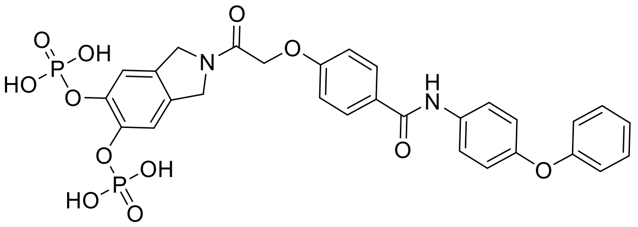

2-(2-(4-((4-Phenoxyphenyl)carbamoyl)phenoxy)acetyl)isoindoline-5,6-diyl bis (dihydrogen phosphate) **8a** was prepared from **12a** (30 mg, 0.029 mmol, 1.0 eq.) according to **Method 8**. Lyophilization yielded **8a** as a colorless solid (19 mg, quant).

R_f = 0.58 (RP-TLC, 3:2 H_2O / CH_3CN, *v/v*).

^{1}H-NMR (400 MHz, DMSO-d_6, rotameric mixture): δ = 10.13 (s, 1H, N**H**), 7.92 (d, *J* = 8.6 Hz, 2H), 7.77 (d, *J* = 8.9 Hz, 2H), 7.41 – 7.31 (m, 4H), 7.14 – 7.06 (m, 3H), 7.02 (d, *J* = 8.9 Hz, 2H), 6.98 (d, *J* = 8.1 Hz, 2H), 4.97 (s, 2H), 4.88 (s, 2H), 4.65 (s, 2H) ppm.

^{31}P-NMR (162 MHz, DMSO-d_6, rotameric mixture): δ = - 6.24 ppm.

^{13}C-NMR (76 MHz, DMSO-d_6, rotameric mixture): δ = 165.9, 164.9, 160.8, 157.4, 152.0, 142.5 (t, *J* = 6.1 Hz), 135.3, 132.5, 131.6, 130.0, 129.4, 127.3, 123.1, 122.1, 119.3, 118.0, 116.6, 116.4, 114.4, 65.8, 51.7, 50.4 ppm.

HRMS (ESI, neg): *m/z* $[M-H]^-$ calculated for $[C_{29}H_{25}N_2O_{12}P_2]^-$: 655.0888, found: 655.0860.

IR (KBr): ṽ = 3434 (br), 1645 (s), 1607 (m), 1508 (s), 1489 (m), 1361 (w), 1309 (w), 1248 (m), 1226 (s), 1180 (w), 973 (m), 873 (w) cm^{-1}.
UV / Vis (H_2O): λ_{max} = 273, 202 nm.
mp: 189 - 191°C.

(4-((4-Phenoxyphenyl)carbamoyl)phenyl)glycine (13a)

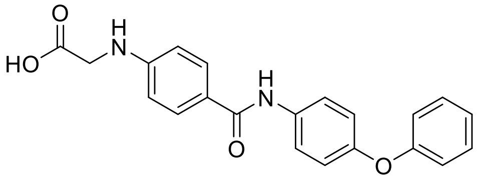

Synthesis of **13a** has been described previously.[18]

4-((2-(6,7-Dihydroxy-3,4-dihydroisoquinolin-2(1*H*)-yl)-2-oxoethyl)amino)-*N*-(4-phenoxyphenyl)benzamide (13b)

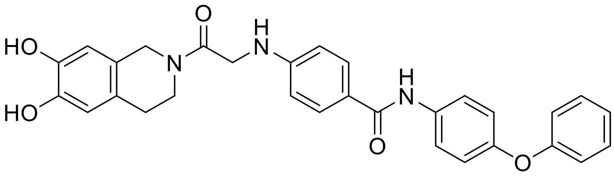

4-((2-(6,7-Dihydroxy-3,4-dihydroisoquinolin-2(1*H*)-yl)-2-oxoethyl)amino)-*N*-(4-phenoxy phenyl)benzamide **13b** was synthesized from **9b** (100 mg, 0.407 mmol, 1.0 eq.) and (4-((4-phenoxyphenyl)carbamoyl)phenyl)glycine **13a** (107 mg, 0.407 mmol, 1.0 eq.) according to **Method 7B**. Purification of the crude product by column chromatography (5 % MeOH in DCM) yielded **13b** as an off-white solid (91 mg, 44 %).

R_f = 0.25 (5 % MeOH in DCM, *v/v*).
^{1}H-NMR (400 MHz, DMSO-d_6, rotameric mixture): δ = 9.90 – 9.78 (m, 1H, N**H**), 8.79 (s, 2H, O**H**), 7.82 – 7.70 (m, 4H), 7.41 – 7.32 (m, 2H), 7.14 – 7.06 (m, 1H), 7.04 – 6.94 (m, 4H), 6.78 – 6.70 (m, 2H, R1/R2), 6.60 – 6.51 (s, 2H, R1/R2), 6.31 – 6.23 (m, 1H, N**H**), 4.54 (s, 1H, R2), 4.45 (s, 1H, R1), 4.11 – 4.03 (m, 2H, R1/R2), 3.70 – 3.62 (m, 2H, R1/R2), 2.71 (t, ^3J = 5.9 Hz, 1H, R1), 2.60 (t, ^3J = 5.9 Hz, 1H, R2) ppm.
^{13}C-NMR (76 MHz, DMSO-d_6, rotameric mixture): δ = 167.8 (**C**=O, R2), 167.7 (**C**=O, R1), 165.3 (**C**=O), 157.5, 151.6, 151.1, 144.1 (R2), 144.0 (R1), 143.90 (R1), 143.89 (R2), 135.7, 130.0, 129.2, 125.0 (R2), 124.8 (R1), 123.5 (R1), 123.2 (R2), 123.0, 121.9, 121.6, 119.3, 117.9, 115.28 (R2), 115.24 (R1), 113.3 (R1), 113.2 (R2), 111.5 (R1/R2), 44.9 (R2), 44.6 (R1/R2), 44.5 (R1), 43.5 (R2), 42.1 (R1), 28.0 (R1), 27.3 (R2) ppm.

HRMS (ESI, pos): *m/z* $[M+H]^+$ calculated for $[C_{30}H_{28}N_3O_5]^+$: 510.2023, found: 510.2039.
IR (KBr): $\tilde{\nu}$ = 3397 (br), 2927 (w), 1645 (s), 1607 (s), 1508 (s), 1488 (s), 1442 (s), 1408 (s), 1315 (s), 1224 (s), 1187 (s), 1101 (m), 872 (s), 834 (m), 762 (m), 693 (m) cm^{-1}.
UV / Vis (MeOH): λ_{max} = 306, 209 nm.
mp: 196 - 199 °C

Tetrabenzyl (2-((4-((4-phenoxyphenyl)carbamoyl)phenyl)glycyl)-1,2,3,4-tetrahydroisoquinoline-6,7-diyl) bis(phosphate) (13c)

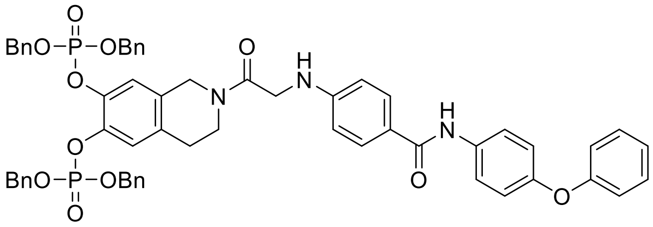

Tetrabenzyl (2-((4-((4-phenoxyphenyl)carbamoyl)phenyl)glycyl)-1,2,3,4-tetrahydroisoquinoline-6,7-diyl) bis(phosphate) **13c** was prepared from **13b** (70 mg, 0.14 mmol, 1.0 eq.) in dry CH_3CN / DMF (1 mL / 0.6 mL) according to **Method 5**. Purification of the crude product by column chromatography (3:2 → 9:1 EtOAC / hexane, *v/v*) yielded **13c** as a colorless oil / colorless foam (81 mg, 57 %).

R_f = 0.25 (8:2 EtOAc / hexane, *v/v*).
^{1}H-NMR (400 MHz, $CDCl_3$, rotameric mixture) δ = 8.26 – 8.21 (m, 1H, R1/R2, N**H**), 7.79 (d, *J* = 8.2 Hz, 2H), 7.63 (d, *J* = 8.5 Hz, 2H), 7.36 – 7.19 (m, 22H), 7.13 – 7.02 (m, 2H), 7.02 – 6.94 (m, 5H), 6.61 (d, *J* = 8.3 Hz, 2H), 5.10 (d, $^3J_{P\text{-}H}$ = 8.3 Hz, 4H, OC**H**$_2$Ph), 5.08 (d, $^3J_{P\text{-}H}$ = 8.2 Hz, 4H, OC**H**$_2$Ph), 4.59 (s, 1H, R1), 4.38 (s, 1H, R2), 3.94 (s, 1H, R1), 3.90 (s, 1H, R2), 3.79 (t, *J* = 6.0 Hz, 1H, R2), 3.57 (t, *J* = 5.8 Hz, 1H, R1), 2.75 (t, *J* = 5.9 Hz, 1H, R1), 2.69 (t, *J* = 6.0 Hz, 1H, R2) ppm.
^{31}P NMR (162 MHz, $CDCl_3$, rotameric mixture) δ = -6.19 (R2), -6.27 (R1) ppm.
^{13}C-NMR (101 MHz, $CDCl_3$) δ = 167.5 (**C**=O, R2), 167.4 (**C**=O, R1), 165.7 (**C**=O), 157.8, 153.1, 150.0, 140.3 – 139.8 (m, R1/R2), 135.4 (d, $J_{P\text{-}C}$ = 7.0 Hz, R2), 135.3 (d, $J_{P\text{-}C}$ = 7.0 Hz, R1), 134.4, 132.5 (R2), 131.4 (R1), 130.4 (R2), 129.81 (R1), 129.78, 129.2, 128.80 (R1), 128.75 (R2), 128.69 (R1), 128.66 (R2), 128.13 (R2), 128.12 (R1), 123.21 (R2), 123.16 (R1), 123.0, 122.1, 121.9 (R2), 121.4 (R1), 119.7, 119.6 (R1), 119.2 (R2), 118.4, 112.22 (R2), 112.18 (R1), 70.4 (d, $^2J_{P\text{-}C}$ = 5.8 Hz, O**C**H$_2$Ph, R2), 70.3 (d, $^2J_{P\text{-}C}$ = 5.9 Hz, O**C**H$_2$Ph, R1), 45.3 (R1), 45.0 (R2), 44.8 (R1), 44.0 (R2), 41.7 (R1), 39.9 (R2), 28.6 (R1), 27.8 (R2) ppm.
HRMS (ESI, pos) *m/z* $[M+H]^+$ calculated for $[C_{58}H_{54}N_3O_{11}P_2]^+$: 1030.3228, found: 1030.3204.

IR (KBr): $\tilde{\nu}$ = 3443 (br), 3033 (w), 1652 (s), 1607 (s), 1508 (s), 1488 (m), 1309 (m), 1279 (s), 1222 (s), 1196 (m), 1015 (s), 964 (m), 900 (w), 741 (m), 696 (m) cm^{-1}.

UV / Vis ($CHCl_3$): λ_{max} = 304 nm.

2-((4-((4-Phenoxyphenyl)carbamoyl)phenyl)glycyl)-1,2,3,4-tetrahydroisoquinoline-6,7-diyl bis(dihydrogen phosphate) (13)

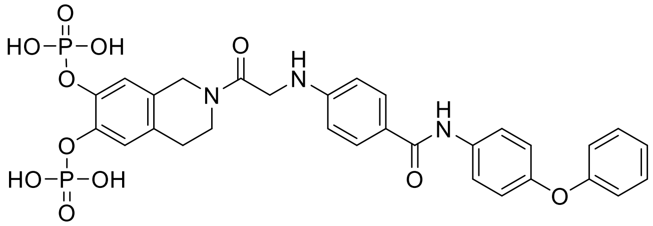

2-((4-((4-Phenoxyphenyl)carbamoyl)phenyl)glycyl)-1,2,3,4-tetrahydroisoquinoline-6,7-diyl bis(dihydrogen phosphate) **13** was prepared from **13c** (20 mg, 0.019 mmol, 1.0 eq.) according to **Method 6**. The crude product was purified by reversed-phase column chromatography (100 % H_2O). Lyophilization yielded **13** as a colorless solid (10 mg, 77 %).

$\mathbf{R_f}$ = 0.57 (RP-TLC, 3:2 H_2O / CH_3CN, *v/v*).

^{1}H-NMR (400 MHz, D_2O, rotameric mixture): δ = 7.75 – 7.66 (m, 2H), 7.51 – 7.34 (m, 4H), 7.26 – 7.13 (m, 3H), 7.11 – 6.98 (m, 4H), 6.72 (d, *J* = 8.3 Hz, 2H), 4.69 (s, 1H, R1), 4.64 (s, 1H, R2), 4.16 (s, 1H, R1), 4.13 (s, 1H, R2), 3.81 – 3.69 (m, 2H, R1/R2), 2.97 – 2.90 (m, 1H, R1), 2.88 – 2.80 (m, 1H, R2) ppm.

^{31}P-NMR (162 MHz, D_2O, rotameric mixture): δ = - 3.51 ppm.

^{13}C-NMR (101 MHz, D_2O, rotameric mixture): δ = 170.6, 169.0, 156.8, 153.8, 151.3, 141.9 – 141.4 (m, R1/R2), 132.9, 131.1 (R2), 130.8 (R1), 130.0, 129.3, 128.6 (R1), 128.1 (R2), 124.4 (R1), 124.3 (R2), 123.7, 121.8, 121.5 (R1), 121.3 (R2), 119.5 (R1), 119.4 (R2), 119.3, 118.5, 112.4, 45.6 (R1), 44.7 (R1, **C**H_2-NH), 44.5 (R2, **C**H_2-NH), 44.0 (R2), 42.3 (R1), 40.6 (R2), 27.8 (R2), 27.1 (R1) ppm.

HRMS (ESI, neg): *m/z* $[M-H]^-$ calculated for $[C_{30}H_{28}N_3O_{11}P_2]^-$: 668.1205, found: 668.1226.

IR (KBr): $\tilde{\nu}$ = 3443 (br), 1639 (m), 1611 (m), 1509 (m), 1489 (w), 1402 (w), 1385 (m), 1312 (w), 1226 (w), 1092 (w), 923 (w) cm^{-1}.

UV / Vis (H_2O): λ_{max} = 288, 200 nm.

mp: 149 °C (decomposition).

Ethyl 5-((4-phenoxyphenyl)carbamoyl)-1*H*-indole-2-carboxylate (14b)

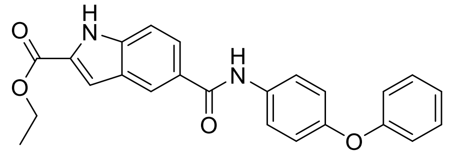

Ethyl 5-((4-phenoxyphenyl)carbamoyl)-1*H*-indole-2-carboxylate **14b** was prepared from 2-(ethoxycarbonyl)-1*H*-indole-5-carboxylic acid **14a** (200 mg, 0.858 mmol, 1.00 eq.) and 4-phenoxyaniline (159 mg, 0.858 mmol, 1.00 eq.) according to **Method 7A**. After stirring overnight, the solvent was removed under reduced pressure, and the crude product was purified by column chromatography (7:3 hexane / EtOAc). The product **14b** was obtained as a colorless solid (288 mg, 84 %).

$\mathbf{R_f}$ = 0.23 (7:3 hexane / EtOAc, *v/v*).
^{1}H-NMR (400 MHz, DMSO-d_6): δ = 12.18 (s, 1H, N**H**), 10.25 (s, 1H, N**H**), 8.38 – 8.36 (m, 1H), 7.88 (dd, *J* = 8.8, 1.7 Hz, 1H), 7.84 – 7.79 (m, 2H), 7.54 (d, *J* = 8.7 Hz, 1H), 7.41 – 7.35 (m, 2H), 7.31 (s, 1H), 7.14 – 7.08 (m, 1H), 7.06 – 7.01 (m, 2H), 7.02 – 6.97 (m, 2H), 4.37 (q, *J* = 7.1 Hz, 2H, $OC\mathbf{H_2}CH_3$), 1.36 (t, *J* = 7.1 Hz, 3H, $OCH_2C\mathbf{H_3}$) ppm.
^{13}C-NMR (101 MHz, DMSO-d_6): δ = 165.9, 161.1, 157.4, 151.8, 138.8, 135.4, 130.0, 128.9, 127.2, 126.1, 124.3, 123.0, 122.5, 122.0, 119.3, 117.9, 112.3, 108.8, 60.7, 14.3 ppm.
HRMS (ESI, pos): *m/z* $[M+H]^+$ calculated for $[C_{24}H_{21}N_2O_4]^+$: 401.1496, found: 401.1492.
IR (KBr): ṽ = 3432 (m), 2980 (w), 1703 (m), 1650 (m), 1524 (m), 1508 (s), 1489 (m), 1406 (w), 1333 (m), 1251 (m), 1225 (m), 1190 (m), 1020 (w), 758 (w), 692 (w) cm^{-1}.
UV / Vis (MeOH): λ_{max} = 310, 256, 204 nm.
mp: 233 - 234 °C.

5-((4-Phenoxyphenyl)carbamoyl)-1*H*-indole-2-carboxylic acid (14c)

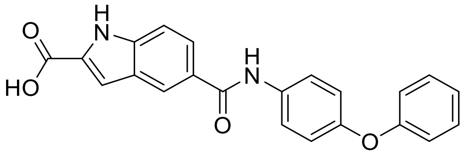

To a solution of **14b** (78 mg, 0.20 mmol, 1.0 eq.) in THF (2 mL) was added NaOH (1 M, 2 mL) and the mixture was refluxed for 2 h. The reaction was neutralised by the addition of 1 M HCl and extracted with EtOAc (3 x 10 mL). The combined organic layers were washed with brine

(1 x 20 mL), dried over Na_2SO_4, filtered and concentrated in vacuo to yield **14c** as a colorless solid (66 mg, 90 %).

^{1}H-NMR (400 MHz, DMSO-d$_6$): δ = 12.05 (s, 1H, N**H**), 10.23 (s, 1H, N**H**), 8.36 (s, 1H), 7.86 (dd, *J* = 8.7, 1.7 Hz, 1H), 7.84 – 7.79 (m, 2H), 7.52 (d, *J* = 8.7 Hz, 1H), 7.41 – 7.35 (m, 2H), 7.24 (d, *J* = 1.7 Hz, 1H), 7.13 – 7.08 (m, 1H), 7.06 – 7.01 (m, 2H), 7.01 – 6.96 (m, 2H) ppm.
^{13}C-NMR (101 MHz, DMSO-d$_6$): δ = 166.0, 162.6, 157.4, 151.8, 138.7, 135.5, 130.1, 130.0, 126.9, 126.2, 124.0, 123.0, 122.4, 122.0, 119.3, 117.9, 112.2, 108.4 ppm.
HRMS (ESI, pos): *m/z* $[M+H]^+$ calculated for $[C_{22}H_{17}N_2O_4]^+$: 373.1183, found: 373.1184.
IR (KBr): ṽ = 3421(br), 2925 (w), 1700 (m), 1641 (w), 1520 (s), 1508 (s), 1489 (s), 1408 (m), 1332 (m), 1249 (s), 1226 (s), 1184 (m), 871 (w), 759 (m), 692 (w) cm^{-1}.
UV / Vis (MeOH): λ_{max} = 308, 254, 204 nm.
mp: 295°C (decomposition).

2-(6,7-Dihydroxy-1,2,3,4-tetrahydroisoquinoline-2-carbonyl)-*N*-(4-phenoxyphenyl)-1*H*-indole-5-carboxamide (14d)

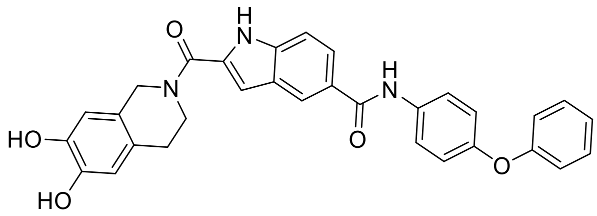

2-(6,7-Dihydroxy-1,2,3,4-tetrahydroisoquinoline-2-carbonyl)-*N*-(4-phenoxyphenyl)-1*H*-indole-5-carboxamide **14d** was synthesized from **14c** (50 mg, 0.13 mmol, 1.0 eq.) and **9b** (33 mg, 0.13 mmol, 1.0 eq.) according to **Method 7A**. After stirring overnight, the solvent was removed under reduced pressure and the crude product was purified by column chromatography (3 % MeOH in DCM, *v/v*) yielded **14d** as an off-white solid (32 mg, 46 %).

R$_f$ = 0.18 (3 % MeOH in DCM, *v/v*).
^{1}H-NMR (400 MHz, DMSO-d$_6$, rotameric mixture, recorded at 5°C): δ = 11.97 (s, 1H, N**H**), 10.28 (s, 1H, N**H**), 8.33 (s, 1H), 7.87 – 7.78 (m, 3H), 7.51 (d, *J* = 8.7 Hz, 1H), 7.40 – 7.33 (m, 2H), 7.09 (t, *J* = 7.4 Hz, 1H), 7.06 – 7.00 (m, 3H, R1/R2), 7.00 – 6.95 (m, 2H), 6.64 – 6.55 (m, 2H, R1/R2), 4.91 (s, 1H, R2), 4.59 (s, 1H, R1), 4.02 – 3.93 (m, 1H, R1), 3.89 – 3.77 (m, 1H, R2), 2.86 – 2.76 (m, 1H, R1), 2.76 – 2.66 (m, 1H, R2) ppm.
^{13}C-NMR (76 MHz, DMSO-d$_6$, rotameric mixture, recorded at 5°C): δ = 165.9 (**C**=O, R2), 165.8 (**C**=O, R1), 161.7 (**C**=O), 157.2, 151.4, 143.6 (R1), 143.5 (R2), 137.2 (R2), 137.1 (R1), 135.2

(R2), 135.1 (R1), 131.3 (R2), 131.2 (R1), 129.7, 126.34 (R2), 126.30 (R1), 126.0 (R1/R2), 124.6 (R1/R2), 123.0, 122.8 (R1/R2), 122.6, 121.7 (R1/R2), 121.6 (R2), 121.5 (R1), 119.1, 117.5, 114.8 (R1/R2), 113.0 (R1/R2), 111.5, 105.3 (R2), 104.5 (R1), 47.9 (R2), 44.9 (R1), 44.2 (R1), 40.6 (R2), 28.0 (R1), 26.7 (R2) ppm.

HRMS (ESI, pos): *m/z* $[M+H]^+$ calculated for $[C_{31}H_{26}N_3O_5]^+$: 520.1867, found: 520.1875.

IR (KBr): $\tilde{\nu}$ = 3446 (br), 2928 (w), 1641 (s), 1614 (s), 1524 (s), 1508 (s), 1488 (s), 1438 (m), 1407 (m), 1320 (w), 1285 (m), 1225 (s), 1189 (m), 1098 (w), 870 (w), 753 (m), 692 (w) cm^{-1}.

UV / Vis (CH_3CN): λ_{max} = 254, 206 nm.

mp: 264 - 266°C.

Tetrabenzyl (2-(5-((4-phenoxyphenyl)carbamoyl)-1*H*-indole-2-carbonyl)-1,2,3,4-tetrahydroisoquinoline-6,7-diyl) bis(phosphate) (14e)

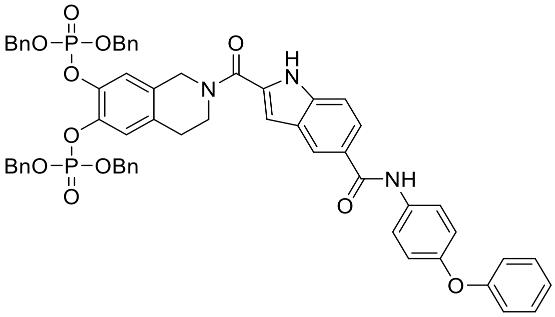

Tetrabenzyl (2-(5-((4-phenoxyphenyl)carbamoyl)-1*H*-indole-2-carbonyl)-1,2,3,4-tetrahydroisoquinoline-6,7-diyl) bis(phosphate) **14e** was synthesized from **14d** (80 mg, 0.15 mmol, 1.0 eq.) according to **Method 5**. Instead of CH_3CN dry DMF was used as solvent. The crude product was purified by column chromatography (7:3 EtOAc / hexane) to yield **14e** as a yellow oil (57 mg, 36 %).

R_f = 0.30 (7:3 hexane / EtOAc, *v/v*).

^{1}H-NMR (400 MHz, CD_2Cl_2, rotameric mixture): δ = 10.35 (s, 1H, N**H**), 8.55 (s, 1H, N**H**), 8.31 (s, 1H), 7.81 (dd, *J* = 8.7, 1.7 Hz, 1H), 7.74 – 7.68 (m, 2H), 7.44 (d, *J* = 8.7 Hz, 1H), 7.38 – 7.31 (m, 2H), 7.31 – 7.19 (m, 20H), 7.10 (t, *J* = 7.4 Hz, 1H), 7.07 – 7.03 (s, 2H), 7.03 – 6.96 (m, 4H), 6.88 (s, 1H), 5.11 (d, $^3J_{H-P}$ = 8.4 Hz, 4H, OC**H**$_2$Ph, R1), 5.09 (d, $^3J_{H-P}$ = 8.4 Hz, 4H, OC**H**$_2$Ph, R2), 4.78 (s, 2H, R1/R2), 4.06 – 3.87 (m, 2H, R1/R2), 2.84 – 2.71 (m, 2H, R1/R2) ppm.

^{31}P NMR (162 MHz, CD_2Cl_2, rotameric mixture): δ = -6.25 (R1), -6.35 (R2) ppm.

^{13}C-NMR (101 MHz, CD_2Cl_2, rotameric mixture, 2D spectra recorded at 0°C): δ = 166.6 (**C**=O), 162.6 (**C**=O), 158.1, 153.6, 140.4 (R2), 140.4 (R1), 140.3 (R1), 140.2 (R2), 138.0, 135.9 (d, *J*

= 7.1 Hz), 135.8 (d, J = 6.9 Hz), 134.9, 132.9, 131.4, 130.4, 130.1, 129.1, 128.96 (R1), 128.95 (R2), 128.4, 127.9, 127.6, 123.7, 123.4, 122.6, 122.5, 121.9 (R1/R2), 119.9, 119.6 (R1), 119.0 (R2), 118.8, 112.3, 106.2 (R1/R2), 107.0 (R1/R2), 70.7 (d, $^2J_{C\text{-}P}$ = 6.0 Hz, O**C**H_2Ph), 70.6 (d, $^2J_{C\text{-}P}$ = 6.0 Hz, O**C**H_2Ph), 48.2 (R2), 44.9 (R1), 44.7 (R1), 40.6 (R2), 28.7 (R1), 27.4 (R2) ppm.
HRMS (ESI, pos): *m/z* $[M+H]^+$ calculated for $[C_{59}H_{52}N_3O_{11}P_2]^+$: 1040.3072, found: 1040.3068.
IR (KBr): $\tilde{\nu}$ = 3433 (br), 3033 (w), 1617 (br), 1507 (s), 1489 (s), 1455 (m), 1407 (w), 1310 (m), 1280 (s), 1222 (s), 1017 (s), 958 (m), 870 (w), 745 (m), 696 (s) cm^{-1}.
UV / Vis (CH_2Cl_2): λ_{max} = 308, 257 nm.

2-(5-((4-Phenoxyphenyl)carbamoyl)-1*H*-indole-2-carbonyl)-1,2,3,4-tetrahydroisoquinoline-6,7-diyl bis(dihydrogen phosphate) (14)

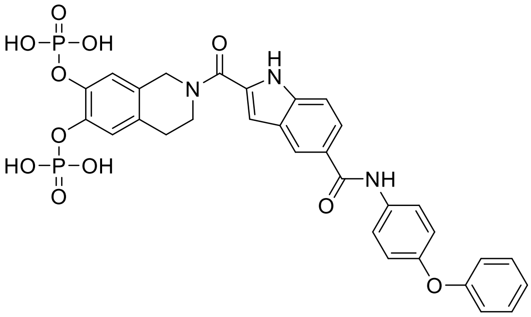

2-(5-((4-Phenoxyphenyl)carbamoyl)-1*H*-indole-2-carbonyl)-1,2,3,4-tetrahydroisoquinoline-6,7-diyl bis(dihydrogen phosphate) **14** was synthesized from **14e** (32 mg, 0.029 mmol, 1.0 eq.) according to **Method 6**. The crude product was purified by reversed-phase column chromatography (100 % H_2O). Lyophilization yielded **14** as a colorless solid (11 mg, 55 %).

^{1}H-NMR (400 MHz, DMSO-d$_6$ / MeOD, rotameric mixture, recorded at 5°C): δ = 8.33 (s, 1H), 7.85 – 7.76 (m, 3H), 7.52 (d, J = 8.6 Hz, 1H), 7.36 (t, J = 7.9 Hz, 2H), 7.09 (t, J = 7.4 Hz, 1H), 7.07 – 7.03 (m, 3H), 7.03 – 6.99 (m, 2H), 6.97 (d, J = 8.0 Hz, 2H), 4.98 (s, 1H, R2), 4.66 (s, 1H, R1), 4.02 (s, 1H, R1), 3.85 (s, 1H, R2), 2.94 – 2.84 (m, 1H, R1), 2.84 – 2.72 (m, 1H, R2) ppm.
^{31}P NMR (162 MHz, D_2O, rotameric mixture): δ = -2.13 ppm.
^{13}C-NMR (101 MHz, DMSO-d$_6$ / MeOD, rotameric mixture, recorded at 5°C): δ = 166.0, 162.1, 157.1, 151.5 , 143.8 (R1), 143.6 (R2), 137.1, 135.0, 131.1, 129.7, 128.4 (R1/R2), 126.9 (R1/R2), 126.2, 125.9, 122.8, 122.7, 121.7, 121.6, 121.5, 119.1, 117.5, 111.5, 105.5 (R2), 104.7 (R1), 47.9 (R2), 44.4 (R1), 44.0 (R1), 40.1 (R2), 27.9 (R1), 26.7 (R2) ppm.
HRMS (ESI, pos): *m/z* $[M-H]^-$ calculated for $[C_{31}H_{26}N_3O_{11}P_2]^-$: 678.1048, found: 678.1071.

IR (KBr): $\tilde{\nu}$ = 3441 (br), 3201 (w), 1635 (m), 1616 (m), 1508 (m), 1488 (w), 1444 (w), 1404 (w), 1320 (w), 1224 (m), 1190 (w), 1088 (w), 920 (w), 838 (w), 754 (w) cm^{-1}.
UV / Vis (H_2O): λ_{max} = 308, 260, 202 nm.
mp: 159°C (decomposition).

2-Methyl-*N*-(4-phenoxyphenyl)quinoline-6-carboxamide (15b)

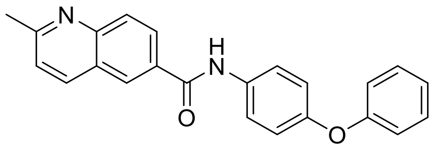

2-Methyl-*N*-(4-phenoxyphenyl)quinoline-6-carboxamide **15b** was synthesized from 2-methyl-6-quinoline carboxylic acid **15a** (200 mg, 1.07 mmol, 1.0 eq.) and 4-phenoxyaniline (198 mg, 1.07 mmol, 1.0 eq.) according to **Method 7A**, using NEt_3 (635 µL, 3.21 mmol, 3.0 eq.). Purification of the crude product by column chromatography (7:3 hexane / EtOAc, *v/v*) yielded **15b** as a colorless solid (275 mg, 73 %).

R_f = 0.23 (7:3 hexane / EtOAc, *v/v*).
^{1}H-NMR (400 MHz, DMSO-d_6): δ = 10.49 (s, 1H, -N**H**), 8.56 (d, *J* = 2.1 Hz, 1H), 8.40 (d, *J* = 8.4 Hz, 1H), 8.21 (dd, *J* = 8.7, 2.1 Hz, 1H), 8.03 (d, *J* = 8.8 Hz, 1H), 7.86 – 7.81 (m, 2H), 7.52 (d, *J* = 8.4 Hz, 1H), 7.42 – 7.35 (m, 2H), 7.15 – 7.09 (m, 1H), 7.08 – 7.04 (m, 2H), 7.03 – 6.98 (m, 2H), 2.70 (s, 3H) ppm.
^{13}C-NMR (76 MHz, DMSO-d_6): δ = 165.1, 160.9, 157.3, 152.2, 148.5, 137.2, 135.0, 131.9, 130.0, 128.4, 128.1, 128.0, 125.4, 123.10, 123.05, 122.1, 119.3, 118.0, 25.1 ppm.
HRMS (ESI, pos): *m/z* $[M+H]^+$ calculated for $[C_{23}H_{19}N_2O_2]^+$: 355.1441, found: 355.1449.
IR (KBr): $\tilde{\nu}$ = 3434 (m), 3056 (w), 1648 (s), 1600 (m), 1533 (s), 1507 (s), 1489 (s), 1408 (m), 1227 (s), 1166 (m), 870 (w), 847 (w), 748 (w), 691 (w) cm^{-1}.
UV / Vis (MeOH): λ_{max} = 323, 238, 212 nm.
mp: 178 - 180°C.

6-((4-Phenoxyphenyl)carbamoyl)quinoline-2-carboxylic acid (15c)

6-((4-Phenoxyphenyl)carbamoyl)quinoline-2-carboxylic acid **15c** was prepared essentially as described by *Jerchel et al*.[19]

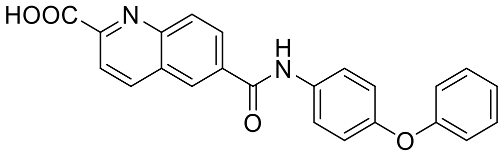

In brief, to a solution of 2-methyl-*N*-(4-phenoxyphenyl)quinoline-6-carboxamide **15b** (175 mg, 0.494 mg, 1.0 eq.) in pyridine (1 mL) was added SeO_2 (220 mg, 1.98 mmol, 4.0 eq.). The mixture was stirred at 115 °C for 20 h, filtered and concentrated under reduced pressure to yield **15c** without further purification as an orange solid (159 mg, 84 %).

$\mathbf{R_f}$ = 0.37 (5 % CH_3COOH, 10 % MeOH in DCM, *v/v*).
^{1}H-NMR (400 MHz, DMSO-d_6): δ = 10.61 (s, 1H, -N**H**), 8.71 (d, *J* = 8.5 Hz, 1H), 8.70 (d, *J* = 2.0 Hz, 1H), 8.33 (dd, *J* = 8.8, 2.0 Hz, 1H), 8.27 (d, *J* = 8.8 Hz, 1H), 8.19 (d, *J* = 8.5 Hz, 1H), 7.86 – 7.81 (m, 2H), 7.41 – 7.36 (m, 2H), 7.15 – 7.10 (m, 1H), 7.09 – 7.05 (m, 2H), 7.03 – 6.99 (m, 2H) ppm.
^{13}C-NMR (101 MHz, DMSO-d_6): δ = 166.2, 164.8, 157.3, 152.4, 150.2, 147.8, 138.9, 134.9, 134.5, 130.05, 129.97, 129.0, 128.2, 128.1, 123.2, 122.2, 121.5, 119.3, 118.1 ppm.
HRMS (ESI, pos): *m/z* $[M+H]^+$ calculated for $[C_{23}H_{17}N_2O_4]^+$: 385.1183, found: 385.1177.
IR (KBr): $\tilde{\nu}$ = 3427 (s), 3060 (w), 1651 (m), 1538 (w), 1507 (s), 1489 (m), 1410 (w), 1226 (m), 1167 (w), 872 (w), 845 (w), 768 (w), 692 (w) cm^{-1}.
UV / Vis (MeOH): λ_{max} = 323, 240, 212 nm.
mp: 212 - 215°C.

2-(6,7-Dihydroxy-1,2,3,4-tetrahydroisoquinoline-2-carbonyl)-*N*-(4-phenoxyphenyl) quinoline-6-carboxamide (15d)

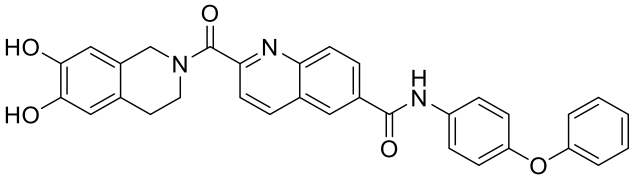

2-(6,7-Dihydroxy-1,2,3,4-tetrahydroisoquinoline-2-carbonyl)-*N*-(4-phenoxyphenyl) quinoline-6-carboxamide **15d** was prepared from 6-((4-phenoxyphenyl)carbamoyl)quinoline-2-carboxylic acid **15c** (156 mg, 0.407 mmol, 1.0 eq.) and **9b** (100 mg, 0.407 mmol, 1.0 eq.) following **Method 7B**. The crude product was purified by column chromatography (0 - 5 % MeOH in DCM, *v/v*) to yield **15d** as an orange solid (152 mg, 70 %).

R_f = 0.28 (5 % MeOH in DCM, *v/v*).

^{1}H-NMR (400 MHz, CD_3OD, rotameric mixture): δ = 8.62 – 8.60 (m, 1H), 8.60 – 8.58 (m, 1H), 8.34 – 8.29 (m, 1H), 8.20 – 8.15 (m, 1H), 7.78 (d, *J* = 8.4 Hz, 1H), 7.76 – 7.71 (m, 2H), 7.39 – 7.32 (m, 2H), 7.15 – 7.08 (m, 1H), 7.06 – 6.97 (m, 4H), 6.67 – 6.30 (m, 2H, R1/R2), 4.79 (s, 1H, R1), 4.55 (s, 1H, R2), 3.99 (t, *J* = 6.0 Hz, 1H, R2), 3.69 (t, *J* = 5.8 Hz, 1H, R1), 2.87 (t, *J* = 6.1 Hz, 1H, R2), 2.81 (t, *J* = 5.8 Hz, 1H, R1) ppm.

^{13}C-NMR (101 MHz, CD_3OD, rotameric mixture): δ = 167.5 (R1, **C**=O), 167.3 (R2, **C**=O), 165.5, 156.8, 154.8, 153.3, 147.0, 143.5 (R2), 143.34 (R1), 143.27 (R1), 143.1 (R2), 138.3 (R2), 138.2 (R1), 133.32 (R2), 133.29 (R1), 133.2, 128.8, 128.40 (R1), 128.35 (R2), 127.90 (R2), 127.86 (R1), 127.1, 126.8, 124.5 (R2), 124.3 (R1), 122.6 (R2), 122.18, 122.17 (R1), 121.9, 119.9, 118.1, 117.5, 114.1 (R1/R2), 111.9 (R1), 111.4 (R2), 47.7 (R2), 44.4 (R1), 43.3 (R1), 40.2 (R2), 27.5 (R1), 26.4 (R2) ppm.

HRMS (ESI, pos): *m/z* $[M+H]^+$ calculated for $[C_{32}H_{26}N_3O_5]^+$: 532.1867, found: 532.1869.

IR (KBr): $\tilde{\nu}$ = 3415 (br), 2925 (w), 1637 (s), 1618 (s), 1529 (m), 1507 (s), 1489 (s), 1449 (m), 1283 (w), 1227 (m), 1166 (w), 865 (w), 843 (w), 692 (w), 624 (w) cm^{-1}.

UV / Vis (MeOH): λ_{max} = 329, 238, 206 nm.

mp: 240°C (decomposition).

Tetrabenzyl (2-(6-((4-phenoxyphenyl)carbamoyl)quinoline-2-carbonyl)-1,2,3,4-tetrahydroisoquinoline-6,7-diyl) bis(phosphate) (15e)

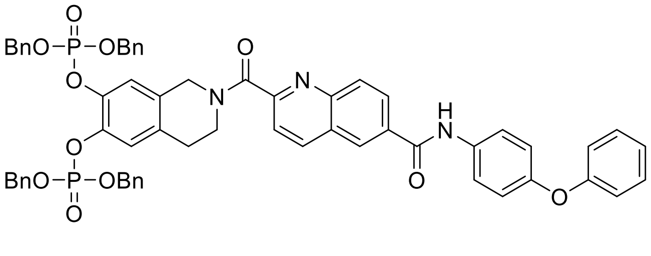

Tetrabenzyl (2-(6-((4-phenoxyphenyl)carbamoyl)quinoline-2-carbonyl)-1,2,3,4-tetrahydroisoquinoline-6,7-diyl) bis(phosphate) **15e** was synthesized from **15d** (120 mg, 0.226 mmol, 1.0 eq.) according to **Method 5**. Instead of CH_3CN dry DMF was used as solvent. The crude product was purified by column chromatography (8:2 → 9:1 EtOAc / hexane) to yield **15e** as a colorless oil (41 mg, 17 %).

R_f = 0.33 (9:1 EtOAc / hexane, *v/v*).

^{1}H-NMR (400 MHz, $CDCl_3$, rotameric mixture): δ = 9.31 – 9.19 (m, 1H, R1/R2, N**H**), 8.45 – 8.44 (m, 1H, R1/R2), 8.31 – 8.27 (m, 1H, R1/R2), 8.21 (d, *J* = 8.3 Hz, 1H), 8.15 – 8.12 (m, 1H, R1/R2), 7.92 – 7.87 (m, 2H, R1/R2), 7.71 – 7.69 (m, 1H, R1/R2), 7.48 – 7.42 (m, 2H), 7.42 –

7.26 (m, 20H), 7.23 – 7.18 (m, 1H), 7.18 – 6.85 (m, 6H, R1/R2), 5.22 (d, $^{3}J_{H-P}$ = 8.3 Hz, 2H, OC**H**$_2$Ph, R1), 5.20 (d, $^{3}J_{H-P}$ = 8.3 Hz, 2H, OC**H**$_2$Ph, R1), 5.18 (d, $^{3}J_{H-P}$ = 8.4 Hz, 2H, OC**H**$_2$Ph, R2), 5.10 (d, $^{3}J_{H-P}$ = 8.3 Hz, 2H, OC**H**$_2$Ph, R2), 4.89 (s, 1H, R1), 4.73 (s, 1H, R2), 4.09 (t, *J* = 6.0 Hz, 1H, R2), 3.84 (t, *J* = 5.8 Hz, 1H, R1), 2.97 – 2.89 (m, 2H, R1/R2) ppm.
^{31}P NMR (162 MHz, $CDCl_3$, rotameric mixture): δ = -6.04 (R1), -6.10 (R2), -6.20 (R1), -6.29 (R2) ppm.
^{13}C-NMR (101 MHz, $CDCl_3$, rotameric mixture): δ = 167.9 (R1, **C**=O), 167.7 (R2, **C**=O), 165.2 (**C**=O), 157.6, 154.8 (R1), 154.7 (R2), 153.8, 147.62 (R1), 147.59 (R2), 140.1 – 139.7 (m, R1/R2), 138.2, 135.5 – 134.1 (m), 134.3 (R2), 134.2 (R1), 134.0, 132.3 (R2), 132.0 (R1), 130.5 (R2), 130.3 (R1), 130.2, 129.9, 128.9 – 128.7 (m, R1/R2), 128.7 – 128.6 (m), 128.5 (R2), 128.4 (R1), 128.2 – 128.0 (m), 127.73 (R1), 127.69 (R2), 127.34 (R2), 127.27 (R1), 123.2, 122.32 (R1), 122.29 (R2), 121.8 (R2), 121.7 (R1), 121.5 (R2), 121.2 (R1), 119.68 (R2), 119.65 (R1), 119.0 (R1/R2), 118.6, 70.5 – 70.2 (m, O**C**H$_2$Ph, R1/R2), 48.6 (R2), 44.7 (R1), 44.4 (R1), 40.5 (R2), 29.0 (R1), 27.8 (R2) ppm.
HRMS (ESI, pos): *m/z* $[M+H]^+$ calculated for $[C_{60}H_{52}N_3O_{11}P_2]^+$: 1052.3072, found: 1052.3072.
IR (KBr): $\tilde{\nu}$ = 3416 (br), 3062 (w), 3034 (w), 1666 (s), 1636 (s), 1540 (m), 1506 (s), 1488 (s), 1455 (m), 1320 (m), 1283 (s), 1222 (s), 1193 (m), 1158 (m), 1015 (s), 959 (s), 895 (m), 740 (s), 695 (s) cm^{-1}.
UV / Vis ($CHCl_3$): λ_{max} = 317, 245 nm.

2-(6-((4-Phenoxyphenyl)carbamoyl)quinoline-2-carbonyl)-1,2,3,4-tetrahydroisoquinoline-6,7-diyl bis(dihydrogen phosphate) (15)

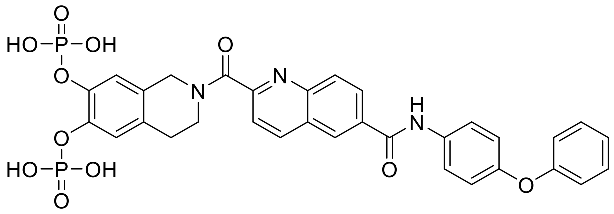

2-(6-((4-Phenoxyphenyl)carbamoyl)quinoline-2-carbonyl)-1,2,3,4-tetrahydroisoquinoline-6,7-diyl bis(dihydrogen phosphate) **15** was prepared from **15e** (59 mg, 0.056 mmol, 1.0 eq.) according to **Method 6**. The crude product was purified by reversed-phase column chromatography (100 % H_2O). Lyophilization yielded **15** as an off-white solid (28 mg, 72 %).

R$_f$ = 0.34 (8:2 H_2O / CH_3CN, *v/v*).
^{1}H-NMR (400 MHz, DMSO-d$_6$, rotameric mixture): δ = 8.69 – 8.62 (m, 2H), 8.32 – 8.27 (m, 1H, R1/R2), 8.19 – 8.16 (m, 1H, R1/R2), 7.86 – 7.72 (m, 3H), 7.38 (t, *J* = 7.9 Hz, 2H), 7.12 (t, *J* =

7.4 Hz, 1H), 7.08 – 6.72 (m, 6H, R1/R2), 4.75 (s, 1H, R1), 4.55 (s, 1H, R2), 3.89 (t, *J* = 6.0 Hz, 1H, R2), 3.63 – 3.59 (m, 1H, R1), 2.87 – 2.76 (m, 2H, R1/R2) ppm.
^{31}P NMR (162 MHz, DMSO-d_6, rotameric mixture): δ = -4.56 (R1), -4.63 (R2) ppm.
^{13}C-NMR (101 MHz, DMSO-d_6, rotameric mixture): δ = 166.9 (**C**=O), 164.8 (**C**=O), 157.3, 155.7, 152.3, 147.2, 144.7 – 144.1 (m), 138.7, 135.0 (R2), 134.9 (R1), 133.6, 130.0, 129.3, 128.8, 128.24, 128.18, 126.9, 126.6, 123.1, 122.14 (R1), 122.08 (R2), 121.8, 121.0, 119.6 (R1), 119.3, 119.1 (R2), 118.0, 47.8 (R2), 44.2 (R1), 43.3 (R1), 39.8 (R2), 27.9 (R1/R2) ppm.
HRMS (ESI, neg): *m/z* $[M-H]^-$ calculated for $[C_{32}H_{26}N_3O_{11}P_2]^-$: 690.1048, found: 690.1050.
IR (KBr): $\tilde{\nu}$ = 3415 (br), 3149 (br), 2797 (w), 1620 (s), 1538 (w), 1507 (s), 1489 (s), 1448 (w), 1401 (s), 1385 (s), 1223 (m), 1103 (m), 980 (w), 919 (m), 839 (m), 691 (w), 623 (w) cm^{-1}.
UV / Vis (H_2O): λ_{max} = 325, 242, 201 nm.
mp: 185°C (decomposition).

Methyl 2-((4-phenoxyphenyl)carbamoyl)-1*H*-indole-5-carboxylate (16b)

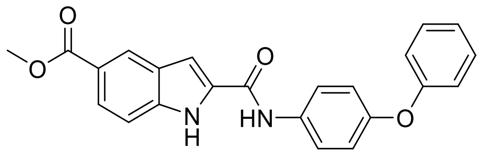

Methyl 2-((4-phenoxyphenyl)carbamoyl)-1*H*-indole-5-carboxylate **16b** was prepared from 5-(methoxycarbonyl)-1*H*-indole-2-carboxylic acid **16a** (100 mg, 0.457 mmol, 1.0 eq.) and 4-phenoxyaniline (85 mg, 0.46 mmol, 1.0 eq.) according to **Method 7A**. After stirring overnight, the crude product was purified by column chromatography (9:1 → 1:1 hexane / EtOAc) yielded **16b** as a colorless solid (115 mg, 65 %).

R_f = 0.23 (7:3 hexane / EtOAc, *v/v*).
^{1}H-NMR (400 MHz, DMSO-d_6): δ = 12.18 – 12.11 (m, 1H, N**H**), 10.39 (s, 1H, N**H**), 8.42 – 8.39 (m, 1H), 7.86 – 7.79 (m, 3H), 7.57 – 7.52 (m, 2H), 7.42 – 7.36 (m, 2H), 7.15 – 7.10 (m, 1H), 7.09 – 7.04 (m, 2H), 7.03 – 6.98 (m, 2H), 3.86 (s, 3H) ppm.
^{13}C-NMR (75 MHz, DMSO-d_6): δ = 167.0, 159.2, 157.3, 152.2, 139.2, 134.6, 133.3, 130.0, 126.7, 124.6, 124.3, 123.1, 122.0, 121.5, 119.4, 118.0, 112.4, 105.1, 51.8 ppm.
HRMS (ESI, pos): *m/z* $[M+H]^+$ calculated for $[C_{23}H_{19}N_2O_4]^+$: 387.1339, found: 387.1339.
IR (KBr): $\tilde{\nu}$ = 3362 (br), 3270 (br), 2949 (w), 1698 (s), 1650 (s), 1617 (s), 1549 (s), 1507 8s), 1489 (s), 1412 (m), 1341 (s), 1268 (s), 1228 (s), 1196 (s), 872 (w), 759 (s), 692 (w) cm^{-1}.
UV / Vis (MeOH): λ_{max} = 320, 257, 206 nm.
mp: 281 - 282°C (decomposition).

2-((4-Phenoxyphenyl)carbamoyl)-1*H*-indole-5-carboxylic acid (16c)

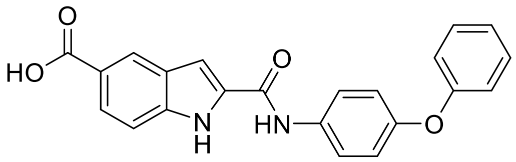

A suspension of **16b** (110 mg, 0.28 mmol, 1.0 eq.) in THF (2.5 mL) and 1 M NaOH (2.5 mL) was stirred at 75 °C overnight. Afterwards, the reaction mixture was acidified by the addition of 1 M HCl and extracted with EtOAc (3 x). The combined organic phases were washed with brine (1 x), dried over Na_2SO_4 and concentrated in vacuo to yield **16c** as an orange solid (102 mg, 96 %).

^{1}H-NMR (400 MHz, DMSO-d_6): δ = 12.57 (s, 1H, OH), 12.13 – 12.06 (m, 1H, NH), 10.37 (s, 1H, NH), 8.37 (d, *J* = 1.6 Hz, 1H), 7.87 – 7.79 (m, 3H), 7.57 – 7.54 (m, 1H), 7.52 (d, *J* = 8.7 Hz, 1H), 7.43 – 7.36 (m, 2H), 7.12 (t, *J* = 7.4 Hz, 1H), 7.09 – 7.04 (m, 2H), 7.01 (d, *J* = 8.0 Hz, 2H) ppm.
^{13}C-NMR (101 MHz, DMSO-d_6): δ = 168.0, 159.2, 157.3, 152.2, 139.0, 134.6, 133.1, 130.0, 126.6, 124.6, 124.6, 123.1, 122.6, 121.9, 119.4, 118.0, 112.2, 105.0 ppm.
HRMS (ESI, neg): *m/z* $[M-H]^-$ calculated for $[C_{22}H_{15}N_2O_4]^-$: 371.1037, found: 371.1024.
IR (KBr): $\tilde{\nu}$ = 3465 (br), 3414 (br), 2925 (w), 1684 (m), 1638 (s), 1617 (s), 1546 (m), 1507 (s), 1489 (s), 1412 (w), 1336 (w), 1227 (s), 1185 (w), 872 (w), 759 (w), 692 (w) cm^{-1}.
UV / Vis (MeOH): λ_{max} = 320, 256, 206 nm.
mp: 274 - 276°C (decomposition).

***N*5-(3,4-dihydroxybenzyl)-*N*2-(4-phenoxyphenyl)-1*H*-indole-2,5-dicarboxamide (16d)**

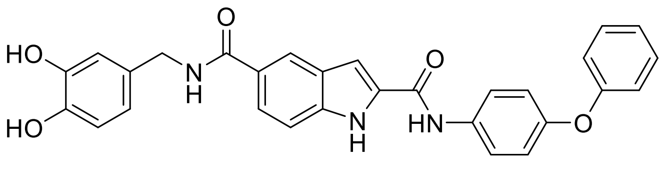

*N*5-(3,4-dihydroxybenzyl)-*N*2-(4-phenoxyphenyl)-1*H*-indole-2,5-dicarboxamide **16d** was prepared from **16c** (100 mg, 0.269 mmol, 1.0 eq.) and 4-(aminomethyl)benzene-1,2-diol (59 mg, 0.27 mmol, 1.0 eq.) according to **Method 7A**. After stirring overnight, the solvent was removed under reduced pressure, and the residue was purified by column chromatography (5 % MeOH in DCM). The product **16d** was isolated as a colorless solid (69 mg, 52 %).

R_f = 0.30 (5 % MeOH in DCM, *v/v*).

^{1}H-NMR (400 MHz, DMSO-d_6): δ = 12.01 – 11.96 (m, 1H, N**H**), 10.32 (s, 1H, N**H**), 8.80 (t, *J* = 5.9 Hz, 1H, N**H**), 8.68 (s, 2H, O**H**), 8.29 (d, *J* = 1.8 Hz, 1H), 7.85 – 7.81 (m, 2H), 7.82 – 7.78 (m, 1H), 7.51 (s, 1H), 7.49 (d, *J* = 8.9 Hz, 1H), 7.42 – 7.35 (m, 2H), 7.16 – 7.09 (m, 1H), 7.09 – 7.04 (m, 2H), 7.03 – 6.99 (m, 2H), 6.76 (d, *J* = 2.1 Hz, 1H), 6.67 (d, *J* = 8.0 Hz, 1H), 6.59 (dd, *J* = 8.1, 2.1 Hz, 1H), 4.34 (d, *J* = 5.9 Hz, 2H) ppm.

^{13}C-NMR (101 MHz, DMSO-d_6): δ = 166.5, 159.2, 157.2, 152.1, 145.0, 144.0, 138.1, 134.6, 132.7, 130.8, 129.9, 126.5, 126.4, 123.2, 123.0, 121.9, 121.5, 119.3, 118.1, 118.0, 115.2, 114.8, 111.8, 104.6, 42.2 ppm.

HRMS (ESI, neg): *m/z* $[M-H]^-$ calculated for $[C_{29}H_{22}N_3O_5]^+$: 492.1565, found: 492.1559.

IR (KBr): ṽ = 3415 (br), 3269 (br), 2926 (w), 1637 (s), 1616 (s), 1532 (s), 1505 (s), 1488 (s), 1411 (w), 1334 (m), 1227 (s), 1195 (m), 1113 (w), 871 (w), 758 (w), 692 (w) cm^{-1}.

UV / Vis (MeOH): λ_{max} = 319, 257, 210 nm.

mp: 224 - 229°C.

Tetrabenzyl (4-((2-((4-phenoxyphenyl)carbamoyl)-1*H*-indole-5-carboxamido)methyl)-1,2-phenylene) bis(phosphate) (16e)

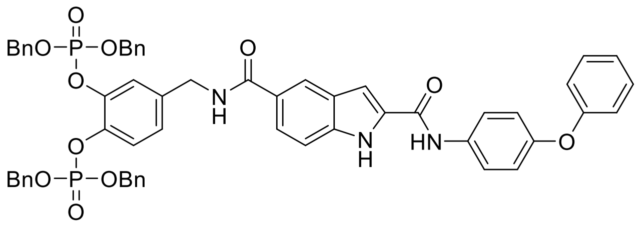

Tetrabenzyl (4-((2-((4-phenoxyphenyl)carbamoyl)-1*H*-indole-5-carboxamido)methyl)-1,2-phenylene) bis(phosphate) **16e** was prepared from **16d** (40 mg, 0.081 mmol, 1.0 eq.) according to **Method 5**. Instead of CH_3CN dry DMF was used as solvent. The crude product was purified by column chromatography (1:1 → 7:3 EtOAc / hexane, *v/v*) to yield **16e** as a colorless solid (23 mg, 30 %).

R_f = 0.21 (7:3 EtOAc / hexane, *v/v*).

^{1}H-NMR (400 MHz, $CDCl_3$): δ = 9.86 – 9.81 (m, 1H, N**H**), 9.07 (s, 1H, N**H**), 8.03 – 7.99 (m, 1H), 7.74 – 7.69 (m, 2H), 7.66 (dd, *J* = 8.7, 1.7 Hz, 1H), 7.40 (t, *J* = 6.0 Hz, 1H, N**H**), 7.34 – 7.29 (m, 3H), 7.25 – 7.12 (m, 22H), 7.11 – 7.05 (m, 2H), 7.04 – 7.00 (m, 1H), 6.99 – 6.96 (m, 2H), 6.95 – 6.92 (m, 2H), 5.09 – 4.97 (m, 8H, OC**H**$_2$Ph), 4.48 (d, *J* = 5.9 Hz, 2H) ppm.

^{31}P NMR (162 MHz, $CDCl_3$): δ = -6.34, -6.67.

^{13}C-NMR (76 MHz, $CDCl_3$): δ = 168.6, 159.6, 157.7, 153.6, 141.53 (t, $^2J_{C\text{-}P}$ = 6.5 Hz), 140.45 (t, $^2J_{C\text{-}P}$ = 6.5 Hz), 138.2, 137.6, 135.3 (d, $^3J_{C\text{-}P}$ = 7.0 Hz), 135.2 (d, $^3J_{C\text{-}P}$ = 7.3 Hz), 133.8, 132.7, 128.9, 128.83, 128.81, 128.73, 128.69, 128.11, 128.10, 127.4, 126.7, 125.0, 123.5, 123.2, 122.4, 122.2, 121.9, 120.8, 119.7, 118.6, 112.0, 105.0, 70.5 (d, $^2J_{C\text{-}P}$ = 6.0 Hz, O**C**H_2Ph), 70.4 (d, $^2J_{C\text{-}P}$ = 5.9 Hz, O**C**H_2Ph), 43.0 ppm.
HRMS (ESI, pos): *m/z* $[M+H]^+$ calculated for $[C_{57}H_{50}N_3O_{11}P_2]^+$: 1014.2915, found: 1014.2915.
IR (KBr): ṽ = 3415 (br), 2922 (w), 1638 (s), 1615 (s), 1540 (s), 1506 (s), 1489 (s), 1456 (m), 1410 (w), 1313 (m), 1278 (s), 1227 (s), 1015 (s), 966 (s), 871 (w), 742 (m), 696 (s) cm^{-1}.
UV / Vis ($CHCl_3$): λ_{max} = 320, 256 nm.
mp: 166 - 168°C.

4-((2-((4-Phenoxyphenyl)carbamoyl)-1*H*-indole-5-carboxamido)methyl)-1,2-phenylene bis(dihydrogen phosphate) (16)

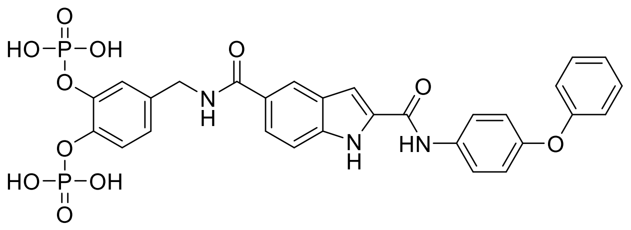

4-((2-((4-Phenoxyphenyl)carbamoyl)-1*H*-indole-5-carboxamido)methyl)-1,2-phenylene bis (dihydrogen phosphate) **16** was prepared from **16e** (23 mg, 0.023 mmol, 1.0 eq.) according to **Method 6**. The crude product was redissolved in CH_3CN, washed with pentane (2 x), dried and purified by reversed-phase column chromatography (100 % H_2O). Lyophilization yielded **16** as a colorless solid (13 mg, 87 %).

R_f = 0.54 (3:2 H_2O / CH_3CN, *v/v*).
^{1}H-NMR (400 MHz, 1:1 D_2O / CH_3CN): δ = 8.82 (d, *J* = 1.7 Hz, 1H), 8.33 (dd, *J* = 8.7, 1.8 Hz, 1H), 8.28 – 8.23 (m, 2H), 8.16 (d, *J* = 8.7 Hz, 1H), 8.00 – 7.94 (m, 3H), 7.89 (s, 1H), 7.83 (d, *J* = 8.4 Hz, 1H), 7.76 – 7.71 (m, 1H), 7.67 – 7.59 (m, 5H), 5.08 (s, 2H) ppm.
^{31}P NMR (162 MHz, 1:1 D_2O / CH_3CN) δ -2.54.
^{13}C-NMR (101 MHz, 1:1 D_2O / CH_3CN): δ = 172.6, 163.2, 160.2, 156.4, 146.8, 146.0, 141.4, 137.4, 136.4, 135.2, 132.9, 129.9, 129.1, 126.4, 126.3, 126.0, 125.7, 125.2, 125.1, 124.6, 122.2, 121.4, 115.2, 108.3, 45.8 ppm.
HRMS (ESI, neg): *m/z* $[M-H]^-$ calculated for $[C_{29}H_{24}N_3O_{11}P_2]^-$: 652.0892, found: 652.0907.
IR (KBr): ṽ = 3425 (br), 3185 (br), 1645 (m), 1609 (m), 1537 (m), 1506 (s), 1489 (m), 1401 (s), 1385 (s), 1334 (w), 1229 (m), 1104 (w), 912 (w), 754 (w) cm^{-1}.

UV / Vis (H_2O): λ_{max} = 316, 258, 200 nm.

mp: 161°C (decomposition).

NMR spectra

^1H-NMR of compound **1a**

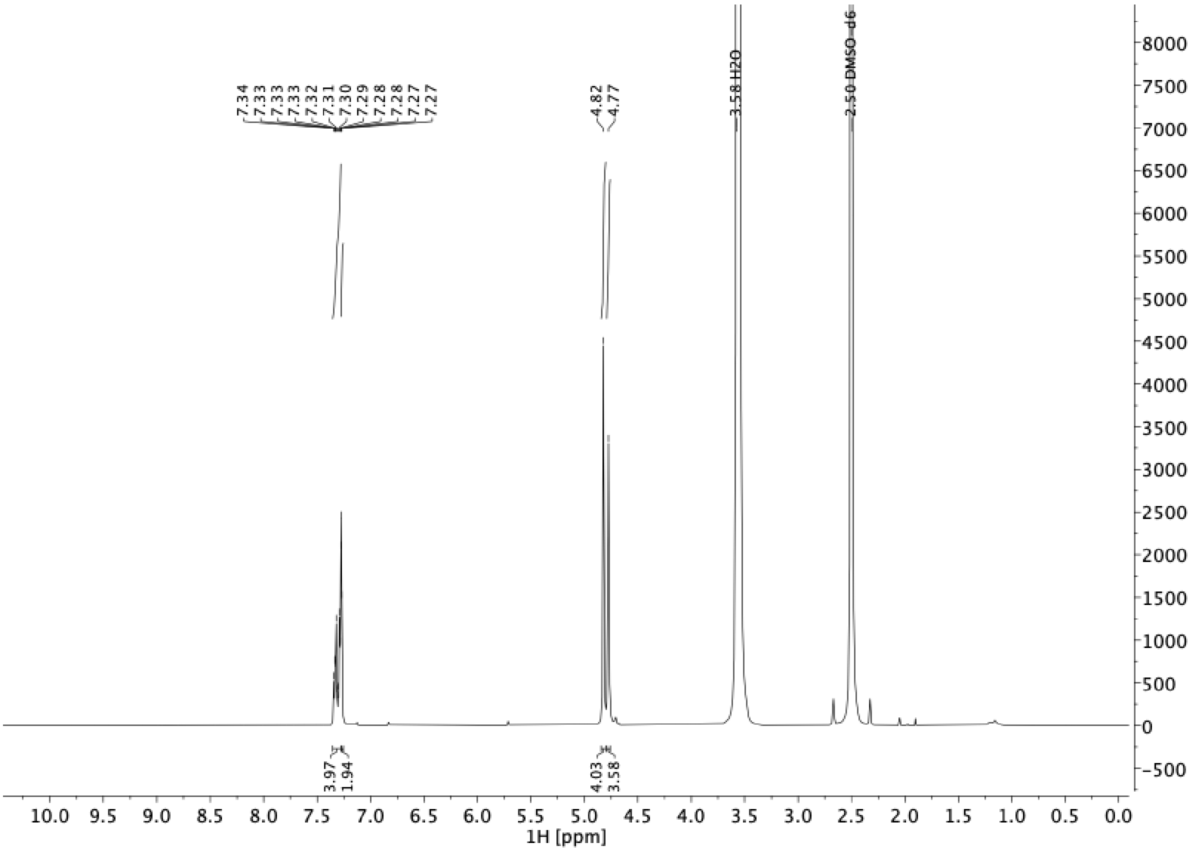

^{31}P-NMR of compound **1a**

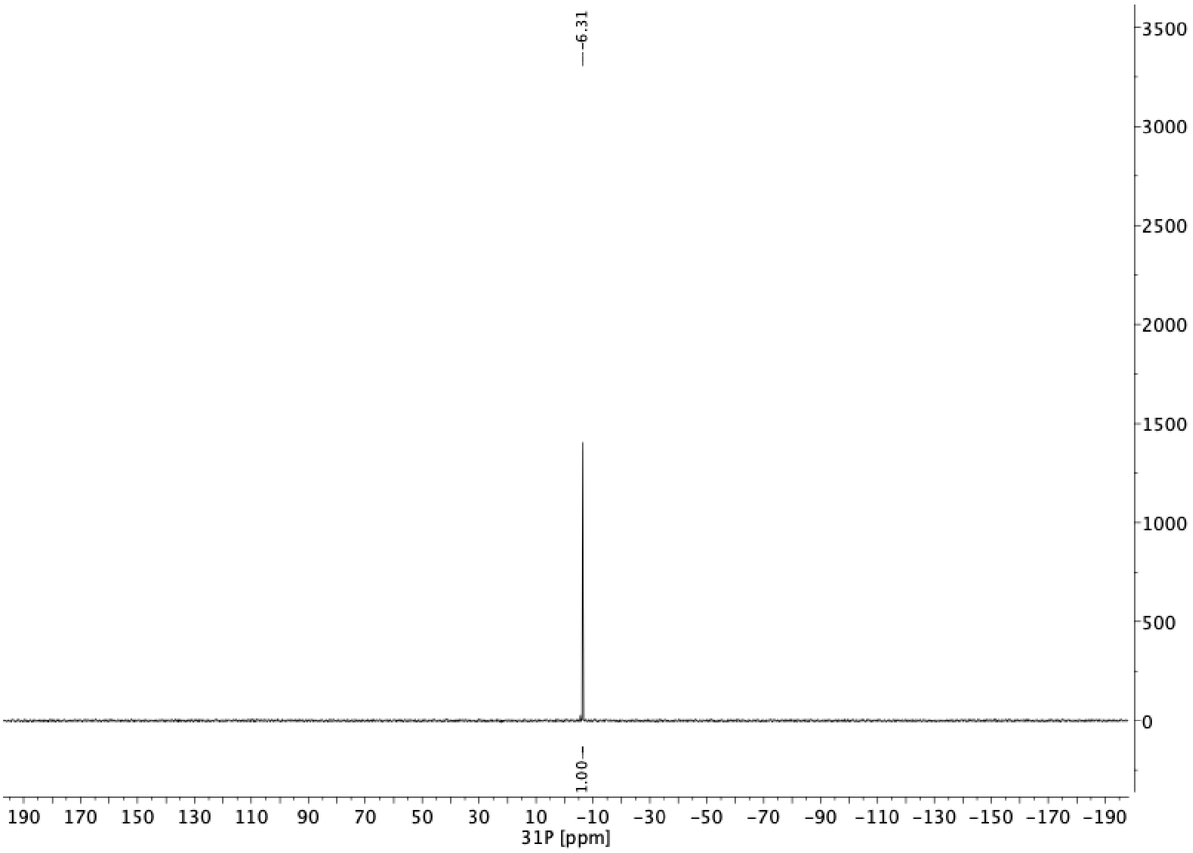

^{13}C-NMR of compound **1a**

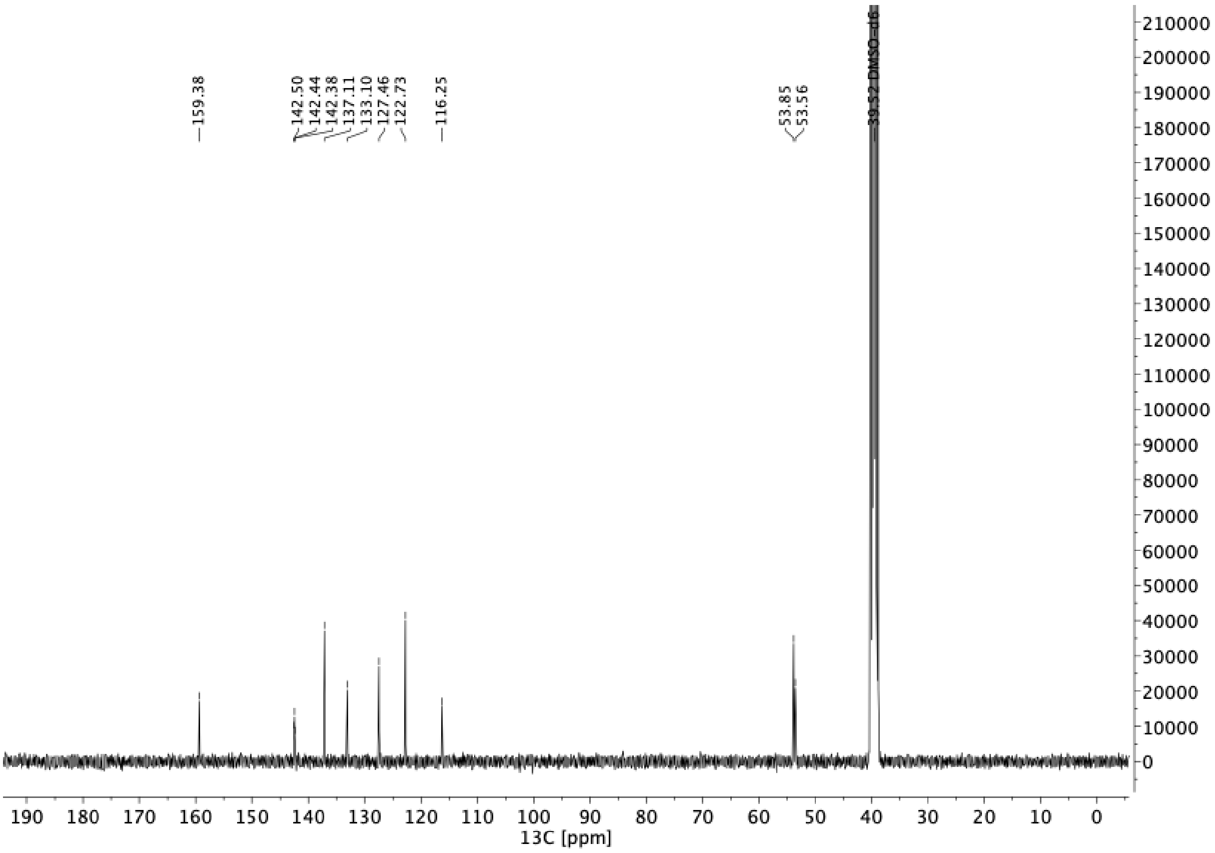

^{1}H-NMR of compound **1b**

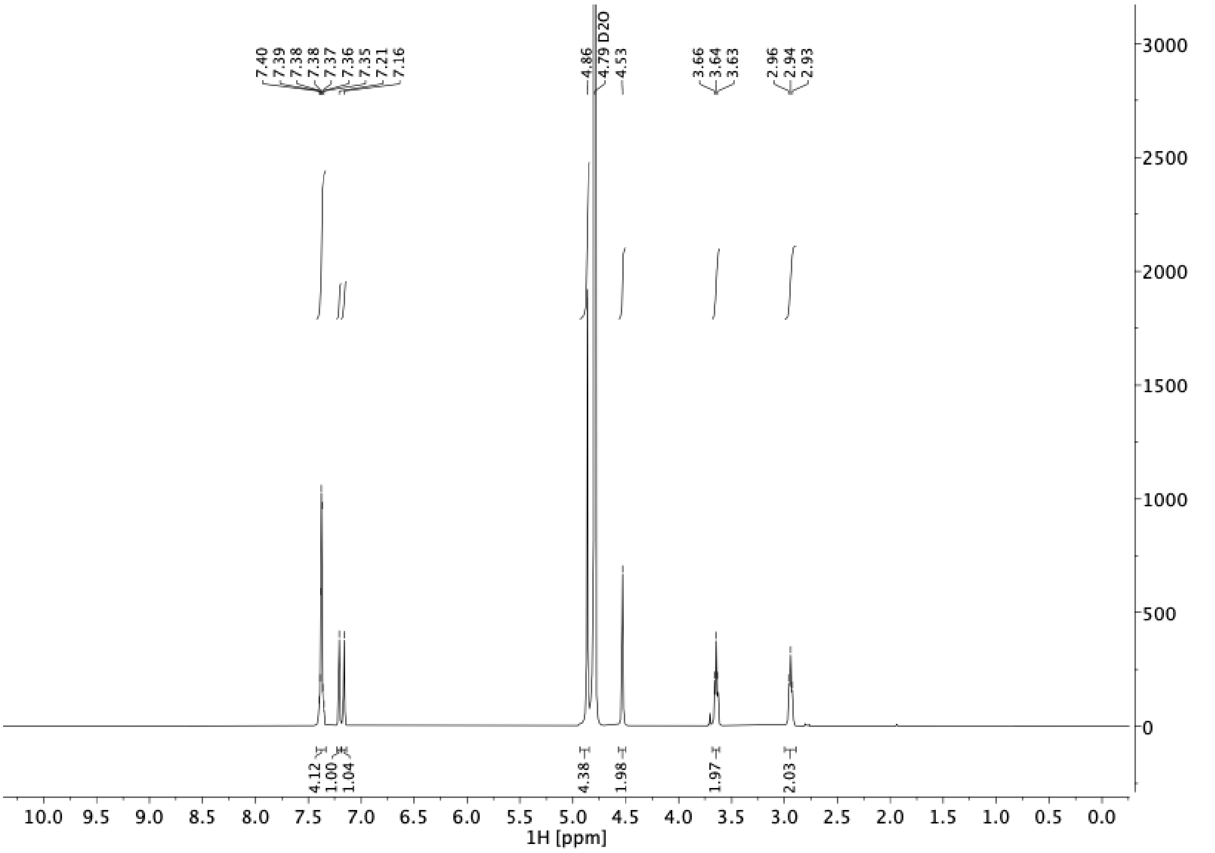

^{31}P-NMR of compound **1b**

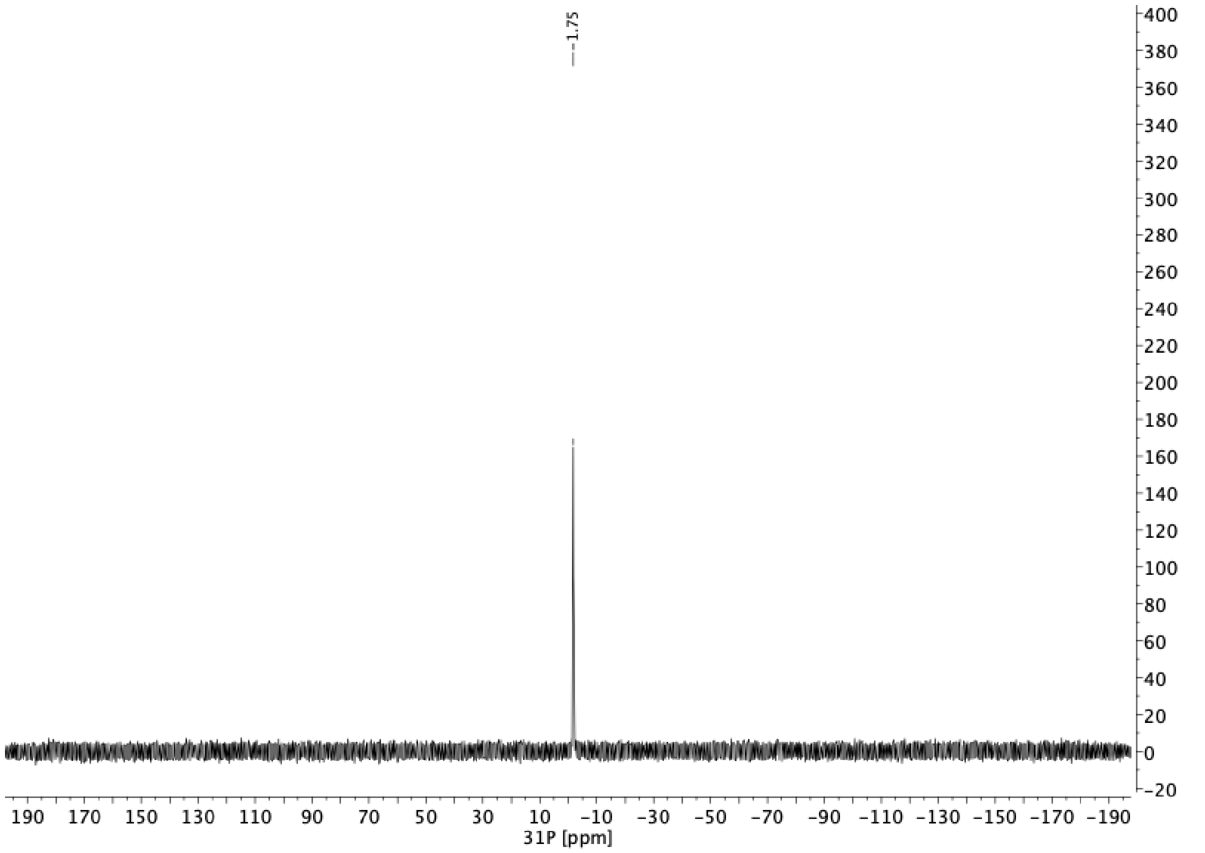

^{13}C-NMR of compound **1b**

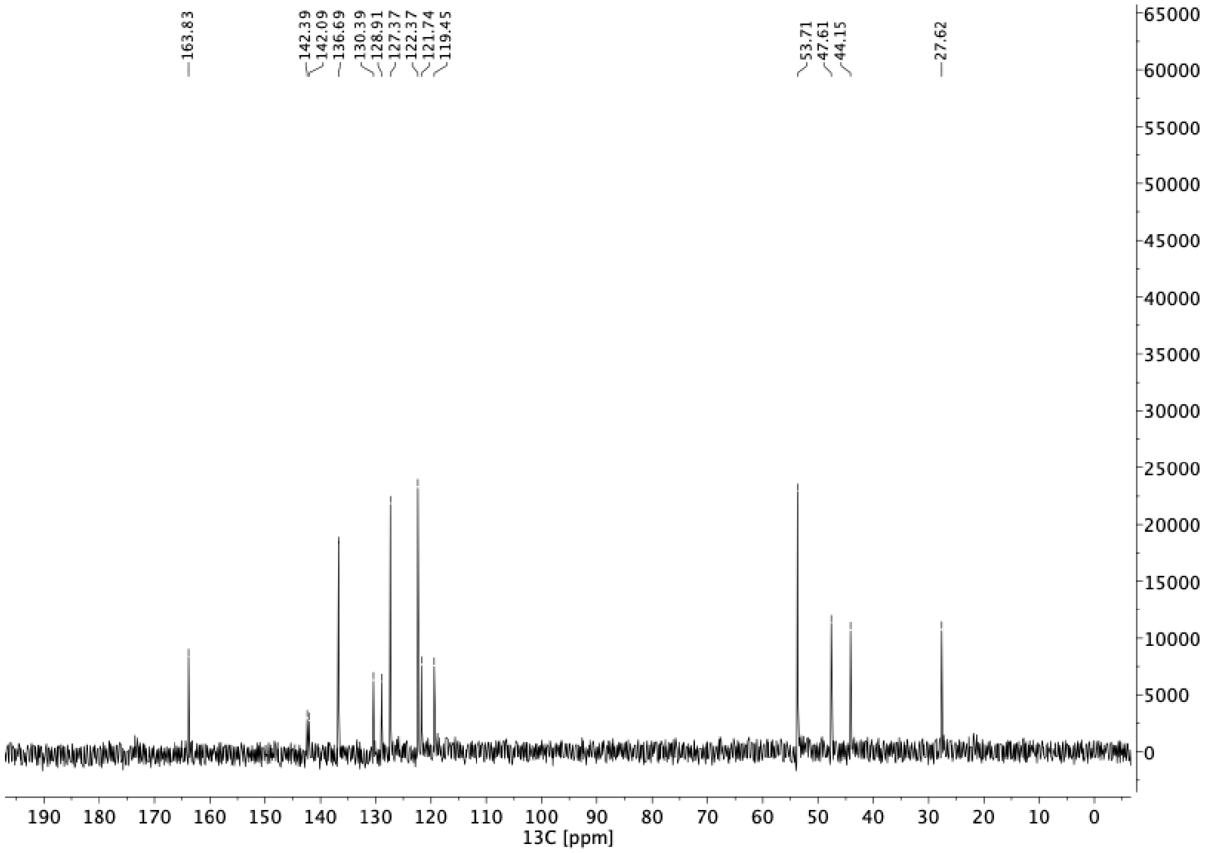

^{1}H-NMR of compound **1c**

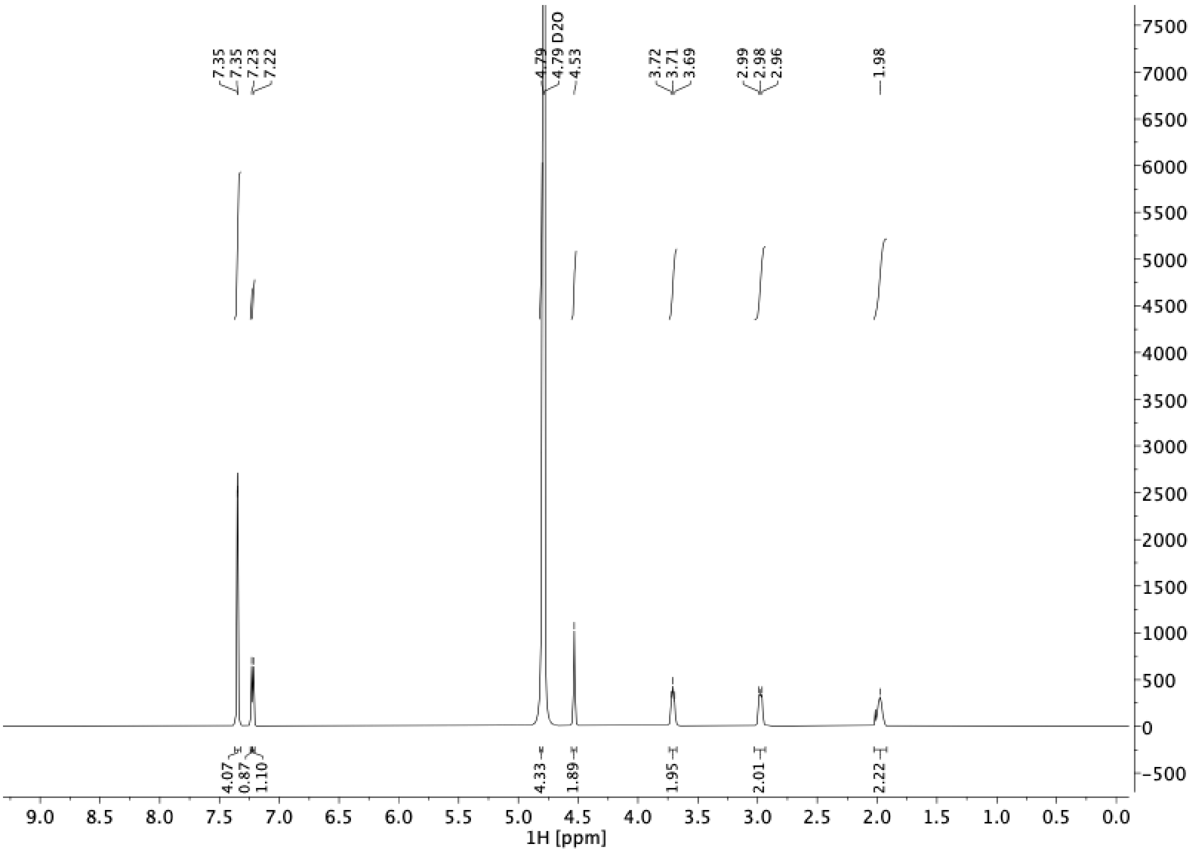

^{31}P-NMR of compound **1c**

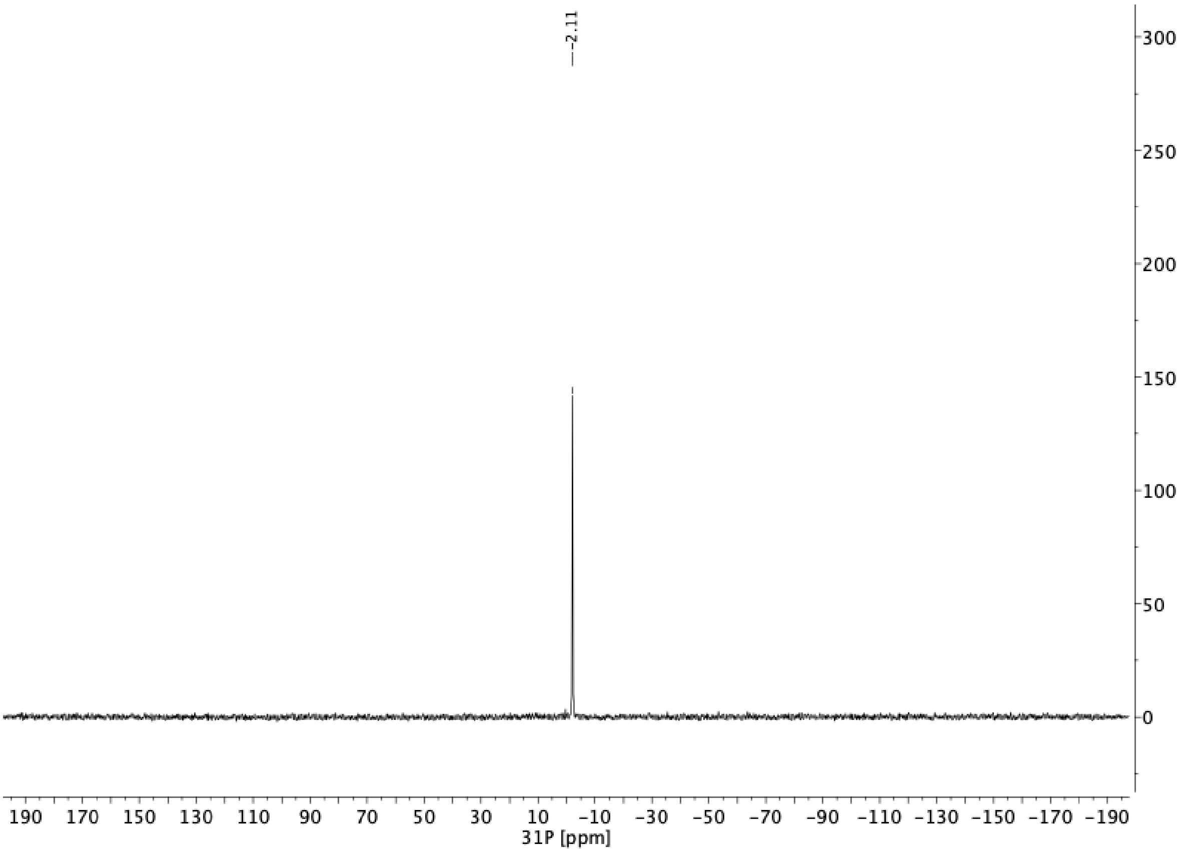

^{13}C-NMR of compound **1c**

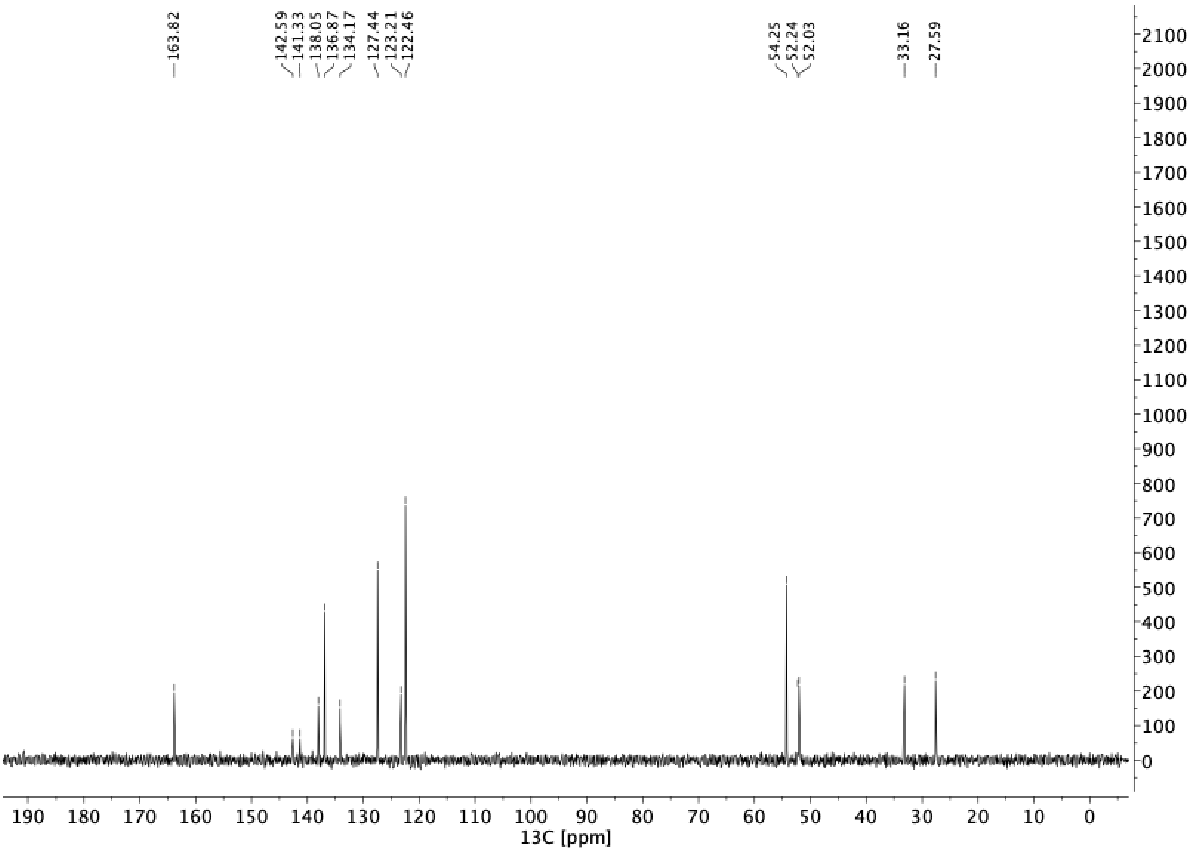

^{1}H-NMR of compound **1d**

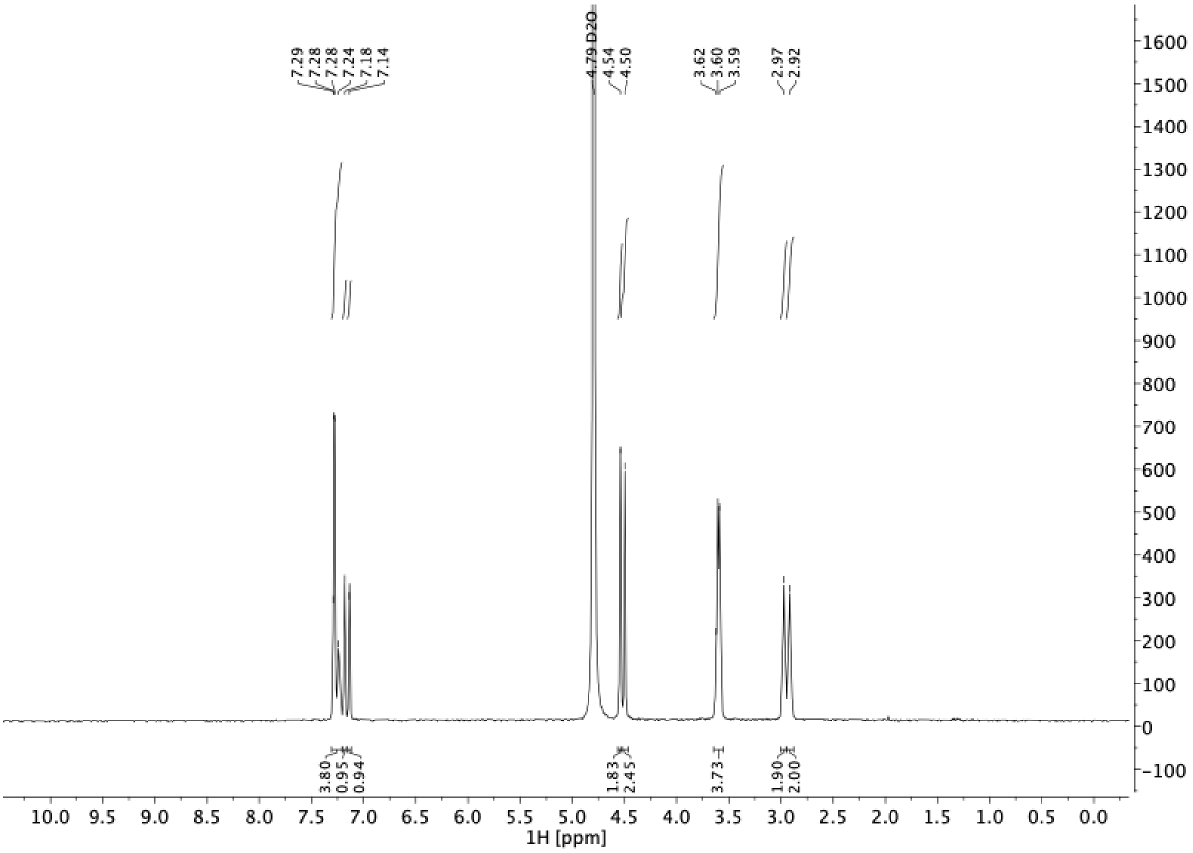

^{31}P-NMR of compound **1d**

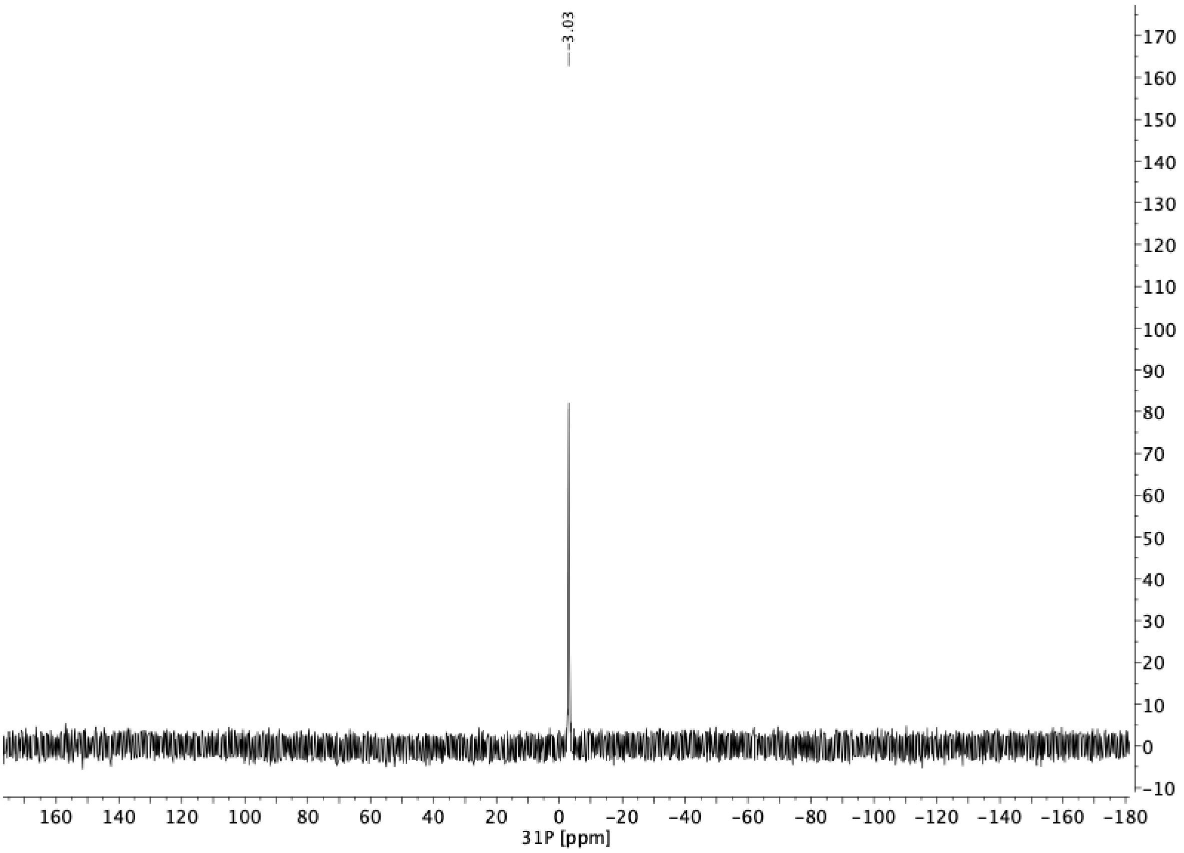

^{13}C-NMR of compound **1d**

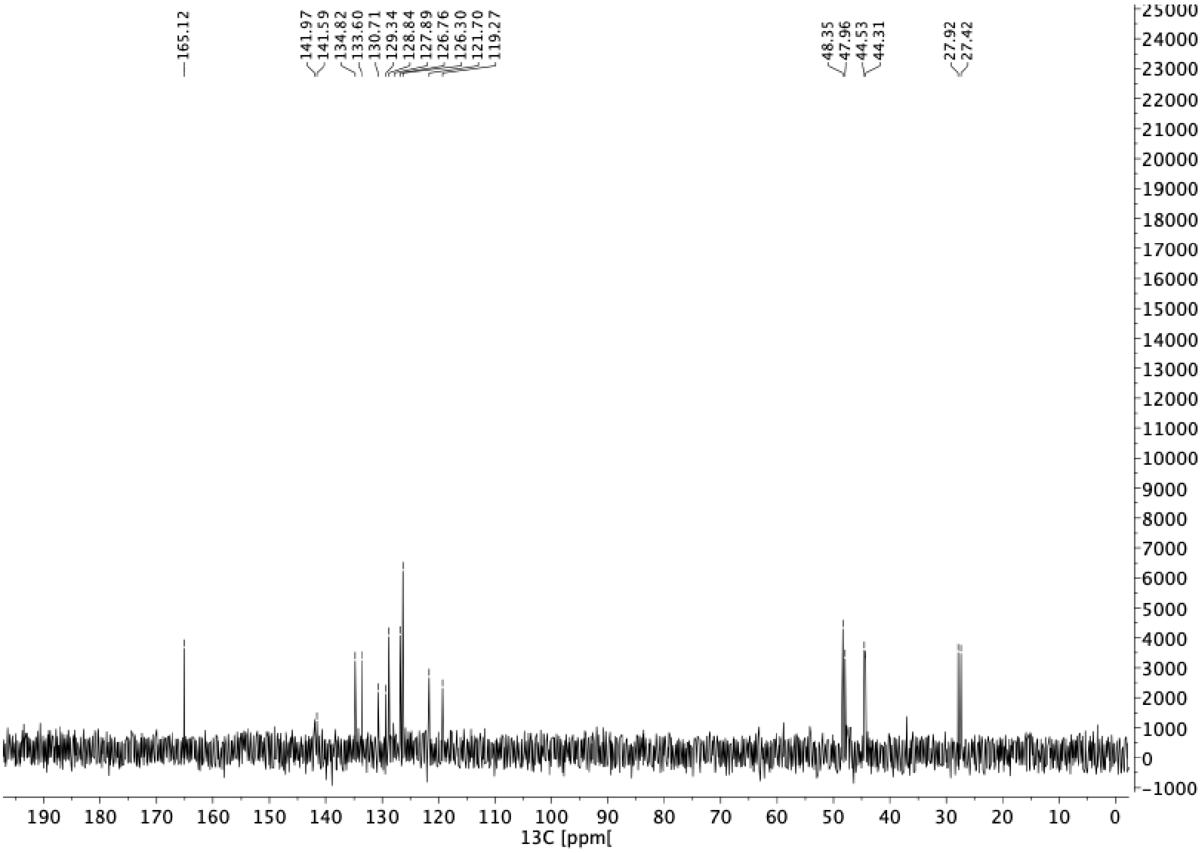

^{1}H-NMR of compound **8a**

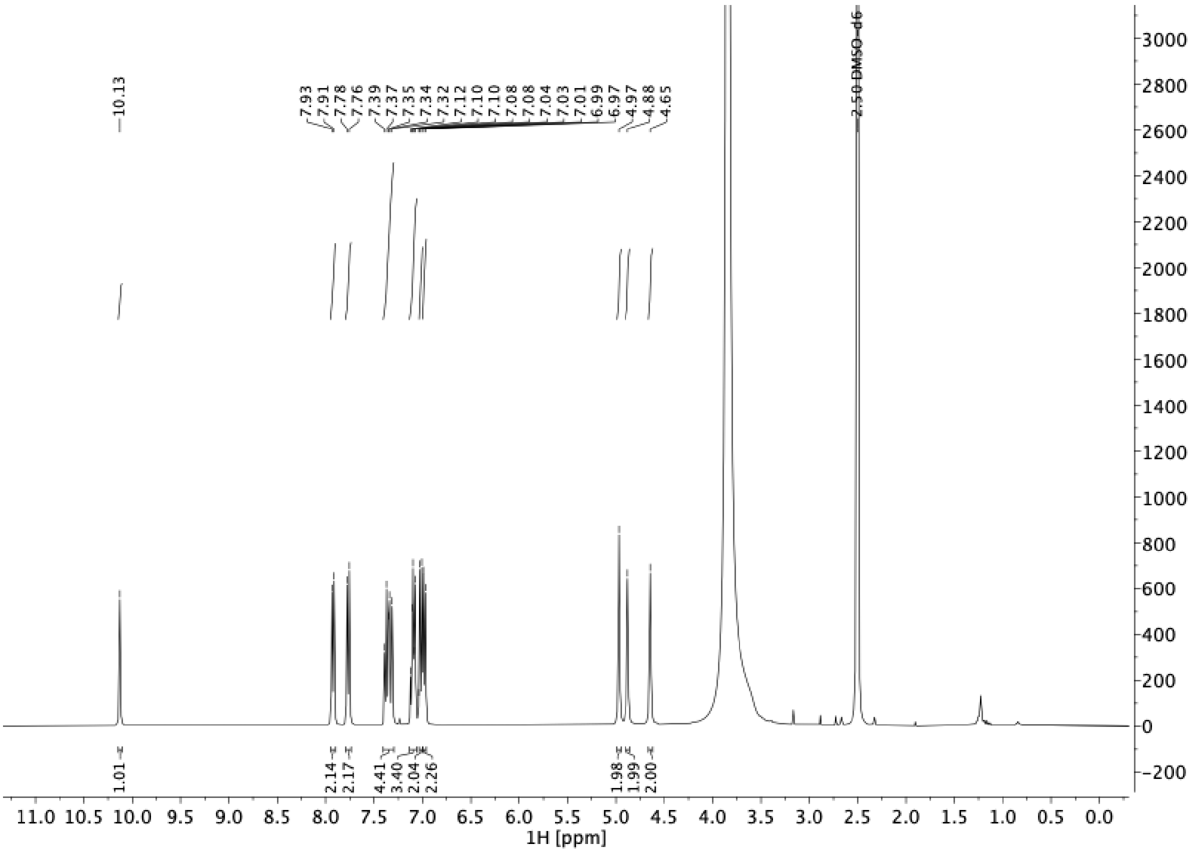

^{31}P-NMR of compound **8a**

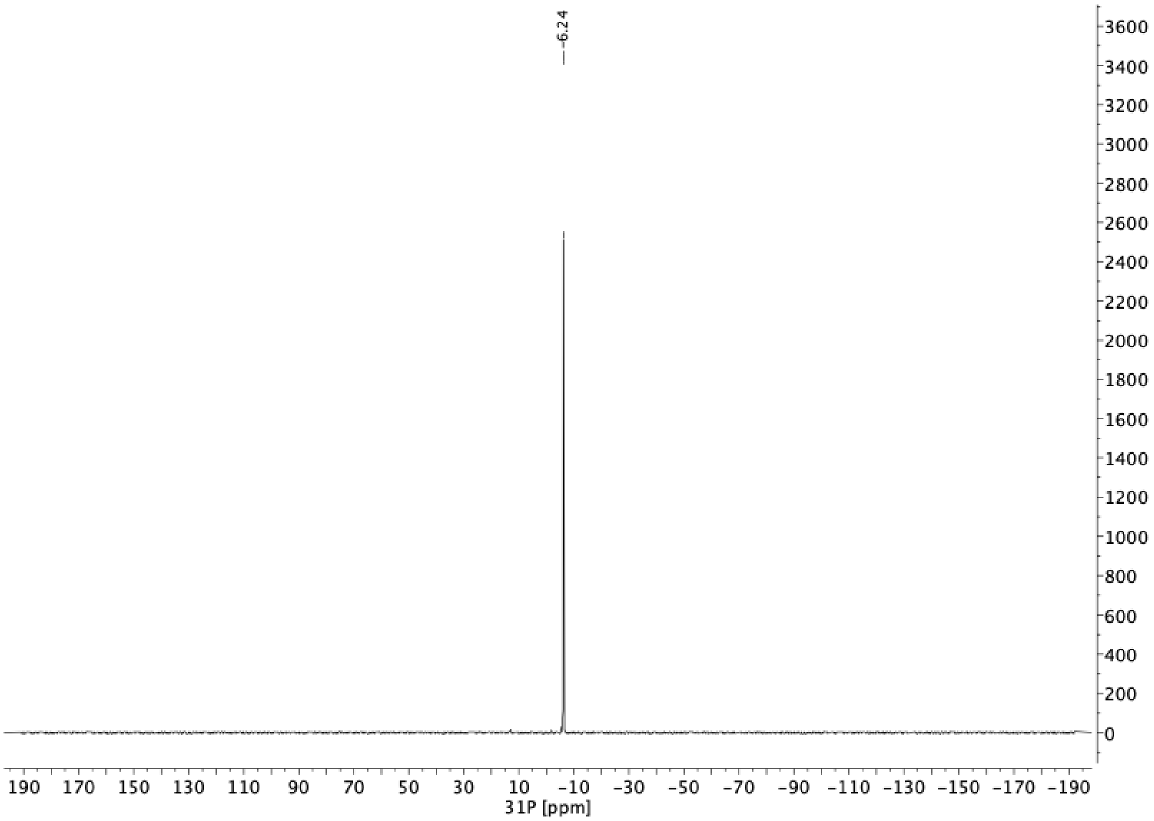

^{13}C-NMR of compound **8a**

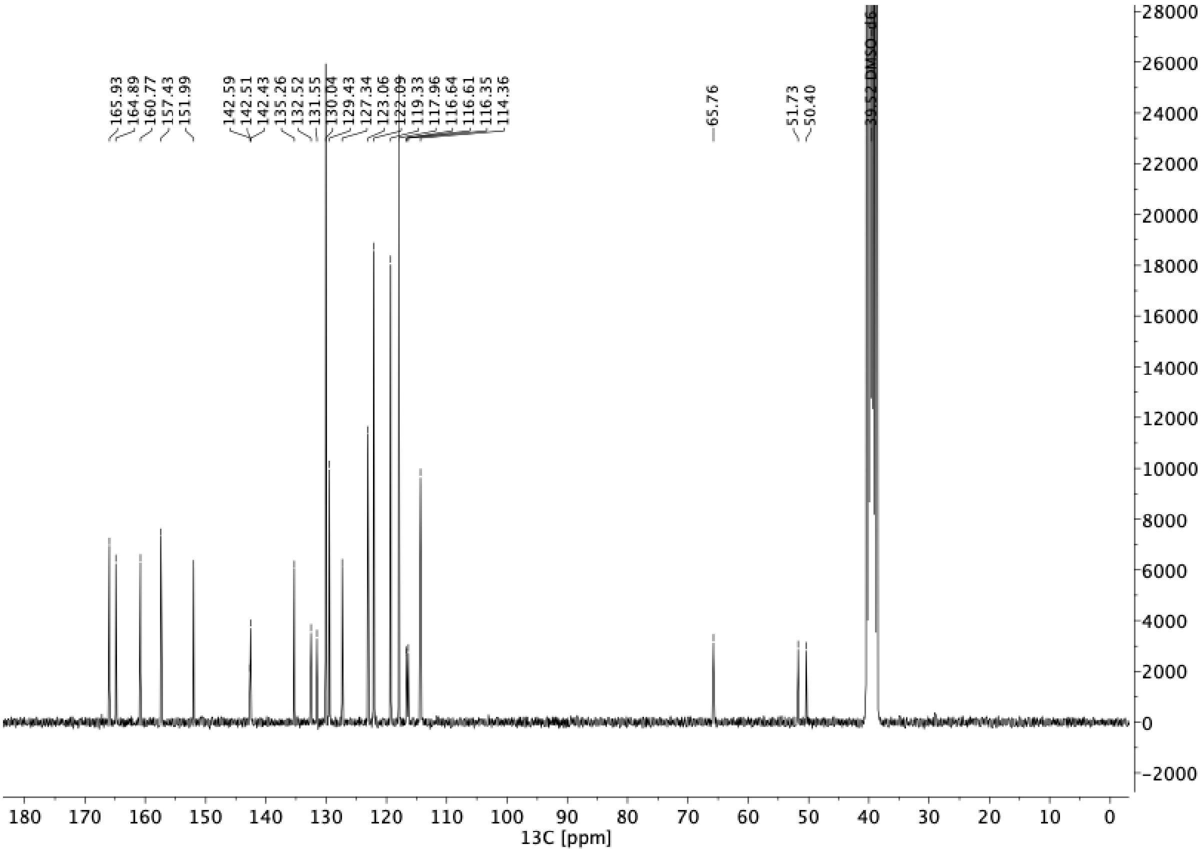

^{1}H-NMR of compound **8b**

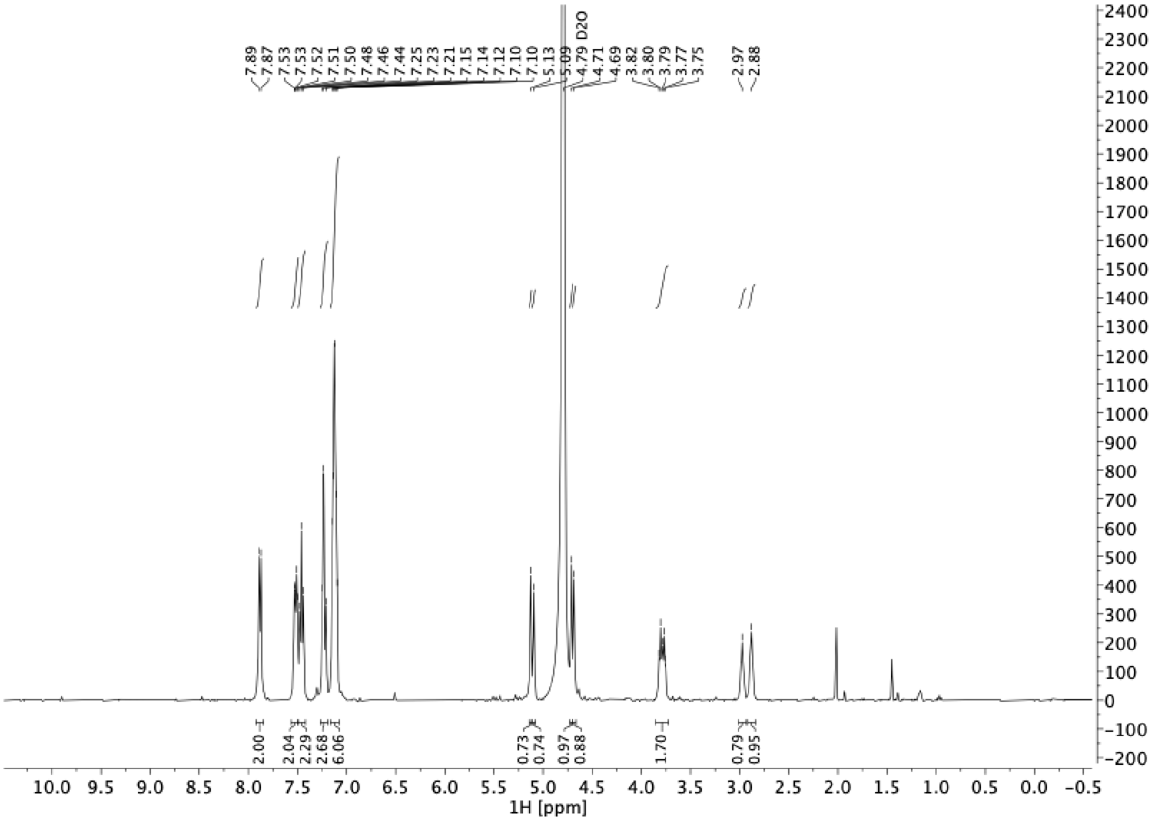

^{31}P-NMR of compound **8b**

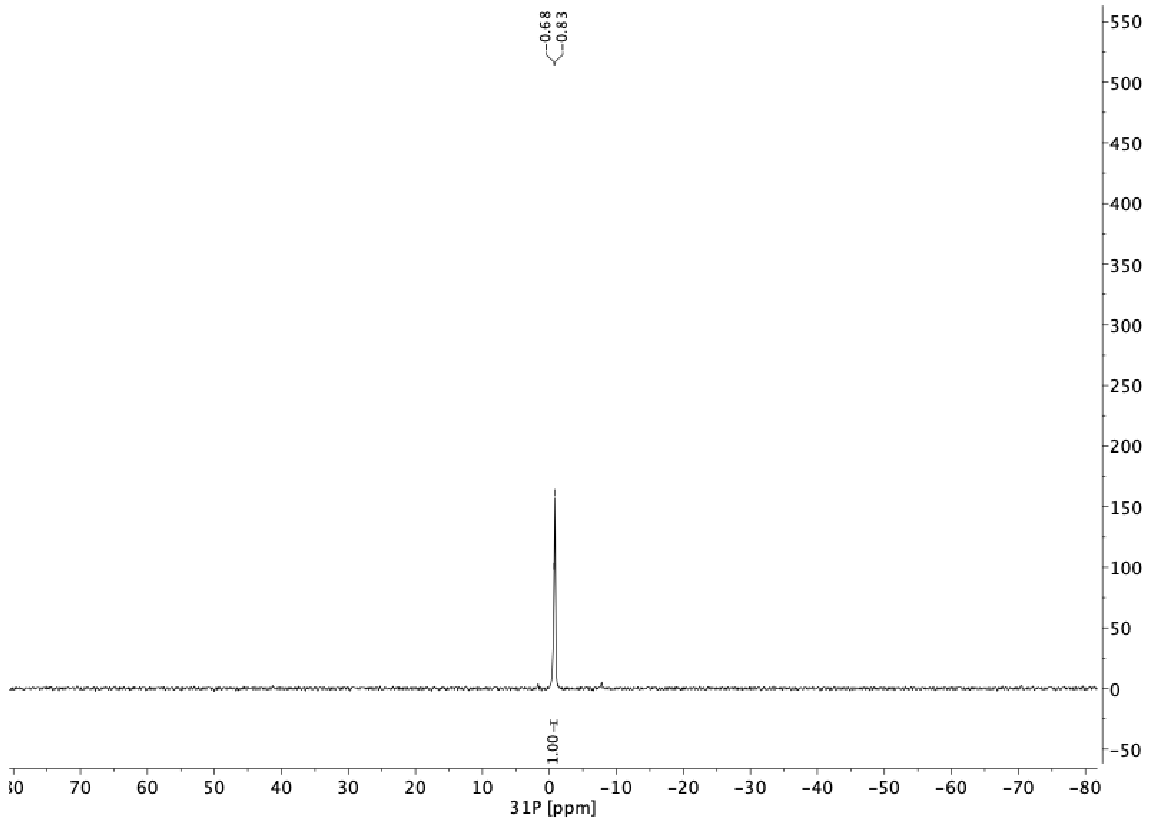

^{13}C-NMR of compound **8b**

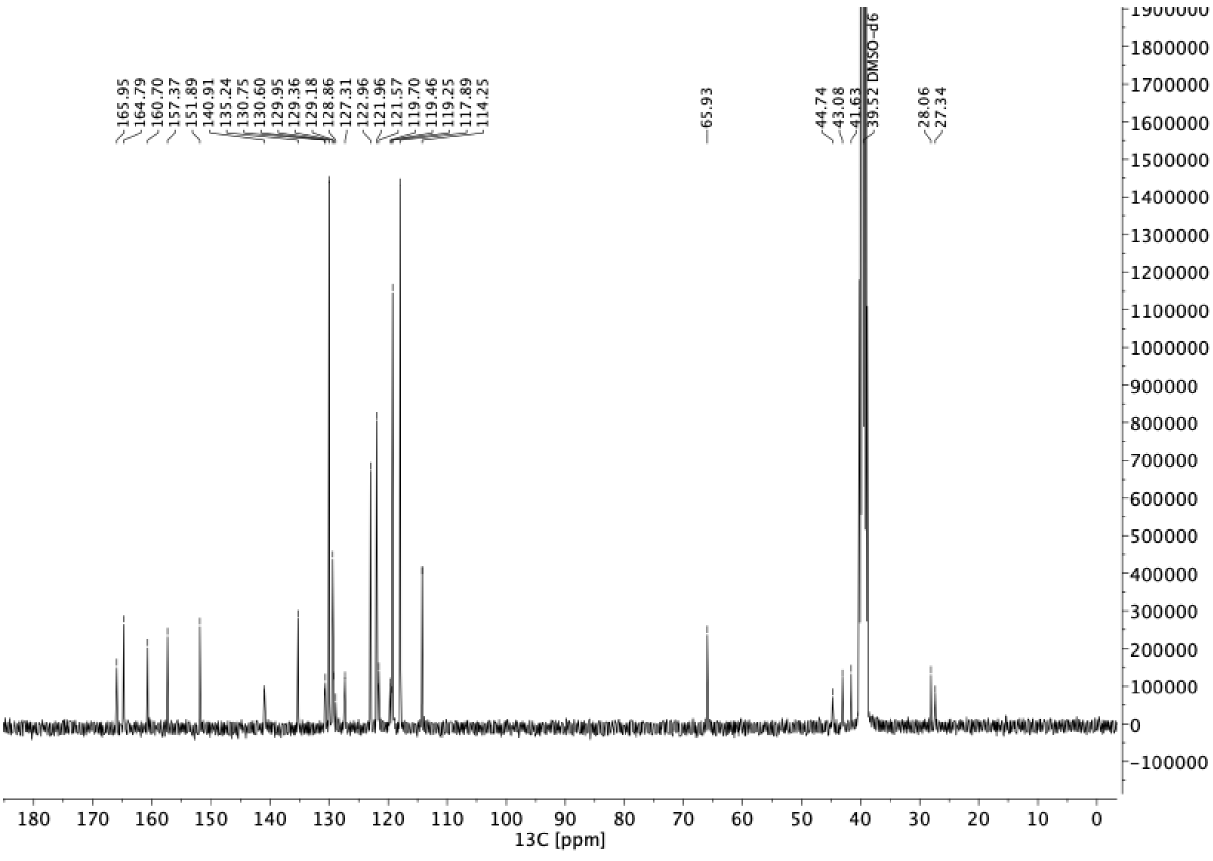

^{1}H-NMR of compound **8c**

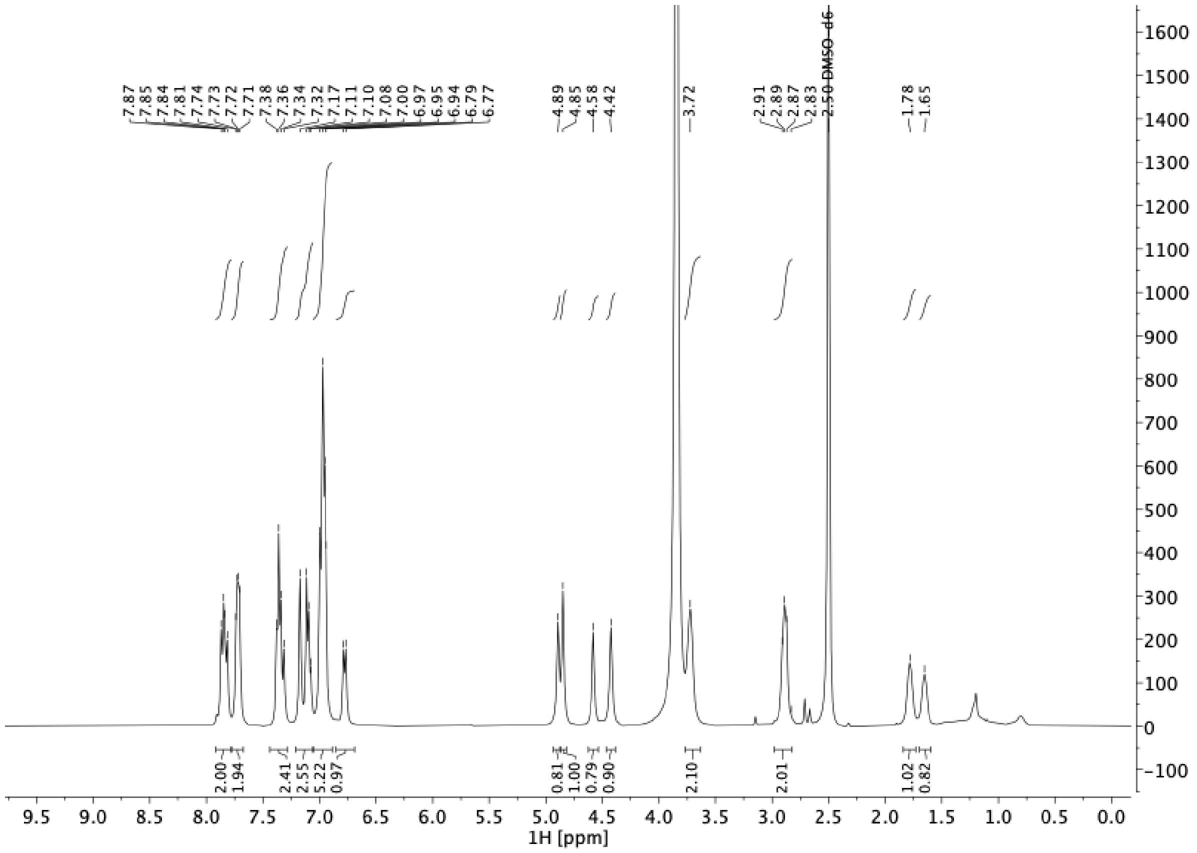

^{31}P-NMR of compound **8c**

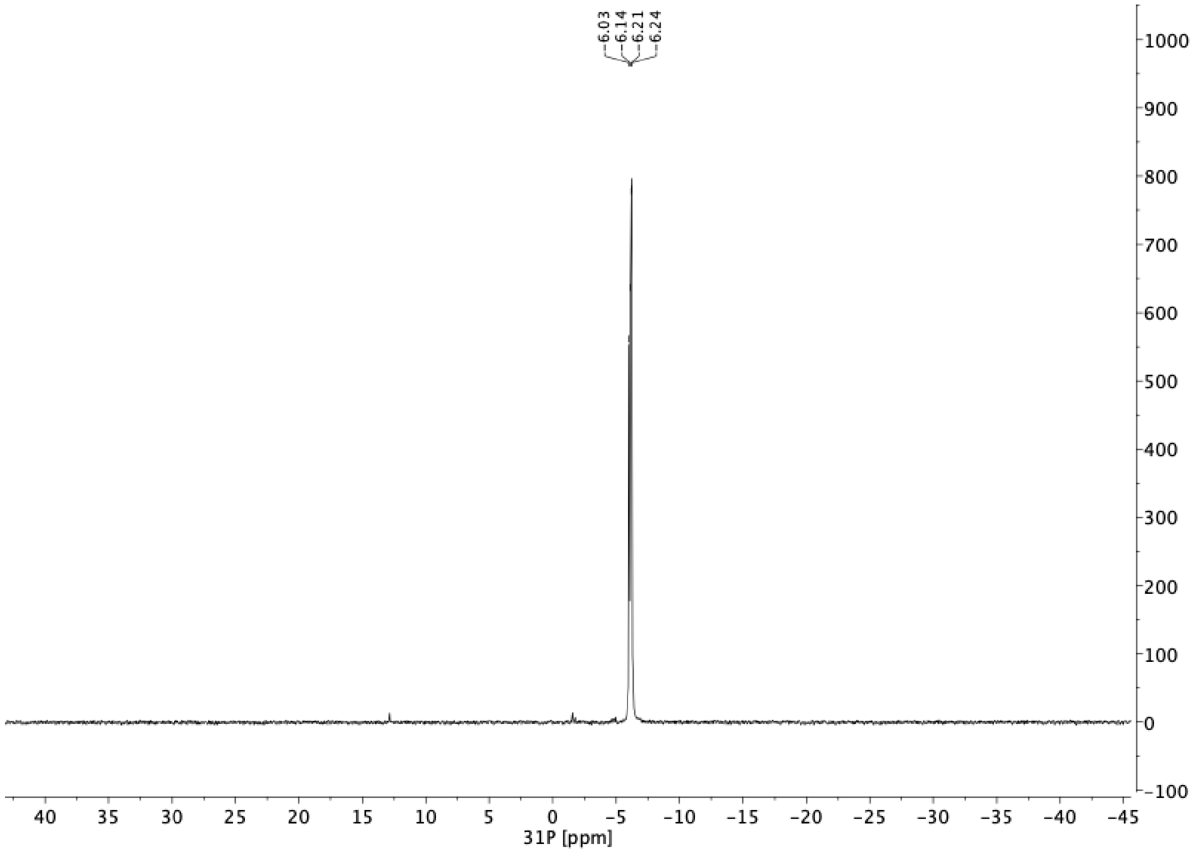

^{13}C-NMR of compound **8c**

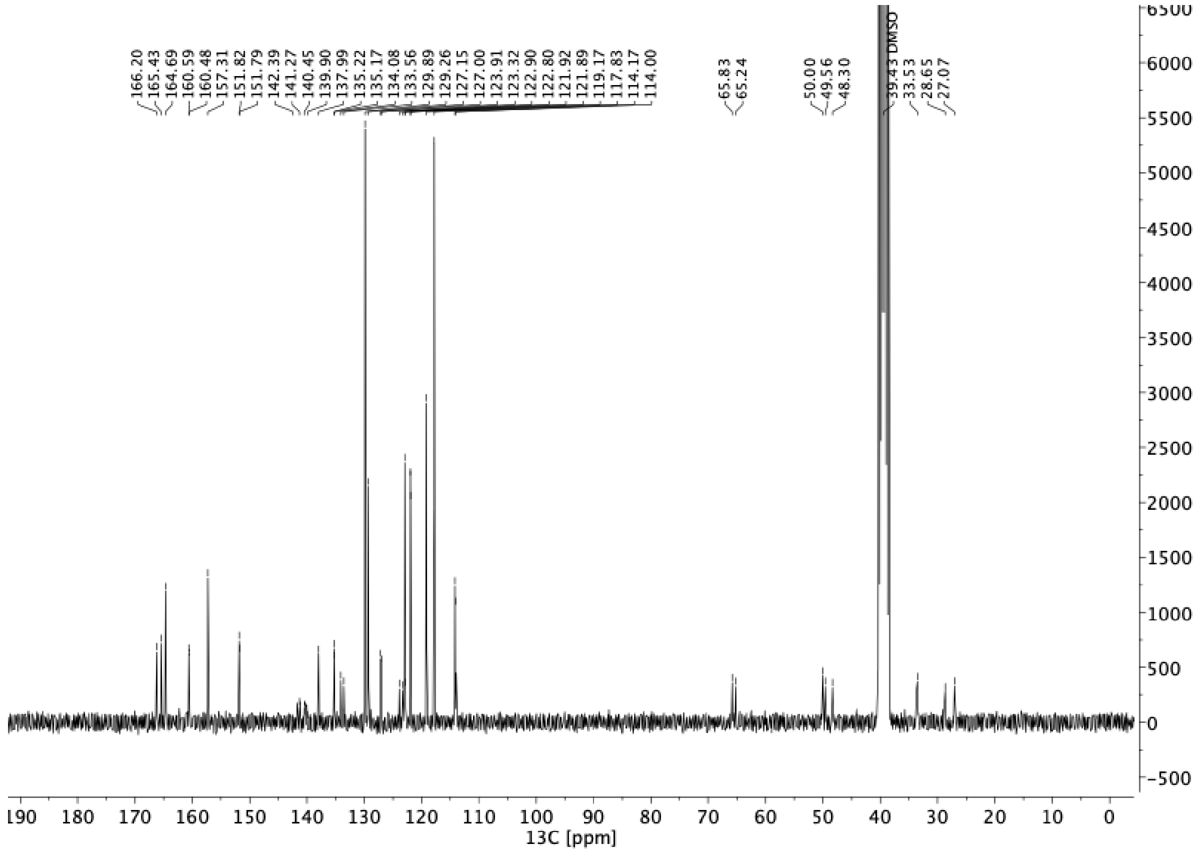

^{1}H-NMR of compound **13**

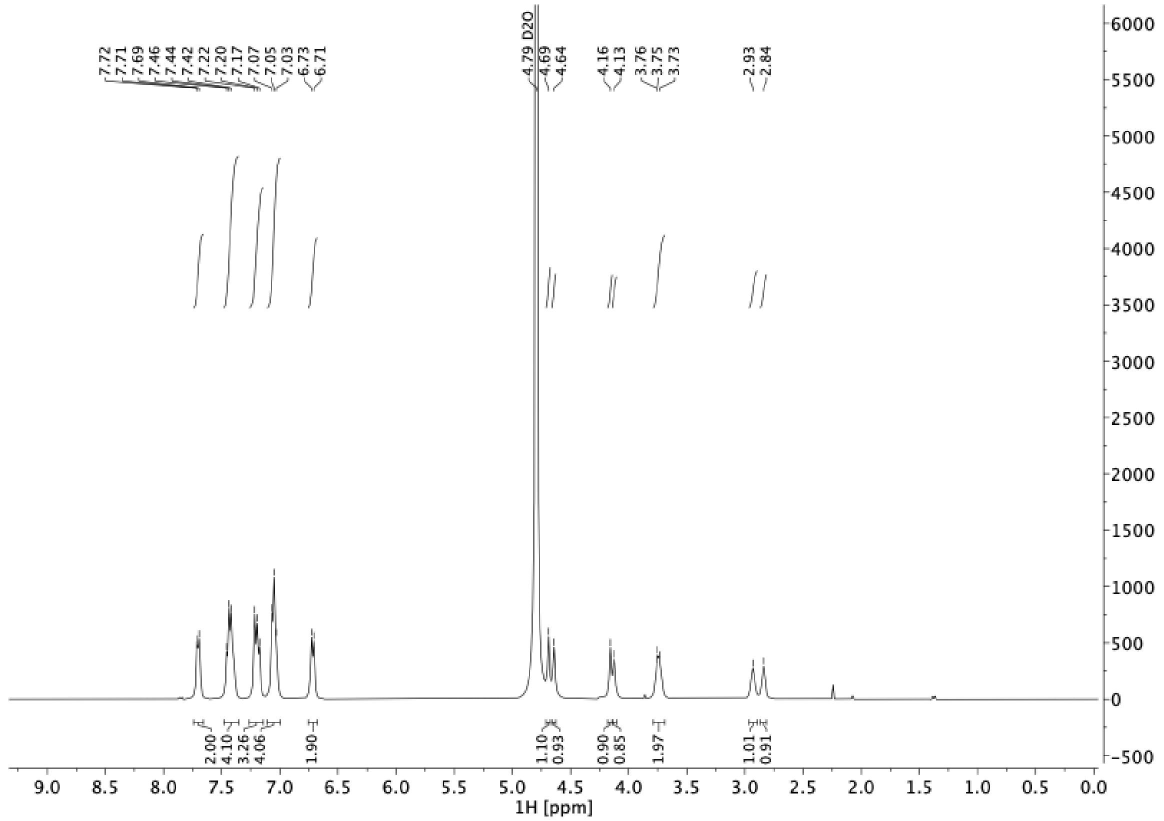

^{31}P-NMR of compound **13**

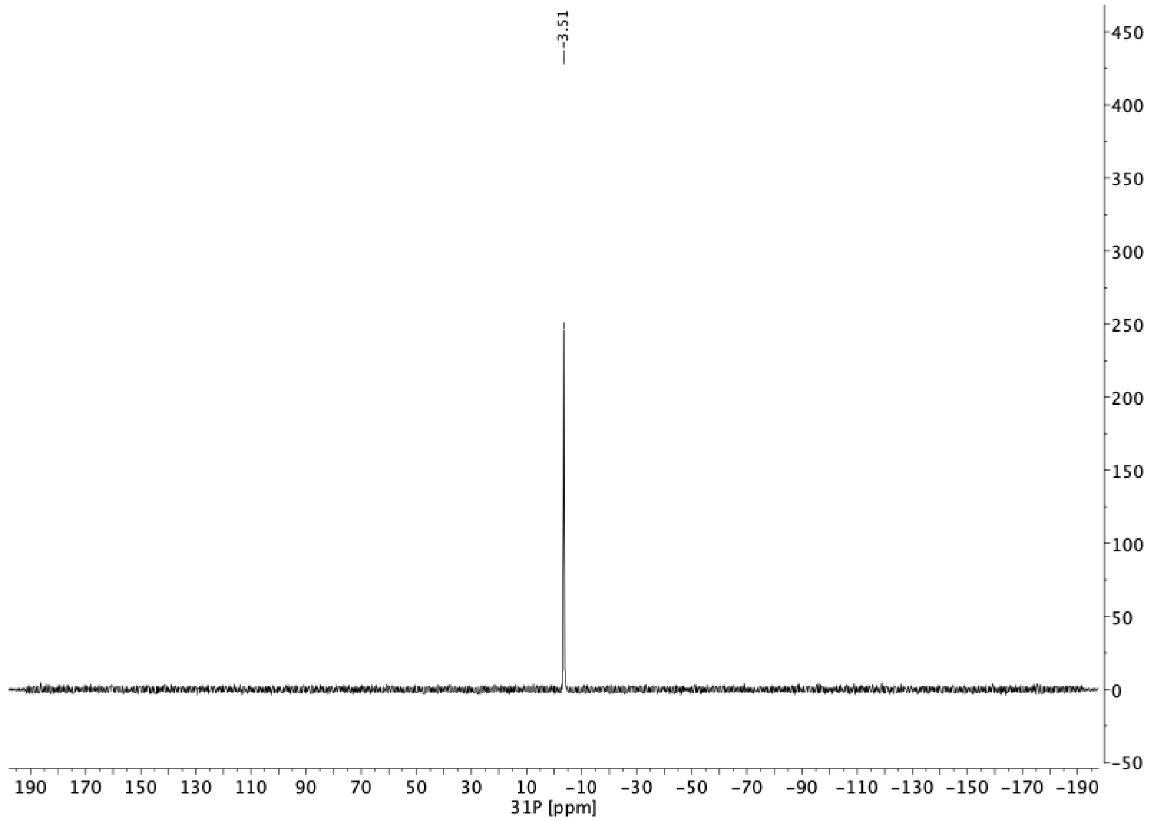

^{13}C-NMR of compound **13**

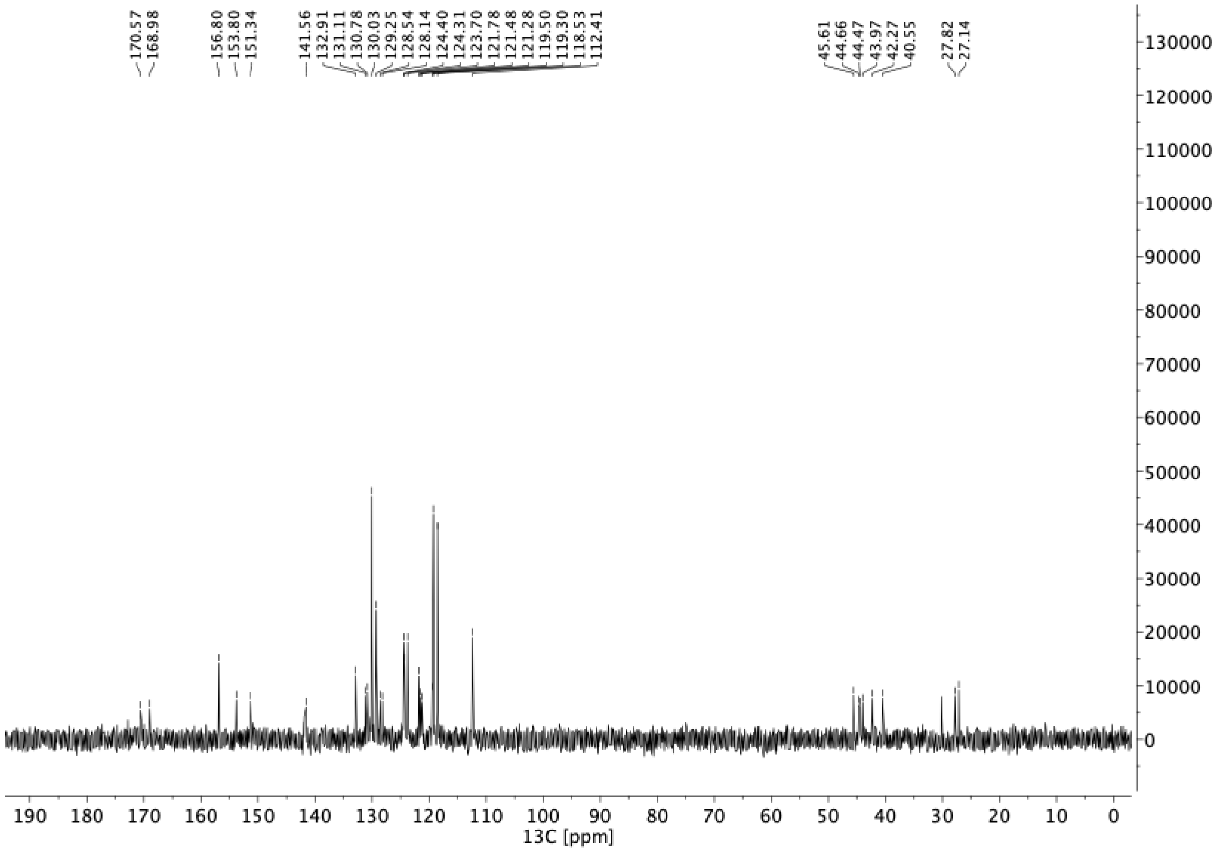

^{1}H-NMR of compound **14**

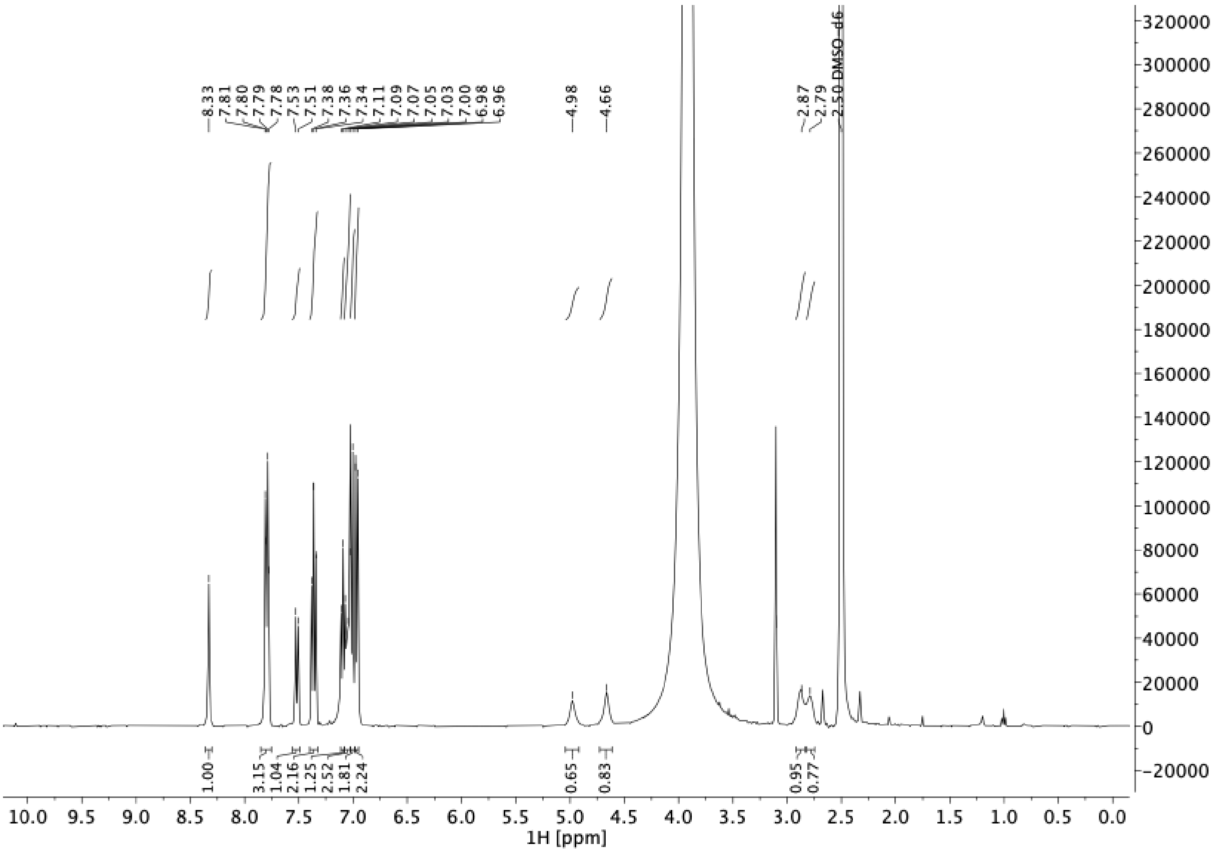

^{31}P-NMR of compound **14**

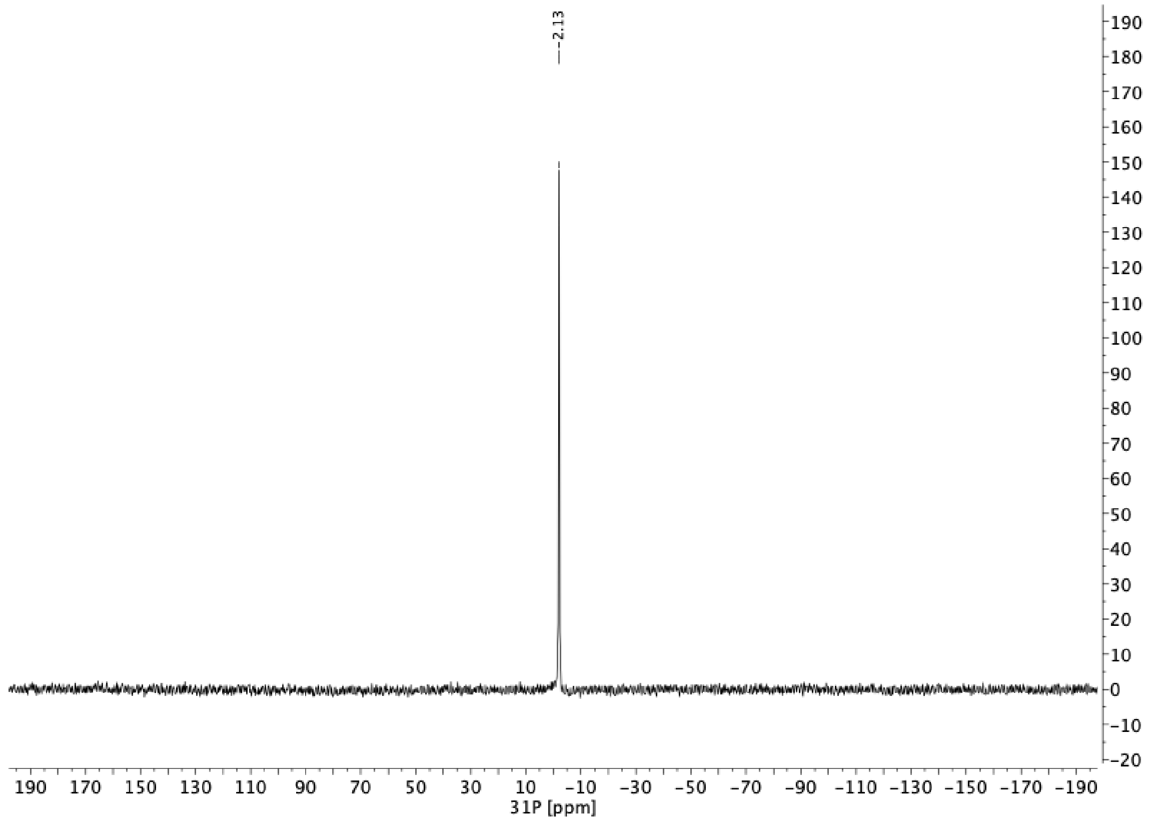

^{13}C-NMR of compound **14**

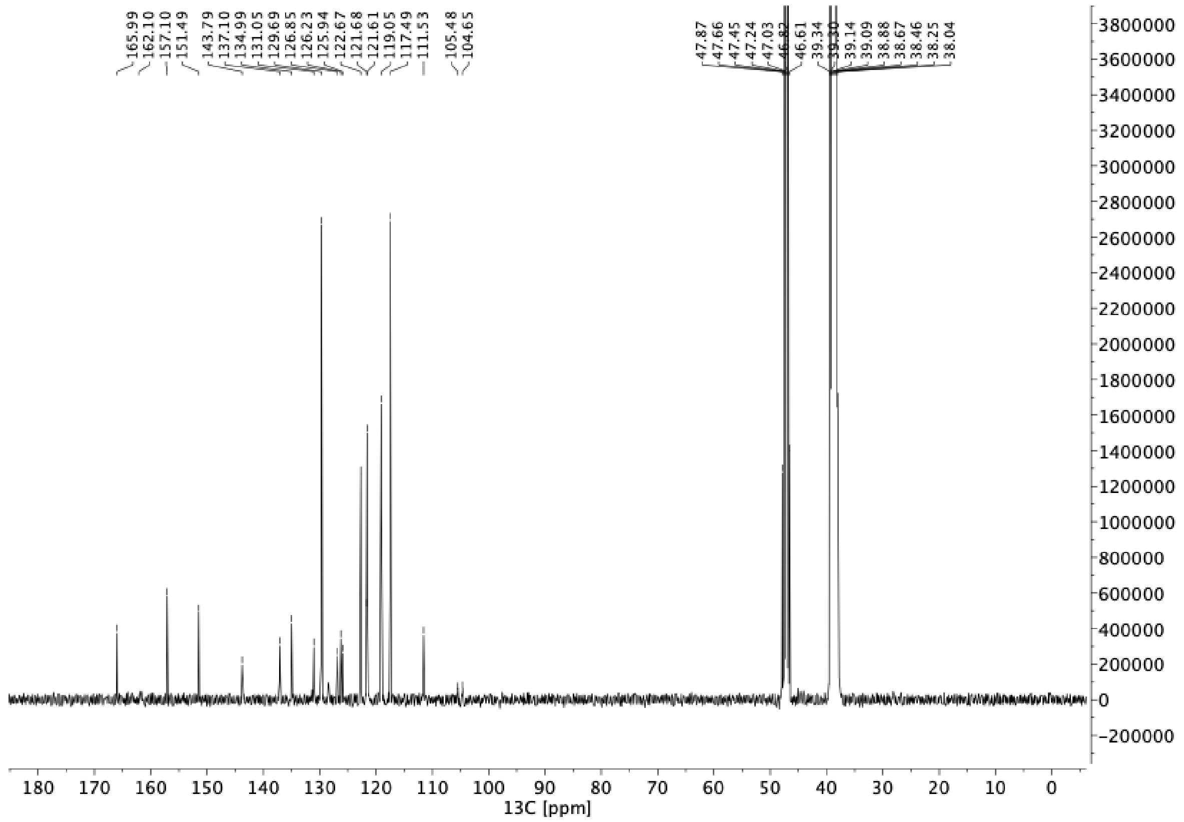

^{1}H-NMR of compound **15**

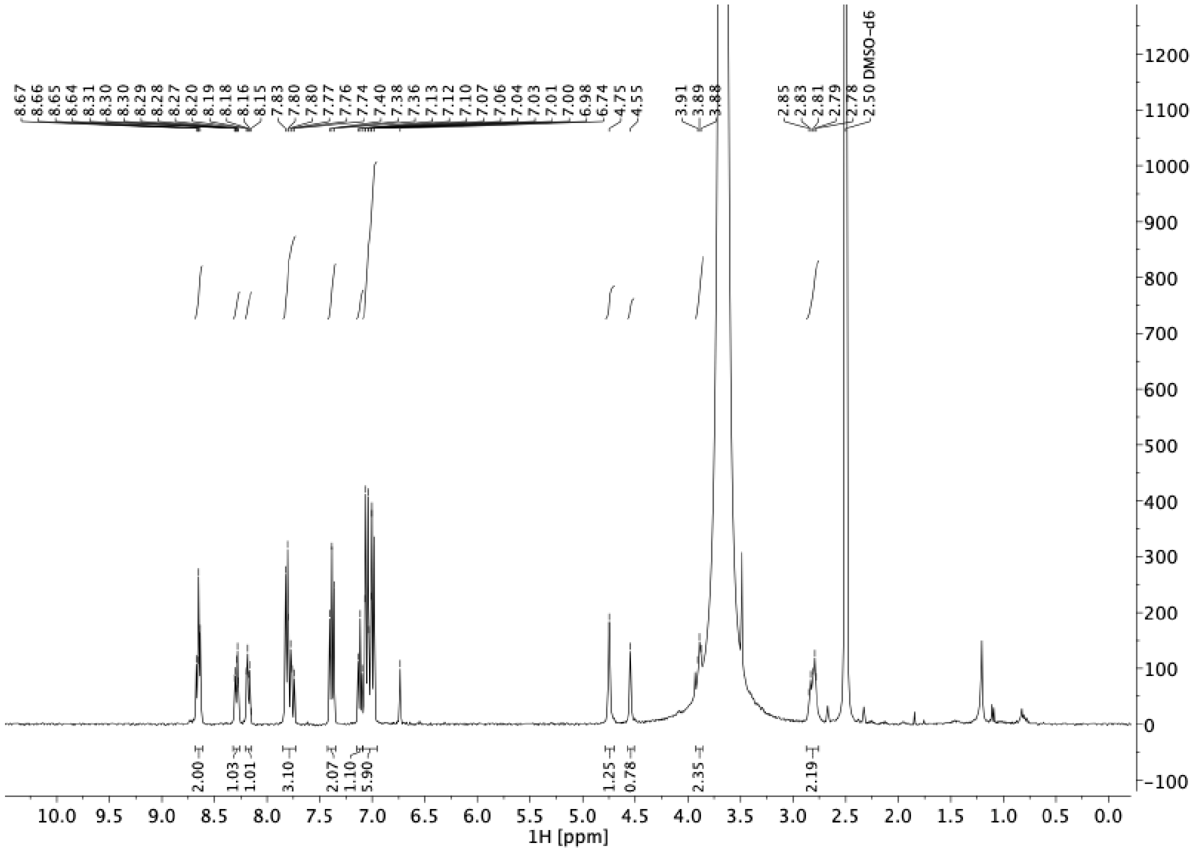

^{31}P-NMR of compound **15**

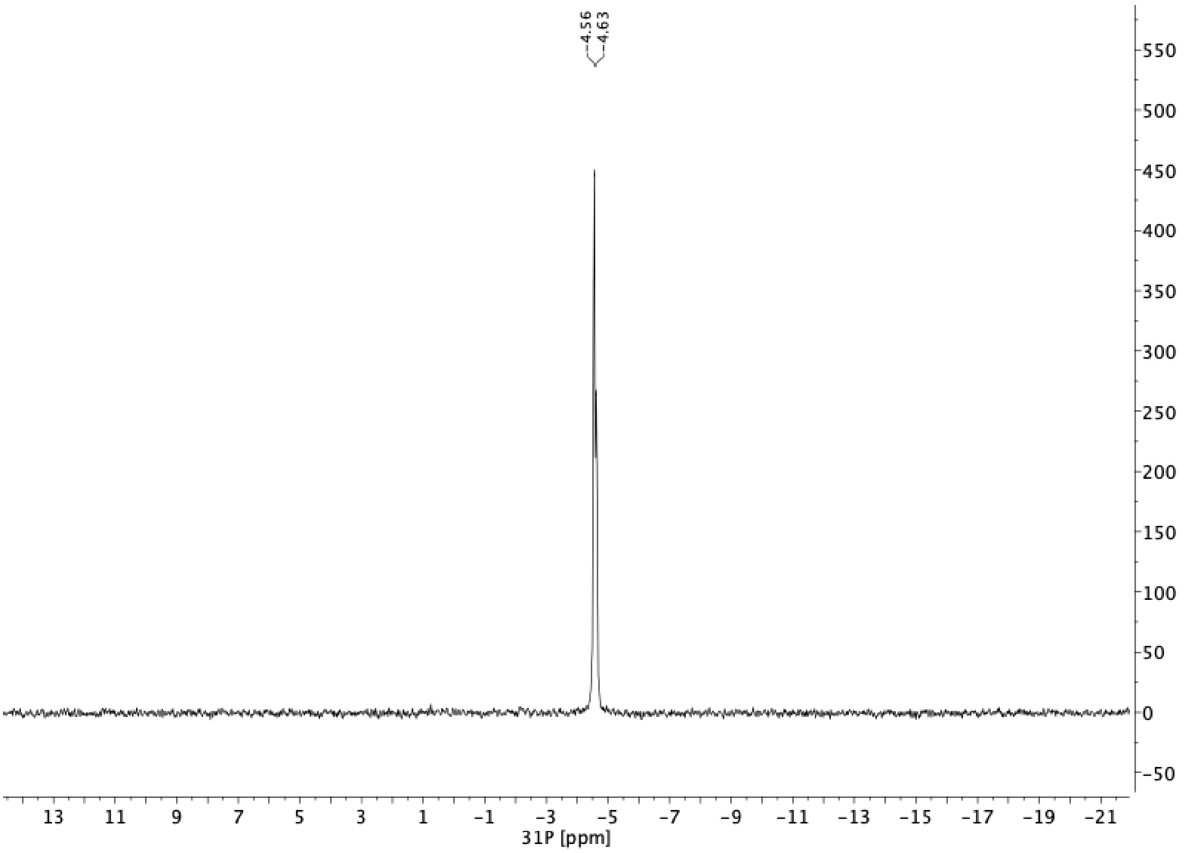

^{13}C-NMR of compound **15**

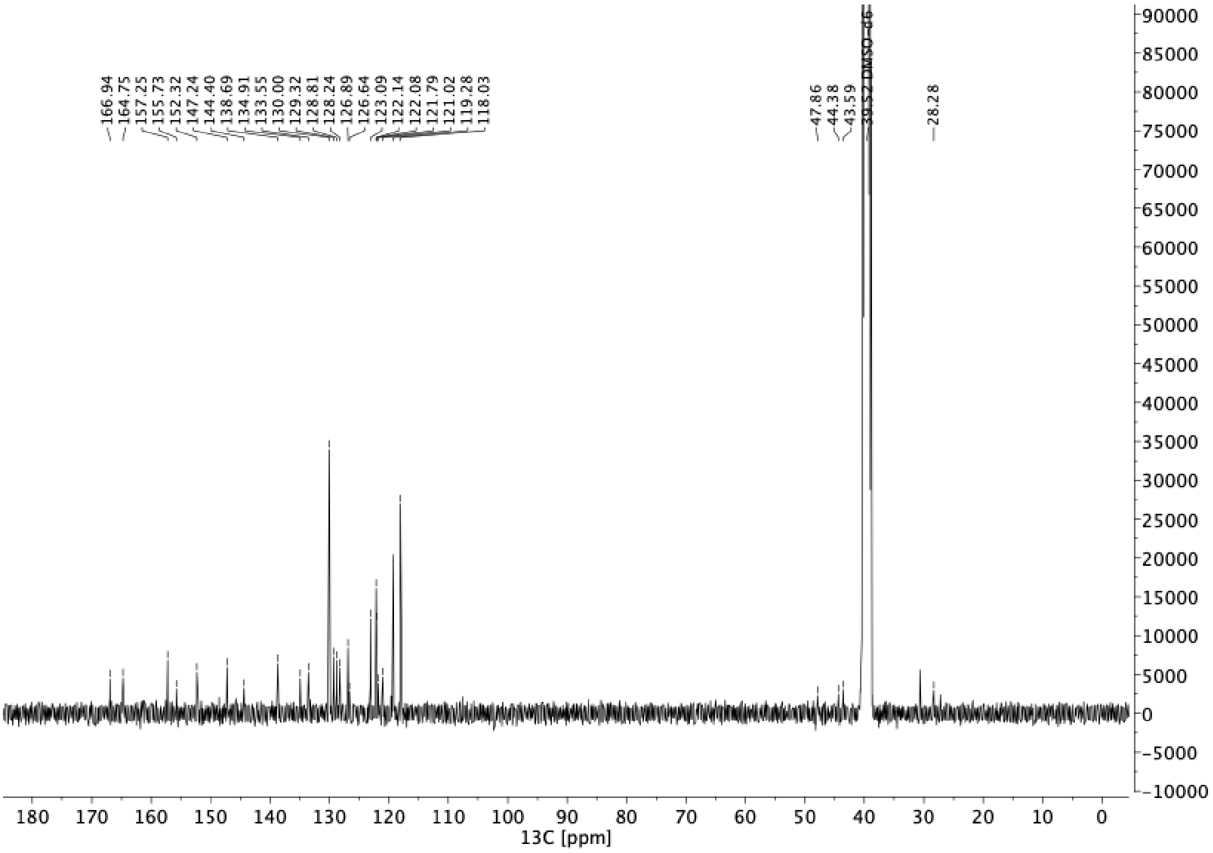

^{1}H-NMR of compound **16**

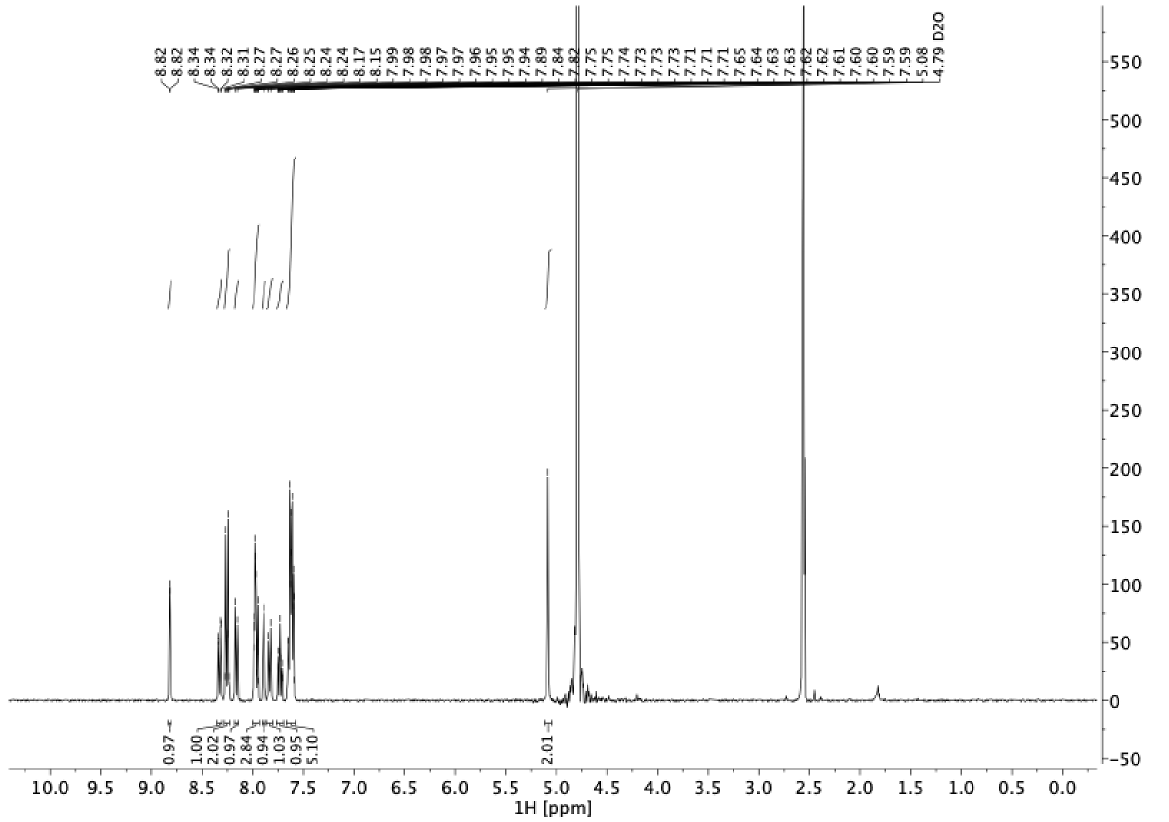

^{31}P-NMR of compound **16**

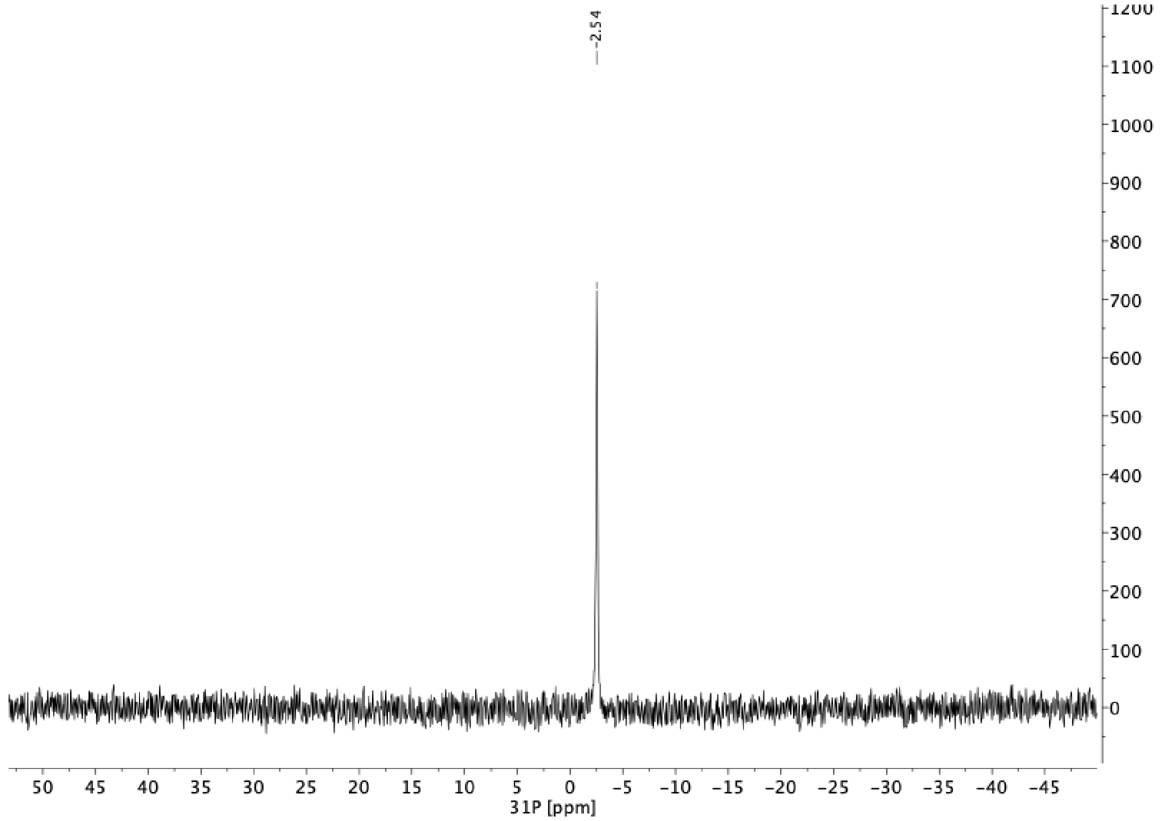

^{13}C-NMR of compound **16**

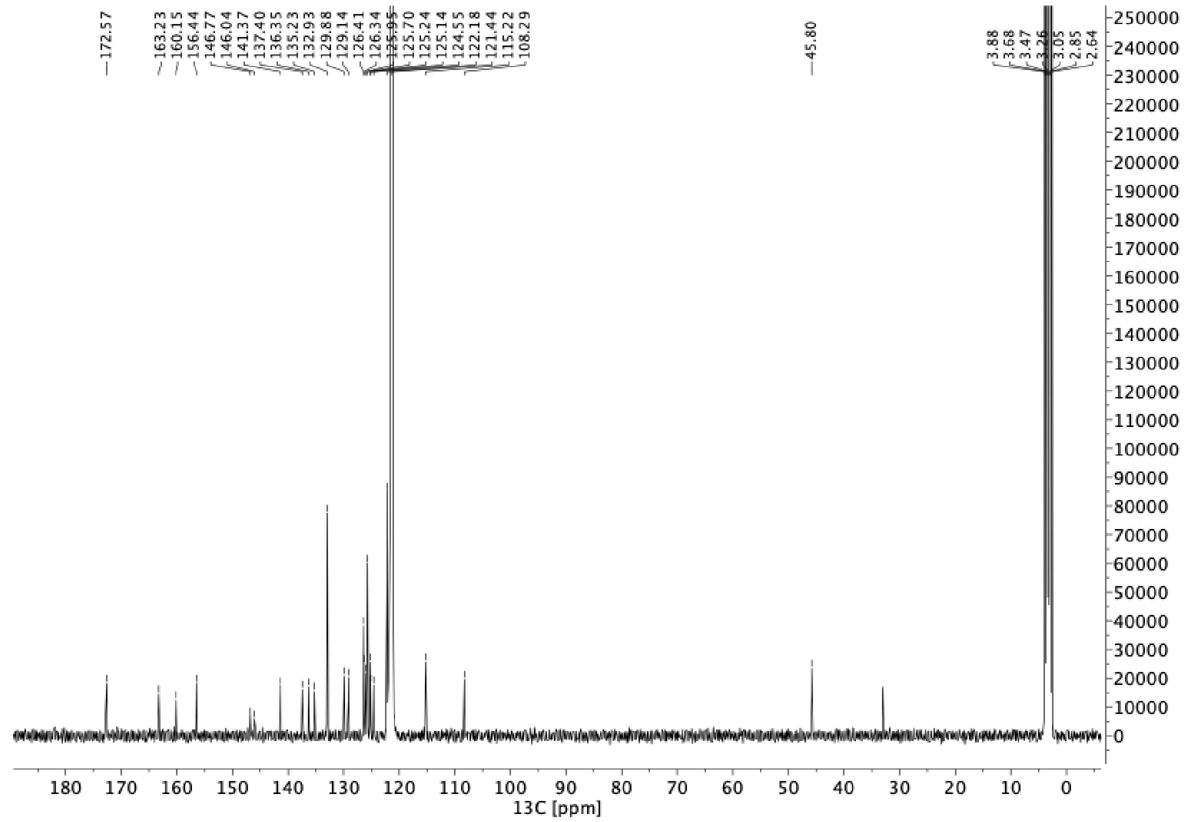

^{1}H-NMR of compound **17**

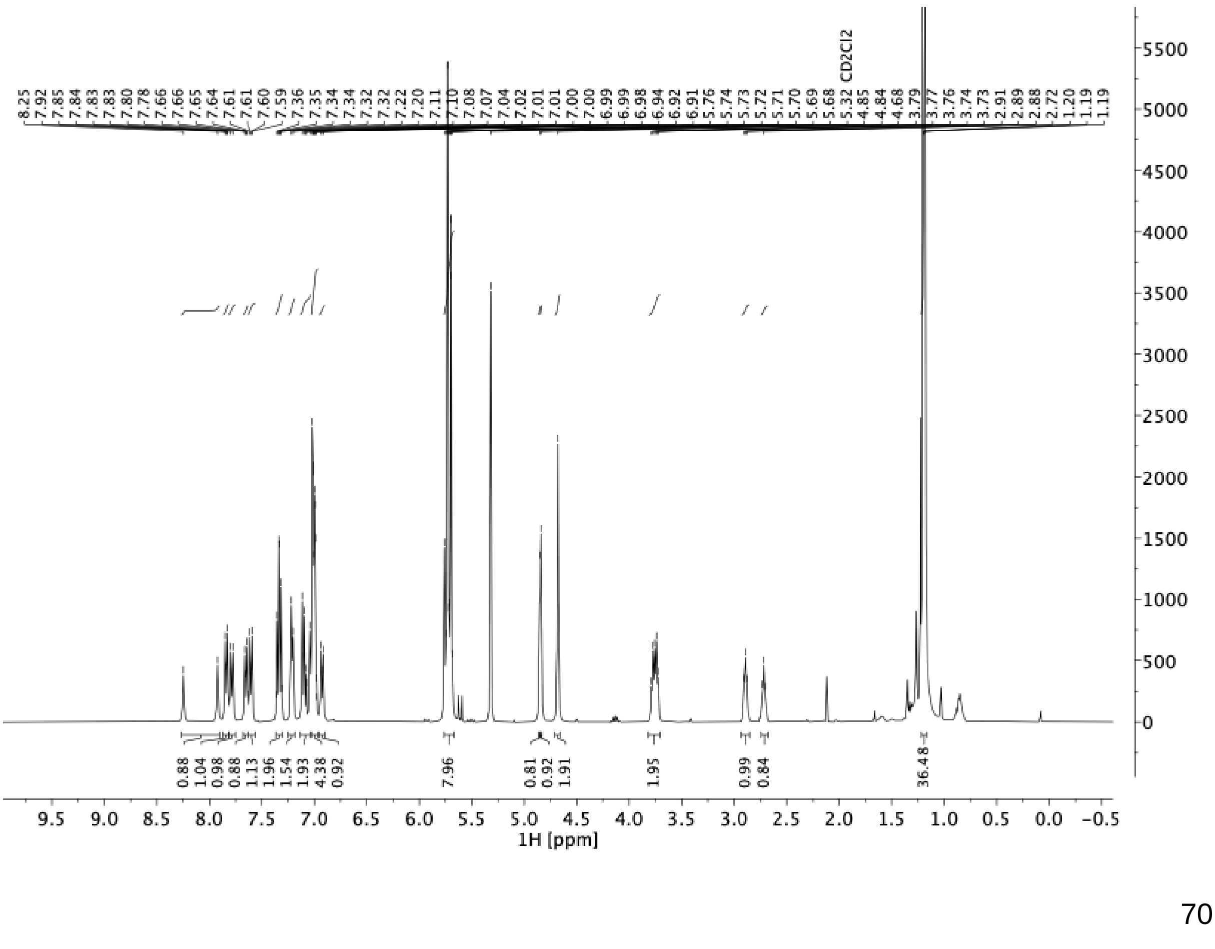

^{31}P-NMR of compound **17**

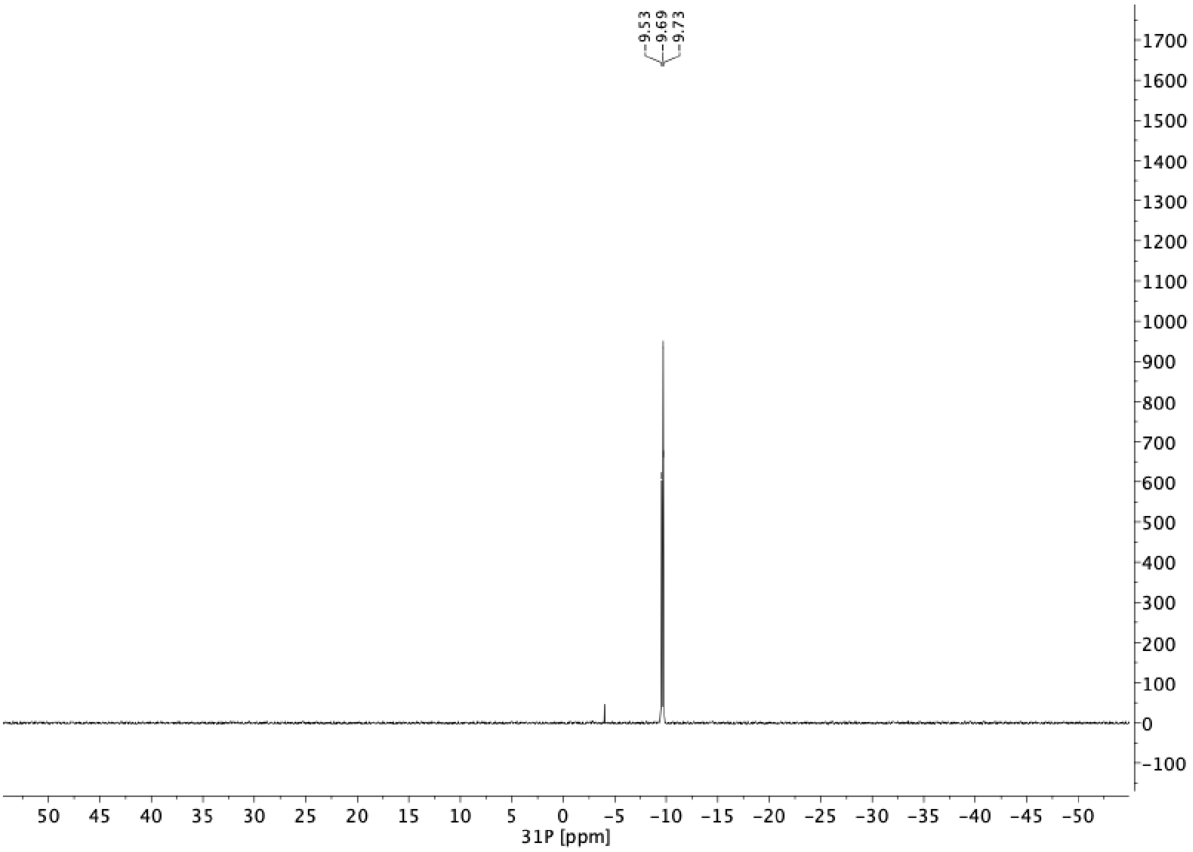

^{13}C-NMR of compound **17**

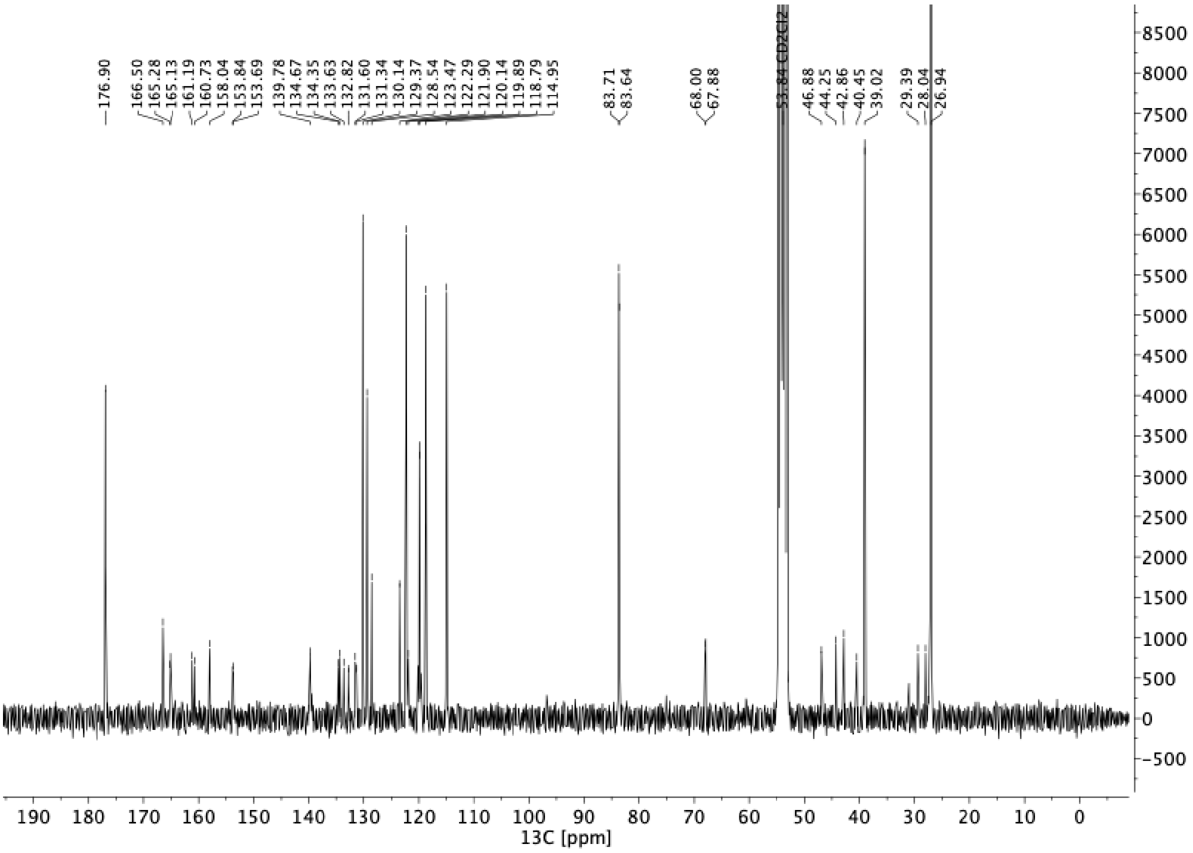

Supporting references

[1] N. Elumalai, A. Berg, S. Rubner, L. Blechschmidt, C. Song, K. Natarajan, J. Matysik, T. Berg "Rational development of Stafib-2: a selective, nanomolar inhibitor of the transcription factor STAT5b", *Sci. Rep.* **2017**, *7*, 819.

[2] J. Schust, B. Sperl, A. Hollis, T. U. Mayer, T. Berg "Stattic: a small-molecule inhibitor of STAT3 activation and dimerization", *Chem. Biol.* **2006**, *13*, 1235-1242.

[3] J. Schust, T. Berg "A high-throughput fluorescence polarization assay for signal transducer and activator of transcription 3", *Anal. Biochem.* **2004**, *330*, 114-118.

[4] M. Gräber, W. Janczyk, B. Sperl, N. Elumalai, C. Kozany, F. Hausch, T. A. Holak, T. Berg "Selective targeting of disease-relevant protein binding domains by O-phosphorylated natural product derivatives", *ACS Chem. Biol.* **2011**, *6*, 1008-1014.

[5] N. Elumalai, A. Berg, K. Natarajan, A. Scharow, T. Berg "Nanomolar Inhibitors of the Transcription Factor STAT5b with High Selectivity over STAT5a", *Angew. Chem. Int. Ed.* **2015**, *54*, 4758-4763.

[6] J. Müller, J. Schust, T. Berg "A high-throughput assay for signal transducer and activator of transcription 5b based on fluorescence polarization", *Anal. Biochem.* **2008**, *375*, 249-254.

[7] J. Gräb, A. Berg, L. Blechschmidt, B. Klüver, S. Rubner, D. Y. Fu, J. Meiler, M. Gräber, T. Berg "The STAT5b Linker Domain Mediates the Selectivity of Catechol Bisphosphates for STAT5b over STAT5a", *ACS Chem. Biol.* **2019**, *14*, 796-805.

[8] S. Keller, C. Vargas, H. Zhao, G. Piszczek, C. A. Brautigam, P. Schuck "High-precision isothermal titration calorimetry with automated peak-shape analysis", *Anal. Chem.* **2012**, *84*, 5066-5073.

[9] T. H. Scheuermann, C. A. Brautigam "High-precision, automated integration of multiple isothermal titration calorimetric thermograms: new features of NITPIC", *Methods* **2015**, *76*, 87-98.

[10] J. C. D. Houtman, P. H. Brown, B. Bowden, H. Yamaguchi, E. Appella, L. E. Samelson, P. Schuck "Studying multisite binary and ternary protein interactions by global analysis of isothermal titration calorimetry data in SEDPHAT: Application to adaptor protein complexes in cell signaling", *Protein Sci.* **2007**, *16*, 30-42.

[11] C. A. Brautigam, in *Methods Enzymol., Vol. 562* (Ed.: J. L. Cole), Academic Press, **2015**, pp. 109-133.

[12] C. A. Schneider, W. S. Rasband, K. W. Eliceiri "NIH Image to ImageJ: 25 years of image analysis", *Nat. Methods* **2012**, *9*, 671-675.

[13] J. A. Grzyb, M. Shen, C. Yoshina-Ishii, W. Chi, R. S. Brown, R. A. Batey "Carbamoylimidazolium and thiocarbamoylimidazolium salts: novel reagents for the

synthesis of ureas, thioureas, carbamates, thiocarbamates and amides", *Tetrahedron* **2005**, *61*, 7153-7175.

[14] P. R. Brooks, M. C. Wirtz, M. G. Vetelino, D. M. Rescek, G. F. Woodworth, B. P. Morgan, J. W. Coe "Boron Trichloride/Tetra-n-Butylammonium Iodide: A Mild, Selective Combination Reagent for the Cleavage of Primary Alkyl Aryl Ethers", *J. Org. Chem.* **1999**, *64*, 9719-9721.

[15] J. In, S. Hwang, C. Kim, J. H. Seo, S. Kim "Synthesis of 3,4-Dihydroisoquinolin-1-ones from N-Boc-(β-Arylethyl)carbamates via Isocyanate Intermediates", *Eur. J. Org. Chem.* **2013**, *2013*, 965-971.

[16] T. Laykea, J. K. Donald "An Efficient Parallel Synthesis of Capsazepine and Capsazepine Analogs", *Comb. Chem. High Throughput Screen.* **2004**, *7*, 153-161.

[17] H. Zheng, Y. Dong, L. Li, B. Sun, L. Liu, H. Yuan, H. Lou "Novel Benzo[a]quinolizidine Analogs Induce Cancer Cell Death through Paraptosis and Apoptosis", *J. Med. Chem.* **2016**, *59*, 5063-5076.

[18] K. S. Eckhardt, T. Münzel, J. Gräb, T. Berg "Stafiba: A STAT5-Selective Small-Molecule Inhibitor", *ChemBioChem* **2023**, *24*, e202200553.

[19] D. Jerchel, J. Heider, H. Wagner "Untersuchungen zur Reaktivitat von Alkylgruppen heterocyclischer Verbindungen und Ihrer funktionellen Derivate II. Oxydation von Alkyl-Pyridinen und Alkyl-Chinolinen durch Selendioxyd", *Liebigs Ann. Chem.* **1958**, *613*, 153-170.
